# A Genome-wide Association Study of Dupuytren Disease Reveals 17 Additional Variants Implicated in Fibrosis

**DOI:** 10.1016/j.ajhg.2017.08.006

**Published:** 2017-09-07

**Authors:** Michael Ng, Dipti Thakkar, Lorraine Southam, Paul Werker, Roel Ophoff, Kerstin Becker, Michael Nothnagel, Andre Franke, Peter Nürnberg, Ana Isabel Espirito-Santo, David Izadi, Hans Christian Hennies, Jagdeep Nanchahal, Eleftheria Zeggini, Dominic Furniss

**Affiliations:** 1 Nuffield Department of Orthopaedics, Rheumatology, and Musculoskeletal Science, University of Oxford, Botnar Research Centre, Windmill Road, Oxford OX3 7HE, UK; 2 Wellcome Trust Sanger Institute, Wellcome Genome Campus, Hinxton, Cambridge CB10 1SA, UK; 3 Wellcome Trust Centre for Human Genetics, University of Oxford, Oxford OX3 7BN, UK; 4 University of Groningen, University Medical Centre Groningen, Department of Plastic Surgery, Hanzeplein 1, 9713 GZ Groningen, the Netherlands; 5 UCLA Center for Neurobehavioral Genetics, 695 Charles E. Young Drive South, Los Angeles, CA 90095, USA; 6 Cologne Center for Genomics, University of Cologne, Weyertal 115b, 50931 Köln, Germany; 7 Cluster of Excellence on Cellular Stress Responses in Aging-associated Diseases, University of Cologne, 50931 Köln, Germany; 8 Institute of Clinical Molecular Biology, Christian-Albrechts-University of Kiel, University Hospital Schleswig-Holstein, 24105 Kiel, Germany; 9 Department of Biological Sciences, University of Huddersfield, Huddersfield HD1 3DH, UK; 10 Department of Plastic and Reconstructive Surgery, Oxford University Hospitals NHS Foundation Trust, John Radcliffe Hospital, Oxford OX3 9DU, UK; 11 NIHR Biomedical Research Centre, NDORMS, University of Oxford, Botnar Research Centre, Windmill Road, Oxford OX3 7HE, UK

**Keywords:** Dupuytren disease, fibrosis, genetics, GWAS, hand surgery

## Abstract

Individuals with Dupuytren disease (DD) are commonly seen by physicians and surgeons across multiple specialties. It is an increasingly common and disabling fibroproliferative disorder of the palmar fascia, which leads to flexion contractures of the digits, and is associated with other tissue-specific fibroses. DD affects between 5% and 25% of people of European descent and is the most common inherited disease of connective tissue. We undertook the largest GWAS to date in individuals with a surgically validated diagnosis of DD from the UK, with replication in British, Dutch, and German individuals. We validated association at all nine previously described signals and discovered 17 additional variants with p ≤ 5 × 10^−8^. As a proof of principle, we demonstrated correlation of the high-risk genotype at the statistically most strongly associated variant with decreased secretion of the soluble WNT-antagonist SFRP4, in surgical specimen-derived DD myofibroblasts. These results highlight important pathways involved in the pathogenesis of fibrosis, including WNT signaling, extracellular matrix modulation, and inflammation. In addition, many associated loci contain genes that were hitherto unrecognized as playing a role in fibrosis, opening up new avenues of research that may lead to novel treatments for DD and fibrosis more generally. DD represents an ideal human model disease for fibrosis research.

## Introduction

Dupuytren disease (DD [MIM: 126900]) is a progressive fibroproliferative disease of the palmar fascia and the most common inherited disorder of the connective tissue. It is the most frequent example of a tissue-specific fibrotic disease: others include pulmonary, renal, hepatic, and skin fibrosis. It is accepted that there are common features of all fibrotic diseases, but some pathologic pathways are likely to be tissue specific.^1^

DD is characterized by the initial development of myofibroblast-rich nodules in the palm of the hand. These myofibroblasts express alpha-smooth muscle actin (α-SMA) and secrete types III and I collagen, leading to the formation of abnormal cords in the palm of the hand. In a proportion of people with DD, the myofibroblasts cause contraction, leading to flexion contractures of the involved digits and subsequent functional impairment.^2^, ^3^ As the hand is the sensorimotor end-organ of the upper limb, impairment here has a disproportionate effect on the quality of life of the individual.^4^ Additionally, because DD is associated with other forms of fibrosis, it may serve as an ideal human model system for fibrotic disease, and the routine excision of tissue as a part of treatment facilitates experimental medicine studies.^5^

DD is very common, affecting 5%–25% of people in populations of European descent, and there is evidence that the prevalence is increasing.^6^, ^7^, ^8^ The mainstay of treatment for DD is surgery, though newer modalities are increasing in popularity. Despite this, complications and recurrence of disease are both common, even after adequate primary treatment.^9^, ^10^

DD has a substantial heritable component. A twin study from Denmark estimated the heritability of DD at 80%^11^ and a sibling recurrence study from the UK estimated the *λ*_*S*_ to be 4.48, confirming a strong genetic predisposition to DD.^12^ Furthermore, age at first surgical intervention is significantly younger in those with a positive family history.^13^ Similarly, there is evidence that multiple non-genetic factors, such as smoking, alcohol intake, diabetes, and hyperlipidemia, also play a role in disease development.^14^

We have previously undertaken a pilot GWAS in 960 Dutch DD-affected individuals to begin to delineate the common genetic variation underlying this predisposition. This defined nine susceptibility loci and revealed the hitherto unsuspected importance of components of the WNT signaling pathway in the pathogenesis of DD.^15^ To boost power for the detection of common-frequency signals, here we undertook a 4-fold larger GWAS in 3,871 UK individuals with surgically validated DD. Replication of significant and suggestive loci was performed in a total of 4,041 surgically validated DD-affected case subjects from the UK, the Netherlands, and Germany.

## Material and Methods

### Ethical Approval

The study was approved by the Research Ethics Committee or equivalent at all institutions where the work was carried out: Oxfordshire Research Ethics Committee B/09/H0605/65 for the British Society for Surgery of the Hand Genetics of Dupuytren’s Disease (BSSH-GODD) study (UK), Medical Ethics Committee (METc) 2007/067 for the Genetic Origin of Dupuytren Disease (GODDAF) Study (the Netherlands), and University of Cologne 14/292 for the German Dupuytren Study (Germany). Informed consent was obtained from all subjects.

### Phenotype Definition and Study Populations

We used samples from three European countries for this study. In all cohorts, the DD-affected case subjects were individuals who had undergone surgical treatment for their disease. The UK cohort consisted of a total of 5,408 case subjects from the BSSH-GODD Study and 9,961 population-based control subjects from the United Kingdom Household Longitudinal Study (UKHLS), which were divided into 4,891 control subjects for the discovery phase and 5,070 control subjects for the replication phase. The Dutch cohort consisted of 2,195 case subjects from the GODDAF Study and 1,983 control subjects from the Lifelines cohort study. The German cohort consisted of 768 case subjects from the German Dupuytren Study and 1,353 control subjects from the PopGen and KORA studies. The cohorts included all samples analyzed in our previous GWAS.^15^

### Biological Samples

For the BSSH-GODD cohort, salivary samples were collected using the Oragene-OG250 salivary DNA collection kit (DNA Genotek). DNA was extracted according to manufacturer’s instructions and stored at −80°C. Diseased fascial samples removed at surgery were immediately placed in EMEM media (Lonza) and transferred by overnight courier to our laboratory.

The UK Household Longitudinal Study is a stratified clustered random sample of households representative of the UK population, led by the Institute for Social and Economic Research at the University of Essex and funded by the Economic and Social Research Council. Blood was taken and DNA isolated by standard methods. The genome-wide scan data were analyzed and deposited by the Wellcome Trust Sanger Institute. Information on how to access the data can be found on the Understanding Society website.

For the GODDAF study, case subjects were identified from plastic surgery clinics within the Netherlands, and DNA and phenotype data were obtained as previously described.^15^

LifeLines is a population-based cohort study based in the Netherlands and has been previously described.^16^ For the purpose of this study, DNA samples from participants, age- and sex-matched to the GODDAF case subjects, were isolated (project number OV14_0257).

For the German Dupuytren study cohort, blood samples were collected from case subjects from Germany and Switzerland by the German Dupuytren Study Group^13^ and DNA was extracted with standard procedures. 1,353 control subjects were obtained from the Popgen and KORA studies.

### Genotyping, Association Analysis, and Imputation

We genotyped 4,201 UK DD-affected case subjects using Illumina HumanCoreExome arrays at the Wellcome Trust Sanger Institute comprising 538,448 SNPs. The data were called using the Illumina GenCall algorithm. Quality control and association analyses were performed in PLINK v.1.9 and R v.3.3.1. We initially performed sample-level quality control (Figure S1). Briefly, we first removed all SNPs with a call rate < 90%. We standardized the output data to NCBI build 37 (hg19) and the strand alignment using scripts provided by Dr. William Rayner. We then removed 242 samples with one or more of the following properties: call rate < 98%; heterozygosity > 3 standard deviations from the mean; different genotype-derived sex and reported sex; or failure of genotyped SNPs to match the pre-GWAS Sequenom fingerprinting. We merged our data with publically available data from the 1000 Genomes Project and performed principal components analysis (PCA) to define (and remove from further analysis) those people who were ethnic outliers by visual inspection (Figure S2).

We then performed SNP-level quality control on this sample set. Briefly, we excluded SNPs with call rate < 98%, those with Hardy-Weinberg equilibrium (HWE) p < 0.0001, and those with a cluster separation score of < 0.4. We also removed non-autosomal SNPs and those that were duplicated.

This generated a final set of 3,959 case subjects genotyped at 494,982 SNPs. From the UKHLS control subjects we selected 4,891 individuals genotyped at 525,314 SNPs after identical quality control. We then combined these control subjects with our case subjects. From this combined dataset, we further excluded 86 case subjects and 202 control subjects from a total of 604 related individuals by average identity-by-descent allele sharing (PiHAT ≥ 0.185 in PLINK), 1 sample due to poor genotype calling, and a further 4 ethnic outliers, leaving 3,871 case subjects and 4,686 control subjects for the association analysis. Here we report on the analysis of common variants within this cohort: 238,825 SNPs with minor allele frequency ≥ 0.05, as less common variants were poorly called.

For the discovery phase, we performed association analysis using logistic regression with sex and the first two principal component (PC1 and PC2) of the PCA as covariates. We did not use further principal components in our regression model as after adjustment for PC2, we saw no further separation of distinct subsets, and the genomic inflation factor did not decrease further (λ_GC_ unadjusted = 1.104; adjusted for PC1 λ_GC_ = 1.089; adjusted for PC1 and PC2 λ_GC_ = 1.089; PC1, PC2, and PC3 λ_GC_ = 1.090). We calculated this overdispersion factor of association test statistics (λ_GC_) using observed versus expected p values, and adjusted for sample size by calculating λ_1000_^17^ (Figure S3). Conditional analysis was performed at each associated locus, again using logistic regression conditioning on the most statistically associated SNP at each locus. If a second independent signal was detected (p ≤ 5.0 × 10^−8^), we conditioned on that SNP, repeating the process until no further independent associations were evident.

We selected SNPs for replication that showed a putative association in the discovery cohort with p ≤ 1 × 10^−5^ (Table S1). The integrity of each of these associations was confirmed by manual inspection of the genotyping intensity plot (Figure S4).

For replication, additional UK case subjects and Dutch case and control subjects were genotyped at the prioritized SNPs using the Sequenom MassARRAY platform. German case and control subjects were previously genotyped on the Affymetrix Human SNP Array 6.0. Where no direct or tag SNP was available on the Affymetrix platform, German case and control subjects were genotyped using TaqMan probes (Table S1). SNPs with call rate < 90% or SNPs with deviation from HWE (p < 0.0001) were removed, leaving 46 (31 in the German cohort, 41 in the Netherlands cohort, and 42 in the UK cohort) in the final dataset. 246 samples were removed due to call rate < 90%, and 70 samples were removed due to sex mismatch between self-reported data and genotyping result. SNP rs2598107 was separately replicated only on the UK cohort, 38 samples of which were removed due to call rate < 90%. The remaining UKHLS control subjects were again genotyped on the Illumina HumanCoreExome platform and underwent QC as described above. We used multiple genotyping platforms in the replication phase, so constructed Forest plots in R to check for heterogeneity. Since our replication signals were in the same direction and of similar magnitude to our discovery results, it is unlikely that genotyping artifact was responsible for the observed associations (Figure S5).

For association analysis of the replication phase, we performed logistic regression and used sex as a covariate. The Breslow-Day test was used to test for heterogeneity. We performed a fixed-effects meta-analysis of discovery and replication phase using the inverse variance method, assuming all studies share a common true effect size at each locus. The explained heritability for Dupuytren disease was estimated using the GCTA package.^18^

For imputation, we phased our dataset using SHAPEIT2.^19^ The phased dataset was submitted to the Haplotype Reference Consortium imputation service, utilizing the Sanger server and the standard PBWT pipeline.^20^ Imputed data were subjected to quality control. We removed SNPs with info score < 0.3, MAF < 1%, or significant deviation from Hardy Weinberg equilibrium (p < 1 × 10^6^). We used SNPTEST v2.5.2^21^ to calculate the Bayes factor for each SNP with the assumption that there was only one causal SNP per associated region, and the additive model of prior distribution. For regions that contained two index SNPs, we identified the BF for the second index SNP by conditioning on the first index SNPs. Posterior probability was defined as Bayes factor for SNP^k^ divided by the summation of the BF for every SNP in the selected region, 500 kb upstream and downstream of the index SNP, as previously described.^22^ 99% credible sets were constructed by summing the ranked posterior probability of every SNP within each associated region until the total reached 0.99.

### Tissue Culture

Primary cells were disaggregated from fresh surgical tissue samples using 300 units/g type II collagenase at 1 mg/mL (Worthington Chemical) in DMEM (Lonza) supplemented with 5% FBS (Labtech) overnight at 37°C with 5% CO_2_. After incubation, cells were filtered using 40 μm tissue culture strainer, pelleted, and cultured on 10 cm^2^ Petri dishes. Primary myofibroblasts were cultured in DMEM supplemented with 10% FBS (Labtech), 1% penicillin/streptomycin, and 1× Glutamax (ThermoFisher Scientific).

### Immunocytochemistry

Diseased, surgically resected palmar fascia was disaggregated as previously described,^23^ and myofibroblasts were seeded on 35 mm FluoroDish tissue culture dishes (World Precision Instruments) at 50,000 cells per dish. Cells were fixed in 4% formaldehyde and permeabilized using 0.1% triton X. SFRP4 was stained using goat anti-SFRP4 primary IgG antibody (AF1827, R&D Systems) and rabbit anti-goat IgG Alexa Fluor 633 (A-21086, Thermofisher Scientific). Filamentous actin and nuclei were stained using Acti-stain 488 phalloidin (Cytoskeleton Inc.) and Hoechst 33342 (Thermofisher Scientific). Fluorescence images were acquired using a confocal laser scanning microscope (Zeiss LSM 710) with a 40× objective.

### Immunohistochemistry

Dupuytren fascia tissue and palm skin were processed by the Kennedy Institute of Rheumatology histopathology service unit. Briefly, samples were dehydrated in a tissue processor Tissue Tek VIP (Sakura, 60320296-1210) and paraffin embedded with Tissue-Tek TEC (Sakura 5230-1177). 5 μM sequential sections were obtained and mounted onto Surgipath X-tra Adhesive slides (Leica, Milton Keynes) or Polysine slides (ThermoFisher Scientific). Slides were baked at 60°C for 60 min and submerged in a FLEX TRS filled PT Link machine for deparaffinization and antigen retrieval. Immunostaining was performed using an Autostainier Link 48 machine with rabbit anti-SFRP4 primary antibody (Abcam cat# AB32784; RRID: AB_2187103) or rabbit anti-WNT3A primary antibody (GTX128101, GeneTex). Antigen binding was visualized using FLEX 3,3′-diaminobenzidine (DAB) substrate working solution and was counterstained with hematoxylin (Dako). Flex Rabbit isotype control (Dako) was used as a reference for non-specific antigen binding. All images were obtained using a Zeiss AXIO Imager microscope and 20× objective.

### RNA Expression

For basal expression level experiments, myofibroblast cells of defined genotype at rs16879765 were thawed from storage in liquid nitrogen, then plated on 6-well plates at a density of 1 × 10^5^ cells per well or 24-well plates at 5 × 10^4^ cells per well, without antibiotics. RNA was extracted after 24 hr of culture using Trizol (ThermoFisher Scientific) and Direct-zol RNA MiniPrep kit (Zymo Research) according to the manufacturer’s instructions. Reverse transcription was performed using High-Capacity RNA-to-cDNA kit (ThermoFisher Scientific) according to the manufacturer’s instructions. Quantitative PCR (qPCR) was performed using Taqman Advance Master Mix with pre-designed Taqman probes (Thermo Fisher Scientific) for genes of interest, and control gene *18S*. Relative quantification over control genes was calculated using the ΔCt method. The statistical significance between means was tested using a two-tailed Student’s t test, with equal variances assumed. Each experiment was conducted in triplicate on cells derived from independent individuals (CC n = 10; CT n = 9; TT n = 7). A p value of less than 0.05 was considered significant.

For stimulation experiments, primary cells derived from surgically resected DD fascia were cultured as described above. Cells were plated on 6-well plates at 1 × 10^5^ cells per well and serum starved for 24 hr. Cells were then stimulated with vehicle control or a combination of recombinant WNT3A (200 ng/mL), SFRP4 (8 μg/mL), or DKK1 (100 ng/mL). Cells were harvested at 48 hr, and relative expression of genes of interest was determined by qPCR using Taqman probes as described above. Results were calculated using the ΔΔCt method and are expressed as relative expression compared to WNT3A stimulation alone, which was normalized to *18S* as described above. Each experiment was performed on cells derived from four independent individuals. The statistical significance between means was tested using two-tailed Student’s t test with equal variance assumed, and a p value of less than 0.05 was considered significant.

### SFRP4 Protein Expression

Intracellular and extracellular protein expression of SFRP4 was determined using the sandwich ELISA kit (Phadia Gmbh) provided by Dr. Hoffmann, University of Freiburg.^24^ Intracellular protein was collected by cell scraping in 100 μL of RIPA buffer, supplemented with 1% protease inhibitor cocktail (PIC). Total protein level was determined using bicinchoninic acid assay (Millipore). We performed the ELISA according to manufacturer’s instructions. All wash steps specified below were performed using the wash buffer provided by the manufacturer unless stated otherwise. Samples were diluted 1 in 10 with the sample diluent and loaded onto SFRP4 antibody-coated 8-well strips. After 60 min of incubation at room temperature, the solution was discarded and the wells were washed three times. 100 μL of primary antibodies (Phadia Gmbh) was added to the wells, followed by 60 min incubation at room temperature. The solution was removed, and the wells were washed three times. 100 μL of conjugate was added and incubated for 30 min of incubation at room temperature. After discarding the conjugate and washing the wells three times, 50 μL of HRP substrate, 3,3′,5,5′-Tetramethylbenzidine (TMB) was added. The reaction was then terminated using the stop solution after 30 min of incubation in the dark. The absorbance of the solution was determined using photometer at 450 nm with reference wavelength at 620 nm. Absolute concentration was calculated using the standard curve generated. After an initial range-finding experiment, the time point of 7 days was selected for the full experiment. Each experiment was repeated in duplicate on cells from independent individuals (CC n = 9; TT n = 6), at 7 days from the beginning of culture. The statistical significance between means was tested using two-tailed Student’s t test, and a p value of less than 0.05 was considered significant.

## Results

### GWAS

**Figure 1:**
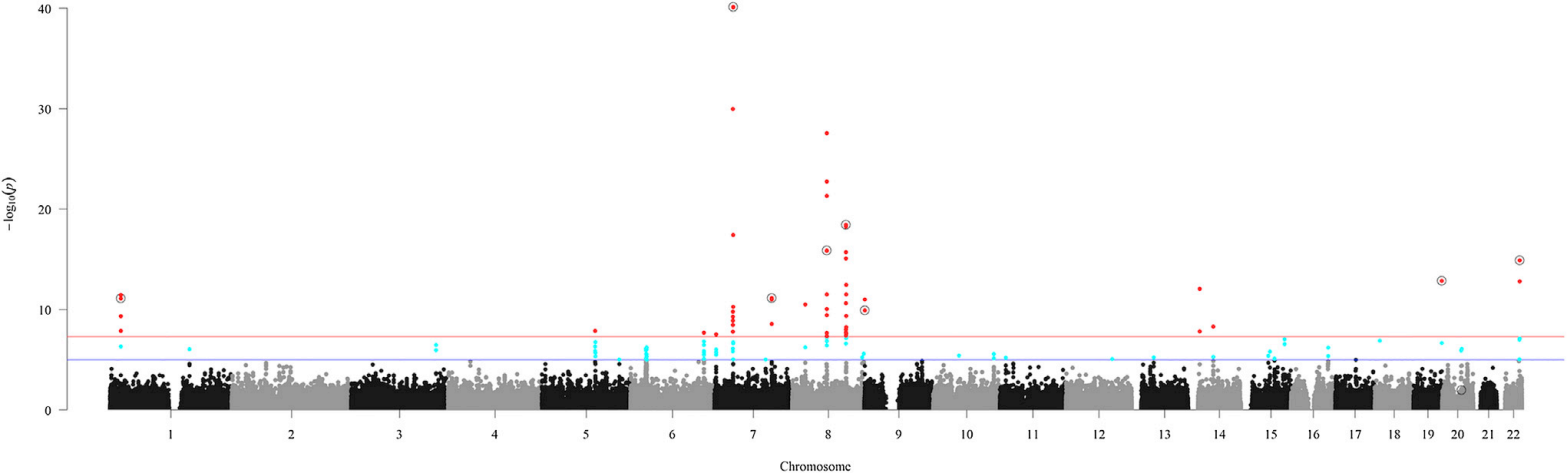
Manhattan Plot for the Discovery Association Analysis The horizontal blue line represents p = 1 × 10^−5^ and the horizontal red line indicates p = 5 × 10^−8^. Variants colored in cyan are suggestive of association (p ≤ 1 × 10^−5^) and those colored red have genome-wide significant association (p ≤ 5 × 10^−8^). The nine previously reported associated loci are indicated by an open circle surrounding the SNP.

In the GWAS discovery phase, we used the Illumina HumanCoreExome array to test 238,825 common-frequency variants (MAF ≥ 0.05) for association with DD in 3,871 UK case subjects and 4,686 UK control subjects, after quality control. This yielded genome-wide significant associations (p ≤ 5 × 10^−8^) at 14 variants, including 8 of the 9 previously reported loci (Figure 1).

In the replication phase, we genotyped 45 SNPs with p ≤ 1 × 10^−5^ in the discovery set, in a total of 4,041 case subjects and 8,251 control subjects from the UK, the Netherlands, and Germany. In addition, there were a further four SNPs with suggestive association (p ≤ 1 × 10^−5^) and one with genome-wide significant association (rs246105, frequency 20.1%, OR = 0.796, p = 1.34 × 10^−8^) for which we were unable to design an appropriate assay (Tables 1 and S1). After fixed-effects meta-analysis, we confirmed association at all 9 previously reported loci and defined 15 further loci with genome-wide significant evidence of association (Table 1; Figure S6).

**Table 1:** SNPs Significantly Associated with Dupuytren Disease (p ≤ 5 × 10^−8^)

**Chromosome**	**Position**^a^	**rsID**	**Allele**	**EAF**^b^	**Discovery**		**Replication**		**Meta-analysis**			**Selected Nearby Genes**
					**p**	**OR**	**p**	**OR**	**p**	**OR**	**(95% CI)**	
1	22698447	rs7524102	G	0.214	7.68 × 10^−12^	1.332	6.43 × 10^−5^	1.448	3.00 × 10^−15^	1.351	1.254–1.456	*WNT4*, *ZBTB40*
1	162672011	rs17433710	C	0.12	9.13 × 10^−7^	0.791	3.73 × 10^−5^	0.833	1.99 × 10^−10^	0.813	0.763–0.867	*DDR2*, *HSD17B7*
5	108672946	rs246105^c^	T	0.201	1.34 × 10^−8^	0.796	–	–	–	–	–	*PJA2*
6	149797014	rs394563	T	0.411	2.03 × 10^−8^	0.828	1.02 × 10^−10^	0.827	1.14 × 10^−17^	0.828	0.793–0.864	*ZC3H12D*, *TAB2*, *SUMO4*
7	3318658	rs10276303	T	0.26	2.89 × 10^−8^	0.817	6.00 × 10^−9^	0.831	9.63 × 10^−16^	0.825	0.787–0.865	*SDK1*, *CARD11*
7	37973014	rs2598107^d^	T	0.447	1.11 × 10^−30^	1.478	1.82 × 10^−15^	1.475	1.55 × 10^−44^	1.477	1.399–1.56	*SFRP4*, *EPDR1*
7	37989095	rs16879765	T	0.178	7.15 × 10^−41^	1.926	2.82 × 10^−42^	1.837	3.38 × 10^−81^	1.877	1.759–2.002	*SFRP4*, *EPDR1*
7	116892846	rs38904	C	0.464	1.02 × 10^−11^	1.254	6.52 × 10^−13^	1.253	4.24 × 10^−23^	1.253	1.199–1.311	*WNT2*
8	25845675	rs10866846	A	0.421	3.14 × 10^−11^	1.249	1.78 × 10^−6^	1.148	1.75 × 10^−15^	1.19	1.14–1.242	*EBF2*
8	69992380	rs2912522^d^	G	0.201	1.29 × 10^−16^	0.72	3.26 × 10^−14^	0.751	4.09 × 10^−29^	0.736	0.698–0.777	*LOC100505718*
8	70007938	rs629535	T	0.351	2.84 × 10^−28^	1.477	1.17 × 10^−15^	1.275	4.31 × 10^−40^	1.357	1.297–1.42	*LOC100505718*
8	109228008	rs611744	G	0.402	3.70 × 10^−19^	0.737	9.92 × 10^−16^	0.794	1.15 × 10^−32^	0.77	0.737–0.804	*EIF3E*, *RSPO2*
8	145504343	rs7838717	T	0.405	2.55 × 10^−6^	1.173	3.91 × 10^−9^	1.188	4.81 × 10^−14^	1.182	1.131–1.234	*BOP1*, *HSF1*, *DGAT1*
9	1201156	rs12342106	A	0.308	9.76 × 10^−12^	1.289	6.40 × 10^−16^	1.29	3.78 × 10^−26^	1.29	1.23–1.352	*LINC01230*, *DMRT1*, *DMRT2*, *DMRT3*
13	44842503	rs9525927	G	0.167	5.80 × 10^−6^	0.823	6.76 × 10^−6^	0.842	1.82 × 10^−10^	0.833	0.788–0.881	*MIR8079*, *SMIM2*, *SERP2*
14	23312594	rs1042704	A	0.248	8.72 × 10^−13^	1.326	1.12 × 10^−8^	1.213	2.49 × 10^−19^	1.259	1.198–1.324	*MMP14*
14	51074461	rs1032466	C	0.306	4.90 × 10^−9^	0.812	6.82 × 10^−10^	0.824	1.96 × 10^−17^	0.818	0.781–0.857	*ATL1*, *MAP4K5*, *SAV1*
15	56229760	rs1509406	G	0.356	4.03 × 10^−6^	1.175	1.41 × 10^−5^	1.154	2.59 × 10^−10^	1.164	1.110–1.22	*NEDD4*
15	68628163	rs2306022	T	0.11	7.59 × 10^−6^	1.286	2.57 × 10^−6^	1.266	8.70 × 10^−11^	1.275	1.185–1.372	*ITGA11*
15	89238184	rs6496519	T	0.164	9.35 × 10^−8^	0.795	1.42 × 10^−10^	0.789	7.18 × 10^−17^	0.791	0.749–0.836	*ISG20*, *ACAN*, *AEN*
16	75506593	rs977987	G	0.403	6.24 × 10^−7^	1.184	8.84 × 10^−5^	1.12	4.82 × 10^−10^	1.146	1.098–1.197	*CHST6*, *TMEM170A*, *CFDP1*
18	9762933	rs9951109	C	0.133	1.24 × 10^−7^	0.776	8.89 × 10^−5^	0.852	1.43 × 10^−10^	0.82	0.771–0.871	*RAB31*
19	57678194	rs11672517	A	0.284	1.42 × 10^−13^	1.331	2.71 × 10^−5^	1.384	1.99 × 10^−17^	1.341	1.254–1.435	*DUXA*, *ZIM3*, *ZNF264*
20	38300807	rs6016142	T	0.132	1.19 × 10^−6^	1.282	1.98 × 10^−8^	1.274	1.15 × 10^−13^	1.277	1.197–1.363	*LINC01370*, *LOC339568*
20	39320751	rs6102095	A	0.125	8.54 × 10^−7^	0.792	1.10 × 10^−13^	0.71	1.96 × 10^−18^	0.748	0.701–0.799	*MAFB*
22	46459132	rs7291412	T	0.413	1.24 × 10^−15^	1.316	1.14 × 10^−16^	1.269	1.53 × 10^−30^	1.288	1.234–1.345	*WNT7B*, *MIRLET7BHG*

a Based on human genome build hg19.

b The effect allele frequency (EAF) in the total cohort is shown, except for rs246105, where the effect allele frequency in the discovery set is shown.

c We were unable to design an assay for this SNP in the replication phase.

d Identified by conditional analysis.

Conditional analysis at all associated loci confirmed two independent signals at two loci. On chromosome 7, after conditioning on rs16879765, rs2598107 showed residual evidence of association (r^2^∼0, frequency 44.7%, OR = 1.48, p_cond_ = 6.85 × 10^−31^). Similarly, on chromosome 8, after conditioning on rs629535, rs2912522 showed residual evidence of association (r^2^∼0, frequency 19.7%, OR = 0.73, p_cond_ = 1.31 × 10^−14^; Table 1).

To further characterize the genetic architecture of DD, we tested first the contribution of all autosomal common-frequency variants (MAF ≥ 0.05) and second the 26 genome-wide significant variants alone to trait variance using genome-wide complex trait analysis (GCTA)^25^ and estimated them to be 53.1% and 11.3%, respectively.

### Imputation and Construction of 99% Credible Sets

We imputed our dataset using the Haplotype Reference Consortium resource. We calculated single SNP Bayes Factors (BF) for 7,218,238 SNPs within our imputed dataset, containing variants that passed our QC criteria. Variants with the highest BF within each associated region from our meta-analysis were used as the index SNP for the construction of 99% credible sets. Similar to our primary analysis, conditional analysis revealed the same two loci with two independent signals. On chromosome 7, after conditioning on rs117402009 (BF = 9.87 × 10^45^), we found residual evidence of association for rs2598100 (BF_cond_ = 7.29 × 10^31^), and on chromosome 8, after conditioning on rs2472141 (BF = 2.22 × 10^28^), we found residual association for rs2981040 (BF_cond_ = 1.75 × 10^11^). We therefore constructed independent credible sets based around each independent signal at these loci (Figure S7 and Table S2). Intriguingly, for one of the credible sets constructed, the genotyped SNP rs1042704 (BF = 1.67 × 10^10^) had a posterior probability greater than 0.99 and therefore appears to be the causative allele at that locus. Overall, the credible sets range in size from 1 to 293 variants, with a median size of 27.5. Details of the 99% credible sets can be found in Table S2 and Figure S7.

### rs16879765

**Figure 2:**
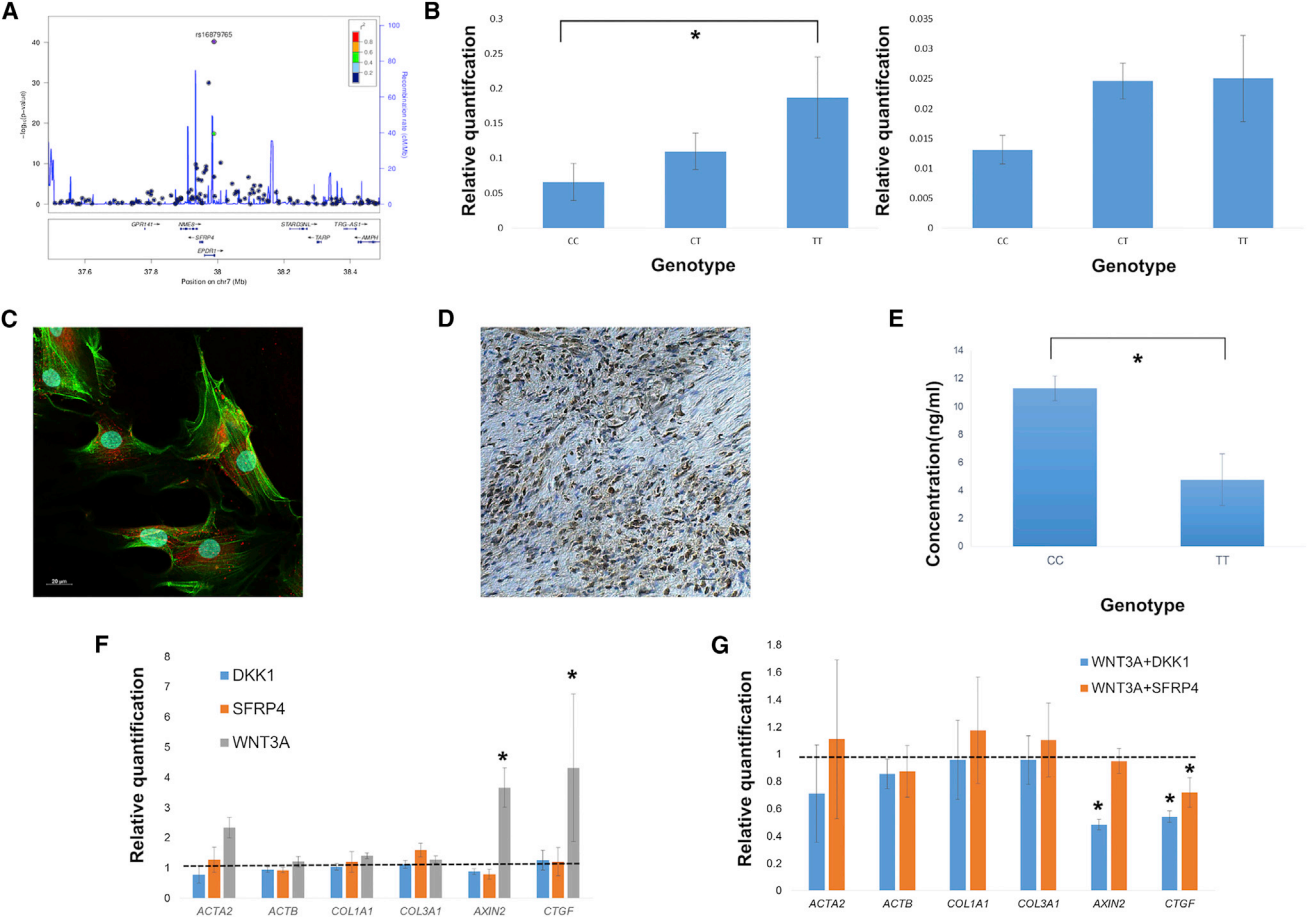
The High-Risk Genotype at rs16879765 Is Associated with a Reduction in SFRP4 Protein Secretion and Reduces Inhibition of Non-canonical WNT Signaling (A) Annotated regional association plot for the 7p14.1 locus, generated using LocusZoom software.^57^ Recombination rates were derived from HapMap data. (B) qPCR of genotyped DD-derived myofibroblasts revealed that the high-risk TT genotype was associated with increased mRNA expression of *SFRP4* (left) but not *EPDR1* (right). Each experiment was conducted in triplicate on cells derived from independent individuals (CC n = 10; CT n = 9; TT n = 7). Error bars represent the standard error of the mean. (C) Immunocytochemistry reveals robust expression of SFRP4 (red) in DD-derived myofibroblasts. Nuclei are stained blue, and F-actin is stained green. (D) Immunohistochemistry in fixed surgically resected DD fibrotic fascia confirms expression of SFRP4 (brown). (E) ELISA of supernatant from DD-derived myofibroblasts shows that decreased extracellular accumulation of SFRP4 protein is associated with the high-risk TT genotype at rs16879765. Each experiment was repeated in duplicate on cells from independent individuals (CC n = 9; TT n = 6), at 7 days from the beginning of culture. Error bars represent the standard error of the mean. (F and G) WNT3A stimulation of DD-derived myofibroblasts upregulates both the canonical (*AXIN2*) and non-canonical (*CTGF*) pathways, and also upregulates the expression of α-smooth muscle actin (*ACTA2*), while having no effect on the expression of β-actin (*ACTB*) or collagen types I (*COL1A1*) or III (*COL3A1*). The addition of SFRP4 or non-specific WNT inhibitor DKK1 alone has no appreciable effect on signaling or expression of any tested gene. The addition of SFRP4 in combination with WNT3A selectively inhibits signaling via the non-canonical pathway, whereas DKK1 inhibits both canonical and non-canonical signaling. Each experiment was repeated in duplicate on cells from independent individuals (CC n = 9; TT n = 6) at 7 days from the beginning of culture. ^∗^p < 0.05.

We further investigated the functional consequences of the statistically most associated SNP from the direct genotyping, rs16879765, as a proof of principle that using myofibroblasts from surgically resected DD tissue could help define the causative gene at a particular locus. This SNP is located in an intron of the gene *EPDR1* and approximately 4 kb upstream of *SFRP4* (MIM: 606570) (Figure 2A). EPDR1 is a poorly characterized type II transmembrane protein that shares some homology with ependymins and protocadherins. EPDR1 has been shown to be upregulated in CD34^+^ hematopoetic stem cells and colorectal cancer cells.^26^, ^27^ SFRP4 is a secreted protein with homology to the membrane-bound WNT receptors FZD. It is thought to modulate WNT signaling by competing for WNT ligands with Frizzled receptors.^28^

We utilized myofibroblasts up to passage three derived from surgically resected DD samples to study the genotype-specific expression of *SFRP4* and *EPDR1*. The homozygous high-risk (TT) genotype at rs16879765 showed significantly greater *SFRP4* expression compared to the low-risk (CC) genotype, with the heterozygous state showing intermediate expression levels. There was no genotype-specific differential expression of *EPDR1* (Figure 2B). Immunocytochemistry performed on disaggregated primary cells from surgically resected DD tissue failed to demonstrate EPDR1 expression but showed cytoplasmic SFRP4 expression (Figure 2C). Furthermore, immunohistochemistry confirmed expression of SFRP4 in fixed surgically resected fibrotic DD tissue (Figure 2D), but not in palm skin or using isotype control antibody (Figure S8). We used ELISA to examine the expression of SFRP4 protein in DD-derived myofibroblasts. There was decreased accumulation of extracellular SFRP4 from the high-risk genotype cells (Figure 2E), consistent with the role of SFRP4 as a secreted WNT antagonist. As SFRP4 has been previously shown to bind to WNT3A,^29^ we first examined WNT3A expression by immunohistochemistry in our fixed surgical specimens and showed expression in surgically resected fibrotic DD tissue, but not in palm skin or using isotype control antibody (Figure S8). We then used recombinant human WNT3A, SFRP4, or a combination of the two proteins to stimulate DD-tissue-derived myofibroblasts, palmar-skin-derived fibroblasts, and non-palmar-skin-derived fibroblasts from four unrelated DD-affected individuals. We found no difference in expression of collagen type I or type III in any of the cells tested. WNT3A selectively increased the expression of α-SMA in the Dupuytren myofibroblasts. Furthermore, WNT3A increased signaling in both canonical and non-canonical WNT signaling pathways, as evidenced by increased *AXIN2* (MIM: 604025) and *CTGF* (MIM: 121009) expression, respectively^30^ (Figure 2F). Interestingly, SFRP4 appeared to act as a selective antagonist of non-canonical signaling by WNT3A but had no significant effect on canonical signaling (Figure 2G).

## Discussion

We have completed the largest GWAS to date in DD, the most common inherited disorder of connective tissue. Our results have almost tripled the known loci associated with this localized fibrosis and have also highlighted the role of fundamental biological processes in the pathophysiology of fibrosis, in the context of DD. Several associated loci harbor potentially attractive drug targets and are the subject of active further research. While we acknowledge that the mechanistic link between associated SNPs and pathophysiological function can often be obscure and requires experimental validation, we think that certain biological processes deserve discussion.

### WNT Signaling

The importance of WNT signaling in fibrosis, as exemplified by DD, has been confirmed by this work. All previously reported loci that harbor WNT pathway genes have been replicated in this larger study, including WNT ligands WNT2, WNT4, and WNT7B, a co-signaling molecule RSPO2, and WNT antagonist SFRP4.^15^

Our detailed functional studies on the statistically most strongly associated variant (rs16879765) have suggested that a subtle imbalance of WNT signaling contributes to the fibrotic phenotype. We postulate that the decreased SFRP4 secretion seen in individuals homozygous for the high-risk allele at rs16879765 allows a subtle increase in WNT3A signaling through the non-canonical pathway. This could lead to greater α-SMA expression and hence contraction of the DD cords.^31^ This contraction is characteristic of the latter stages of DD and requires surgical treatment.

Taken as a whole, our genetic results suggest that subtle variations in the level of WNT signaling are likely to be responsible for the fibrosis seen in DD. The genetic variants cluster around ligands, a co-stimulatory molecule, and a WNT antagonist. This contrasts with variants in the WNT signaling pathway that predispose to cancer, which tend to be downstream of the receptor and lead to receptor-independent signaling and unrestrained cellular growth and proliferation.^32^ While DD does share some clinical features with cancer, such as excess cellular proliferation, abnormal extracellular matrix deposition,^33^ and the tendency to recur after treatment, it is ultimately a benign phenotype.

### Extracellular Matrix Modulation

Fibrotic disease is characterized by abnormal and excessive extracellular matrix (ECM) deposition.^1^ In DD, the abnormal fibrotic cords are composed mainly of collagen, with a higher type III to type I collagen ratio than in unaffected palmar fascia.^2^ Several of our additional associated loci harbor genes that are known to interact with and modulate the ECM: *DDR2* (MIM: 191311) at chromosome 1q23.3 (rs17433710, OR = 0.81, p_meta_ = 1.99 × 10^−10^), *MMP14* (MIM: 600754) at chromosome 14q11.2 (rs1042704, OR = 1.26, p_meta_ = 2.49 × 10^−19^), *ITGA11* (MIM: 604789) at chromosome 15q23 (rs2306022, OR = 1.28, p_meta_ = 8.70 × 10^−11^), *ACAN* (MIM: 155760) at chromosome 15q26.1 (rs6496519, OR = 0.79, p_meta_ = 7.18 × 10^−17^), and *CHST6* (MIM: 605294) at chromosome 16q22 (rs977987, OR = 1.15, p_meta_ = 4.82 × 10^−10^).

Discoidin domain receptor 2 (DDR2) is a membrane-bound receptor tyrosine kinase that contains an extracellular discoidin homology domain.^34^ The functional ligand for DDR2 is fibrillar collagen (types I–III), though it has also been shown to bind type X collagen.^35^, ^36^, ^37^ DDR2 has previously been shown to play a role in collagen production and migration through the basement membrane by skin fibroblasts.^38^ Furthermore, DDR2 plays a complex role in liver fibrosis. DDR2 expression is induced by acute liver injury in a mouse model, and expression of a constitutionally active form of DDR2 enhances proliferation and invasion of hepatic stellate cells.^39^ However, in contrast to the acute injury model, DDR2 knockout mice are more susceptible to chronic inflammation and fibrosis in a carbon tetrachloride model of chronic liver injury.^40^ Intriguingly, this increased susceptibility to chronic fibrosis is mediated in part by attenuating the interaction of hepatic stellate cells with macrophages, suggesting a link between DDR2 and pro-inflammatory pathways (see below).

DDR2 expression has also been shown to be increased both in mouse models of osteoarthritis (OA) and in human OA.^41^ In this context, the effect of DDR2 is mediated by its induction of matrix metalloproteinase 13 (MMP13), the major MMP responsible for type II collagen degradation in OA.^42^ Decreased expression of DDR2 in heterozygous knockout mice lead to the attenuation of OA after joint destabilization.^43^ This raises the possibility of cross talk between DDR2 and MMP pathways that may be relevant in DD pathogenesis. DDR2 represents an attractive therapeutic target in DD and other fibrotic diseases and is currently under active investigation by several pharmaceutical companies.^44^

Matrix metalloproteinase 14 (MMP14 or MT1-MMP) is a type 1 transmembrane protein and member of the MMP family of proteases, initially characterized for their ability to degrade the extracellular matrix. MMP14 was the first membrane-bound MMP to be discovered and was initially characterized as a pro-MMP2 activator, though now at least 42 substrates have been defined, including fibrillar collagen and the WNT antagonist DKK1.^45^ There is some evidence for the involvement of MMP14 in DD pathogenesis. In clinical trials, broad-spectrum MMP inhibition caused some individuals to develop DD.^46^ MMP14 is overexpressed in DD nodules,^47^ and knockdown of MMP14 in DD-derived cells reduced both contraction and MMP2 activation *in vitro*.^48^ Interestingly, knockdown of MMPs including MMP14 did not change the rate of collagen breakdown, suggesting that non-proteolytic effects of MMP14 are responsible for the pro-fibrotic phenotype. Further characterization of the mechanism of action of MMP14 in DD may lead to the validation of this protein as a therapeutic target in fibrosis.

*ITGA11* encodes integrin alpha11, a member of the integrin family of cell-surface-adhesion receptors. These type I transmembrane proteins act as heterodimers composed of an α and β subunit, and through binding to the ECM transmit both mechanical and chemical signals to the cell.^49^ Heterodimers consisting of integrin α11β1 are fibroblast-specific collagen receptors, which are mechanically induced, and regulate myofibroblast differentiation. Furthermore, *ITGA11* has recently been shown to be overexpressed in lung samples from individuals with idiopathic pulmonary fibrosis.^50^ Also, a variant near *PTK2* (encoding focal adhesion kinase [FAK] [MIM: 600758]) at chromosome 8q24.3 showed suggestive evidence of association in our discovery cohort (rs12677559, OR = 0.86, p = 5.75 × 10^−6^; Table S3; Figure S9A), but we were unable to design a suitable assay within the replication set. FAK is a non-receptor tyrosine kinase that is an integral part of focal adhesion structure^51^ and is phosphorylated in response to integrin engagement.^52^ In a mouse model of hypertrophic scarring, another example of a localized fibrosis, fibroblast-specific FAK knockout attenuated both fibrosis and inflammation (see below), emphasizing the importance of this signaling pathway in fibrosis.^53^

### Inflammation

Chronic inflammation has long been recognized as a key player in the pathogenesis of fibrosis in multiple organs, including liver, kidney, lung, and heart.^54^ Recent work has highlighted the important role of inflammation in DD. In particular, tumor necrosis factor (TNF)—signaling via the WNT pathway—was demonstrated to selectively upregulate α-SMA and subsequent contractility in palmar-skin-derived fibroblasts from DD-affected individuals compared to unaffected control subjects.^23^ While our associated regions do not harbor many inflammatory genes, they do indicate how this cross-talk between TNF and WNT signaling might occur. *MAP4K5* (MIM: 604923)—also known as germinal center kinase-related (GCKR)—at chromosome 14q22.1 (rs1032466, OR = 0.82, p_meta_ = 1.96 × 10^−17^) has been shown to be activated by both TNF and WNT3A, and decreased expression of MAP4K5 inhibits GSK3β phosphorylation and subsequent β-catenin accumulation in B lymphocytes.^55^, ^56^ This suggests a key role for MAP4K5, integrating TNF and WNT signaling in DD fibrosis.

In conclusion, we have described the largest-scale GWAS to date in DD, a common disease that is a model human fibrotic condition. We discovered 17 additional variants predisposing to fibrosis, bringing the total described to 26. Analysis of heritability explained by these 26 variants compared to all common autosomal variants in our study suggests that there are many more common variants affecting predisposition to DD and that larger studies with greater power will detect further associated loci. We characterized the subtle nature of the genetic predisposition at our statistically most associated locus, thereby identifying a potential therapeutic target. In addition, our results have highlighted other specific biological pathways that are likely to play an important role in the pathogenesis of DD and in fibrosis more widely.

DD represents a human disease that is attractive for early-phase trials of experimental therapeutics, owing to the ready availability of affected individuals, ease of access to affected tissues, and the excision of fibrotic tissue as routine part of clinical care.

## Accession Numbers

The datasets generated and analyzed during the current study are available in the European Genome-phenome Archive repository (EGA: EGAS00001001206 for BSSH-GODD, EGA: phs000303.v1.p1 for KORA, EGA: EGAS00000000043 for GODDAF). Information on how to access the UKHLS data can be found on the Understanding Society website (see Web Resources).

## Web Resources

1000 Genomes, http://www.internationalgenome.org/
European Genome-phenome Archive (EGA), https://www.ebi.ac.uk/ega
Genotyping Chips Strand and Build Files, http://www.well.ox.ac.uk/∼wrayner/strand/
OMIM, http://www.omim.org/
PLINK 1.9, https://www.cog-genomics.org/plink2/
R statistical software, http://www.r-project.org/
Understanding Society, https://www.understandingsociety.ac.uk/

## Supplemental Data

Document S1. Figures S1–S9 and Table S1

Table S2. 99% Credible Sets for Associated Loci

Table S3. Variants Carried through to Replication Phase, if an Assay Could Be Designed Variants that reached genome-wide significance (p > 5x10-8) on meta-analysis of discovery and replication cohorts are also reported in Table 1 in the main manuscript.

Document S2. Article plus Supplemental Data

## References

[1] Friedman S.L., Sheppard D., Duffield J.S., Violette S. (2013). Therapy for fibrotic diseases: nearing the starting line. Sci. Transl. Med..

[2] Lam W.L., Rawlins J.M., Karoo R.O., Naylor I., Sharpe D.T. (2010). Re-visiting Luck’s classification: a histological analysis of Dupuytren’s disease. J. Hand Surg. Eur. Vol..

[3] Townley W.A., Baker R., Sheppard N., Grobbelaar A.O. (2006). Dupuytren’s contracture unfolded. BMJ.

[4] Wilburn J., McKenna S.P., Perry-Hinsley D., Bayat A. (2013). The impact of Dupuytren disease on patient activity and quality of life. J. Hand Surg. Am..

[5] Nanchahal J., Ball C., Swettenham J., Dutton S., Barber V., Black J., Copsey B., Dritsaki M., Taylor P., Gray A. (2017). Study protocol: A multi-centre, double blind, randomised, placebo-controlled, parallel group, phase II trial (RIDD) to determine the efficacy of intra-nodular injection of anti-TNF to control disease progression in early Dupuytren’s disease, with an embedded dose response study. Wellcome Open Res.

[6] Bebbington E., Furniss D. (2015). Linear regression analysis of Hospital Episode Statistics predicts a large increase in demand for elective hand surgery in England. J. Plast. Reconstr. Aesthet. Surg..

[7] Hindocha S., McGrouther D.A., Bayat A. (2009). Epidemiological evaluation of Dupuytren’s disease incidence and prevalence rates in relation to etiology. Hand (NY).

[8] Lanting R., van den Heuvel E.R., Westerink B., Werker P.M. (2013). Prevalence of Dupuytren disease in the Netherlands. Plast. Reconstr. Surg..

[9] Hurst L.C., Badalamente M.A., Hentz V.R., Hotchkiss R.N., Kaplan F.T., Meals R.A., Smith T.M., Rodzvilla J., CORD I Study Group (2009). Injectable collagenase clostridium histolyticum for Dupuytren’s contracture. N. Engl. J. Med..

[10] van Rijssen A.L., ter Linden H., Werker P.M. (2012). Five-year results of a randomized clinical trial on treatment in Dupuytren’s disease: percutaneous needle fasciotomy versus limited fasciectomy. Plast. Reconstr. Surg..

[11] Larsen S., Krogsgaard D.G., Aagaard Larsen L., Iachina M., Skytthe A., Frederiksen H. (2015). Genetic and environmental influences in Dupuytren’s disease: a study of 30,330 Danish twin pairs. J. Hand Surg. Eur. Vol..

[12] Capstick R., Bragg T., Giele H., Furniss D. (2013). Sibling recurrence risk in Dupuytren’s disease. J. Hand Surg. Eur. Vol..

[13] Becker K., Tinschert S., Lienert A., Bleuler P.E., Staub F., Meinel A., Rößler J., Wach W., Hoffmann R., Kühnel F. (2015). The importance of genetic susceptibility in Dupuytren’s disease. Clin. Genet..

[14] Hart M.G., Hooper G. (2005). Clinical associations of Dupuytren’s disease. Postgrad. Med. J..

[15] Dolmans G.H., Werker P.M., Hennies H.C., Furniss D., Festen E.A., Franke L., Becker K., van der Vlies P., Wolffenbuttel B.H., Tinschert S., Dutch Dupuytren Study Group, German Dupuytren Study Group, LifeLines Cohort Study, BSSH-GODD Consortium (2011). Wnt signaling and Dupuytren’s disease. N. Engl. J. Med..

[16] Stolk R.P., Rosmalen J.G., Postma D.S., de Boer R.A., Navis G., Slaets J.P., Ormel J., Wolffenbuttel B.H. (2008). Universal risk factors for multifactorial diseases: LifeLines: a three-generation population-based study. Eur. J. Epidemiol..

[17] de Bakker P.I., Ferreira M.A., Jia X., Neale B.M., Raychaudhuri S., Voight B.F. (2008). Practical aspects of imputation-driven meta-analysis of genome-wide association studies. Hum. Mol. Genet..

[18] Yang J., Lee S.H., Goddard M.E., Visscher P.M. (2011). GCTA: a tool for genome-wide complex trait analysis. Am. J. Hum. Genet..

[19] Delaneau O., Howie B., Cox A.J., Zagury J.F., Marchini J. (2013). Haplotype estimation using sequencing reads. Am. J. Hum. Genet..

[20] McCarthy S., Das S., Kretzschmar W., Delaneau O., Wood A.R., Teumer A., Kang H.M., Fuchsberger C., Danecek P., Sharp K., Haplotype Reference Consortium (2016). A reference panel of 64,976 haplotypes for genotype imputation. Nat. Genet..

[21] Marchini J., Howie B., Myers S., McVean G., Donnelly P. (2007). A new multipoint method for genome-wide association studies by imputation of genotypes. Nat. Genet..

[22] Maller J.B., McVean G., Byrnes J., Vukcevic D., Palin K., Su Z., Howson J.M., Auton A., Myers S., Morris A., Wellcome Trust Case Control Consortium (2012). Bayesian refinement of association signals for 14 loci in 3 common diseases. Nat. Genet..

[23] Verjee L.S., Verhoekx J.S., Chan J.K., Krausgruber T., Nicolaidou V., Izadi D., Davidson D., Feldmann M., Midwood K.S., Nanchahal J. (2013). Unraveling the signaling pathways promoting fibrosis in Dupuytren’s disease reveals TNF as a therapeutic target. Proc. Natl. Acad. Sci. USA.

[24] Hoffmann M.M., Werner C., Böhm M., Laufs U., Winkler K. (2014). Association of secreted frizzled-related protein 4 (SFRP4) with type 2 diabetes in patients with stable coronary artery disease. Cardiovasc. Diabetol..

[25] Lee S.H., Wray N.R., Goddard M.E., Visscher P.M. (2011). Estimating missing heritability for disease from genome-wide association studies. Am. J. Hum. Genet..

[26] Gregorio-King C.C., McLeod J.L., Collier F.M., Collier G.R., Bolton K.A., Van Der Meer G.J., Apostolopoulos J., Kirkland M.A. (2002). MERP1: a mammalian ependymin-related protein gene differentially expressed in hematopoietic cells. Gene.

[27] Nimmrich I., Erdmann S., Melchers U., Chtarbova S., Finke U., Hentsch S., Hoffmann I., Oertel M., Hoffmann W., Müller O. (2001). The novel ependymin related gene UCC1 is highly expressed in colorectal tumor cells. Cancer Lett..

[28] Cruciat C.M., Niehrs C. (2013). Secreted and transmembrane wnt inhibitors and activators. Cold Spring Harb. Perspect. Biol..

[29] Wawrzak D., Métioui M., Willems E., Hendrickx M., de Genst E., Leyns L. (2007). Wnt3a binds to several sFRPs in the nanomolar range. Biochem. Biophys. Res. Commun..

[30] Park H.W., Kim Y.C., Yu B., Moroishi T., Mo J.S., Plouffe S.W., Meng Z., Lin K.C., Yu F.X., Alexander C.M. (2015). Alternative Wnt signaling activates YAP/TAZ. Cell.

[31] Zhang C., Meng X., Zhu Z., Liu J., Deng A. (2004). Connective tissue growth factor regulates the key events in tubular epithelial to myofibroblast transition in vitro. Cell Biol. Int..

[32] cer Genome Atlas Network (2012). Comprehensive molecular characterization of human colon and rectal cancer. Nature.

[33] Lu P., Weaver V.M., Werb Z. (2012). The extracellular matrix: a dynamic niche in cancer progression. J. Cell Biol..

[34] Leitinger B. (2014). Discoidin domain receptor functions in physiological and pathological conditions. Int. Rev. Cell Mol. Biol..

[35] Shrivastava A., Radziejewski C., Campbell E., Kovac L., McGlynn M., Ryan T.E., Davis S., Goldfarb M.P., Glass D.J., Lemke G., Yancopoulos G.D. (1997). An orphan receptor tyrosine kinase family whose members serve as nonintegrin collagen receptors. Mol. Cell.

[36] Vogel W., Gish G.D., Alves F., Pawson T. (1997). The discoidin domain receptor tyrosine kinases are activated by collagen. Mol. Cell.

[37] Leitinger B., Kwan A.P. (2006). The discoidin domain receptor DDR2 is a receptor for type X collagen. Matrix Biol..

[38] Olaso E., Labrador J.P., Wang L., Ikeda K., Eng F.J., Klein R., Lovett D.H., Lin H.C., Friedman S.L. (2002). Discoidin domain receptor 2 regulates fibroblast proliferation and migration through the extracellular matrix in association with transcriptional activation of matrix metalloproteinase-2. J. Biol. Chem..

[39] Olaso E., Ikeda K., Eng F.J., Xu L., Wang L.H., Lin H.C., Friedman S.L. (2001). DDR2 receptor promotes MMP-2-mediated proliferation and invasion by hepatic stellate cells. J. Clin. Invest..

[40] Olaso E., Arteta B., Benedicto A., Crende O., Friedman S.L. (2011). Loss of discoidin domain receptor 2 promotes hepatic fibrosis after chronic carbon tetrachloride through altered paracrine interactions between hepatic stellate cells and liver-associated macrophages. Am. J. Pathol..

[41] Xu L., Peng H., Glasson S., Lee P.L., Hu K., Ijiri K., Olsen B.R., Goldring M.B., Li Y. (2007). Increased expression of the collagen receptor discoidin domain receptor 2 in articular cartilage as a key event in the pathogenesis of osteoarthritis. Arthritis Rheum..

[42] Xu L., Peng H., Wu D., Hu K., Goldring M.B., Olsen B.R., Li Y. (2005). Activation of the discoidin domain receptor 2 induces expression of matrix metalloproteinase 13 associated with osteoarthritis in mice. J. Biol. Chem..

[43] Xu L., Servais J., Polur I., Kim D., Lee P.L., Chung K., Li Y. (2010). Attenuation of osteoarthritis progression by reduction of discoidin domain receptor 2 in mice. Arthritis Rheum..

[44] Li Y., Lu X., Ren X., Ding K. (2015). Small molecule discoidin domain receptor kinase inhibitors and potential medical applications. J. Med. Chem..

[45] Itoh Y. (2015). Membrane-type matrix metalloproteinases: Their functions and regulations. Matrix Biol..

[46] Hutchinson J.W., Tierney G.M., Parsons S.L., Davis T.R. (1998). Dupuytren’s disease and frozen shoulder induced by treatment with a matrix metalloproteinase inhibitor. J. Bone Joint Surg. Br..

[47] Johnston P., Chojnowski A.J., Davidson R.K., Riley G.P., Donell S.T., Clark I.M. (2007). A complete expression profile of matrix-degrading metalloproteinases in Dupuytren’s disease. J. Hand Surg. Am..

[48] Wilkinson J.M., Davidson R.K., Swingler T.E., Jones E.R., Corps A.N., Johnston P., Riley G.P., Chojnowski A.J., Clark I.M. (2012). MMP-14 and MMP-2 are key metalloproteases in Dupuytren’s disease fibroblast-mediated contraction. Biochim. Biophys. Acta.

[49] Harburger D.S., Calderwood D.A. (2009). Integrin signalling at a glance. J. Cell Sci..

[50] DePianto D.J., Chandriani S., Abbas A.R., Jia G., N’Diaye E.N., Caplazi P., Kauder S.E., Biswas S., Karnik S.K., Ha C. (2015). Heterogeneous gene expression signatures correspond to distinct lung pathologies and biomarkers of disease severity in idiopathic pulmonary fibrosis. Thorax.

[51] Kanchanawong P., Shtengel G., Pasapera A.M., Ramko E.B., Davidson M.W., Hess H.F., Waterman C.M. (2010). Nanoscale architecture of integrin-based cell adhesions. Nature.

[52] Parsons J.T. (2003). Focal adhesion kinase: the first ten years. J. Cell Sci..

[53] Wong V.W., Rustad K.C., Akaishi S., Sorkin M., Glotzbach J.P., Januszyk M., Nelson E.R., Levi K., Paterno J., Vial I.N. (2011). Focal adhesion kinase links mechanical force to skin fibrosis via inflammatory signaling. Nat. Med..

[54] Wynn T.A., Ramalingam T.R. (2012). Mechanisms of fibrosis: therapeutic translation for fibrotic disease. Nat. Med..

[55] Shi C.S., Kehrl J.H. (2003). Tumor necrosis factor (TNF)-induced germinal center kinase-related (GCKR) and stress-activated protein kinase (SAPK) activation depends upon the E2/E3 complex Ubc13-Uev1A/TNF receptor-associated factor 2 (TRAF2). J. Biol. Chem..

[56] Shi C.S., Huang N.N., Harrison K., Han S.B., Kehrl J.H. (2006). The mitogen-activated protein kinase kinase kinase kinase GCKR positively regulates canonical and noncanonical Wnt signaling in B lymphocytes. Mol. Cell. Biol..

[57] Pruim R.J., Welch R.P., Sanna S., Teslovich T.M., Chines P.S., Gliedt T.P., Boehnke M., Abecasis G.R., Willer C.J. (2010). LocusZoom: regional visualization of genome-wide association scan results. Bioinformatics.

