# Supplementary material for: A Genome-wide Association Study of Dupuytren Disease Reveals 17 Additional Variants Implicated in Fibrosis
Source: Am J Hum Genet. 2017 Sep 7;101(3):417–27. doi: 10.1016/j.ajhg.2017.08.006 (PMC5591021; doi:10.1016/j.ajhg.2017.08.006)
Supplement: Document S1. Figures S1–S9 and Table S1 [file mmc1.pdf]

The American Journal of Human Genetics, Volume *101*

Supplemental Data

A Genome-wide Association Study of Dupuytren Disease Reveals 17 Additional Variants Implicated in Fibrosis

Michael Ng, Dipti Thakkar, Lorraine Southam, Paul Werker, Roel Ophoff, Kerstin Becker, Michael Nothnagel, Andre Franke, Peter Nürnberg, Ana Isabel Espirito-Santo, David Izadi, Hans Christian Hennies, Jagdeep Nanchahal, Eleftheria Zeggini, and Dominic Furniss

Figure S1. Quality control summary flowchart.

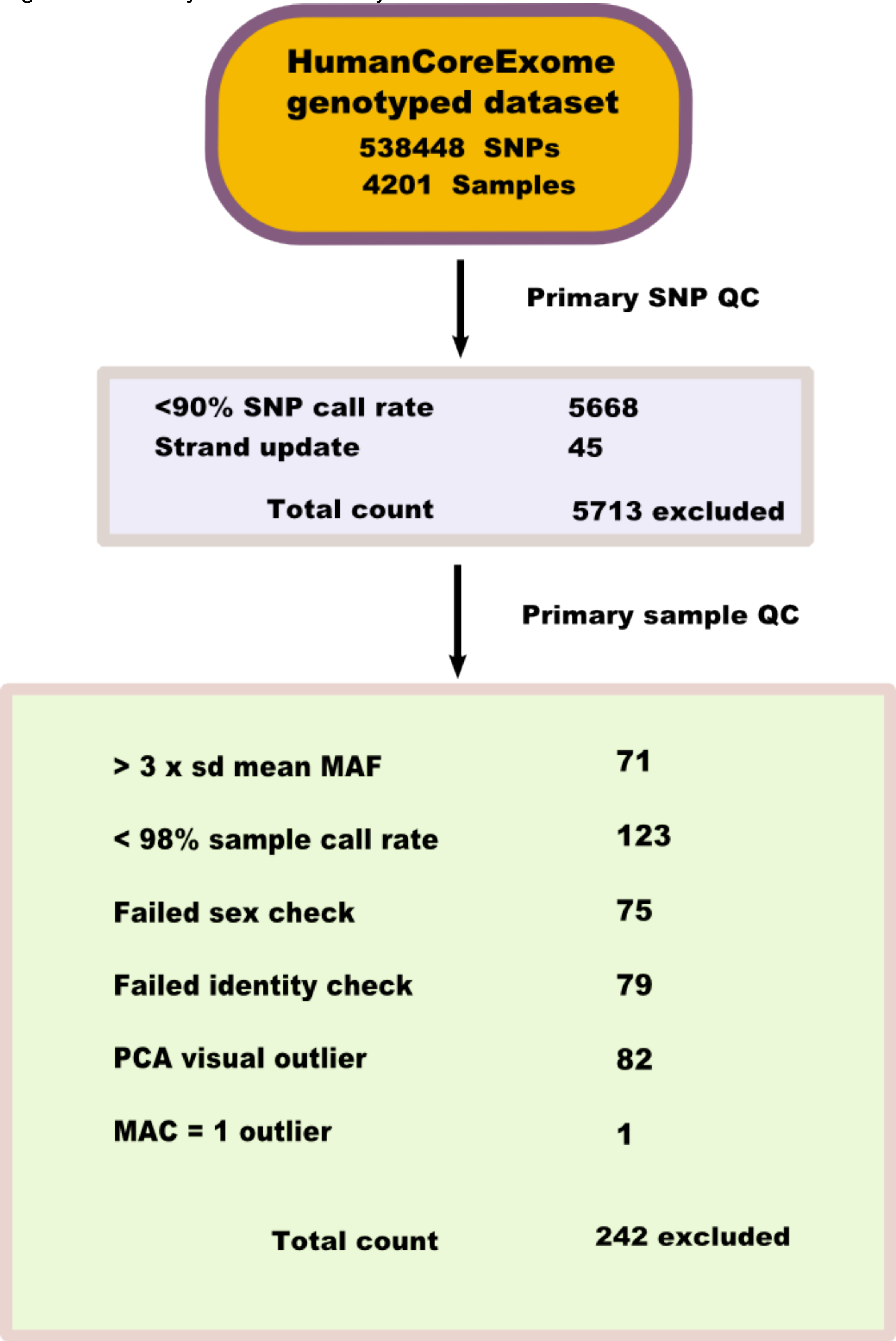

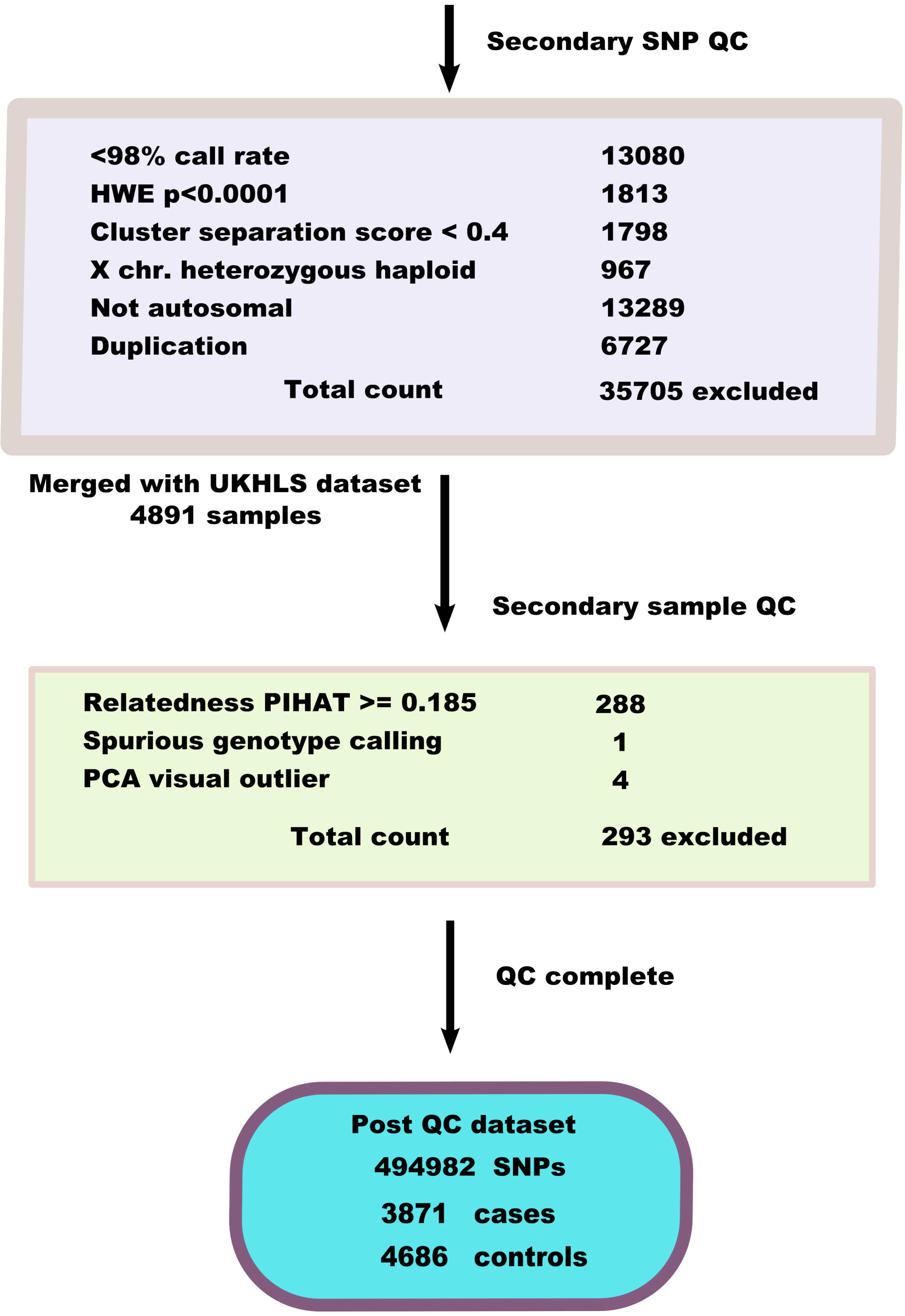
Secondary SNP QC
<98% call rate 13080
HWE p<0.0001 1813
Cluster separation score < 0.4 1798
X chr. heterozygous haploid 967
Not autosomal 13289
Duplication 6727
Total count 35705 excluded
Merged with UKHLS dataset
4891 samples
Secondary sample QC
Relatedness PIHAT >= 0.185 288
Spurious genotype calling 1
PCA visual outlier 4
Total count 293 excluded
QC complete
Post QC dataset
494982 SNPs
3871 cases
4686 controls

Figure S2. Principal component analysis plots. a) Principle components 1 and 2 plotted for cases and controls and the 1000 genomes project samples. b) Close-up view of the area highlighted in (a) demonstrating overlap of BSSH-GODD (case) and UKHLS (control) samples. Three-letter abbreviations are those used in the 1000 Genomes Project. GODD – cases from the BSSH-GODD discovery cohort. UKHLS – Controls from the UKHLS cohort.

a)

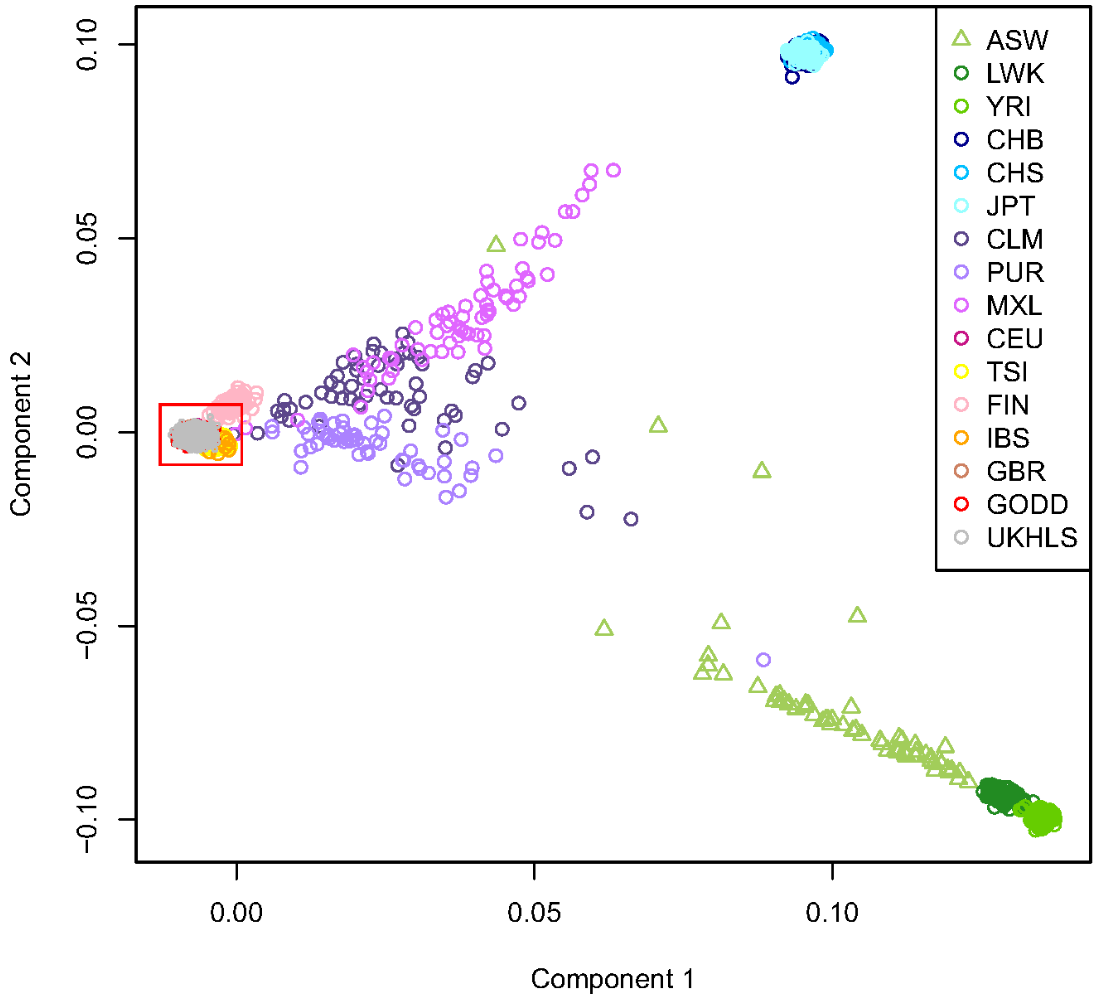

b)

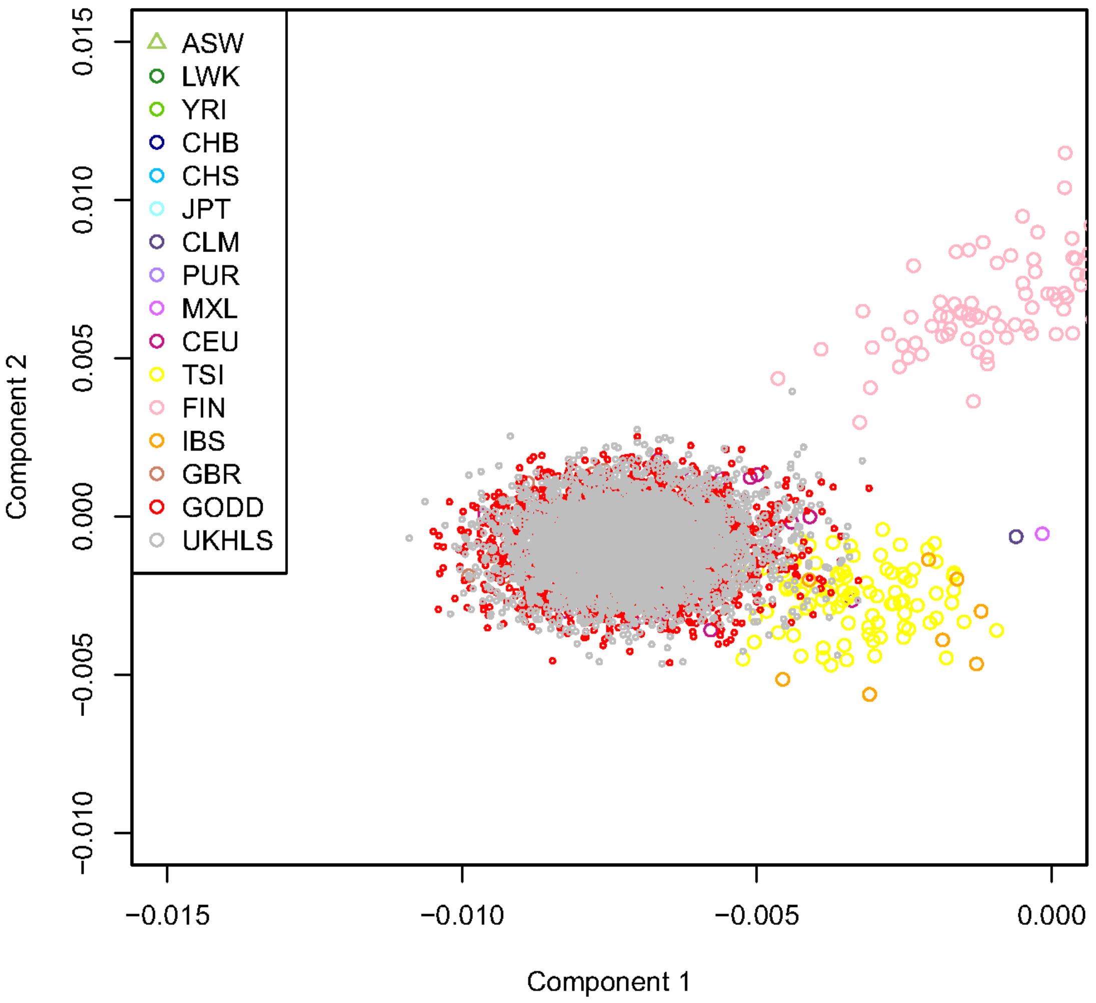
ASW
LWK
YRI
CHB
CHS
JPT
CLM
PUR
MXL
CEU
TSI
FIN
IBS
GBR
GODD
UKHLS
Component 2
0.015
0.010
0.005
0.000
−0.005
−0.010
−0.015
−0.010
−0.005
0.000
Component 1

Figure S3. Q-Q plot. Observed versus expected p-values of association in the discovery cohort. The unadjusted genomic control inflation factor (λ_{GC}) was 1.104. The genomic control inflation factor adjusted to a case control sample size of 1000 (λ_{1000}) [2] was 1.025

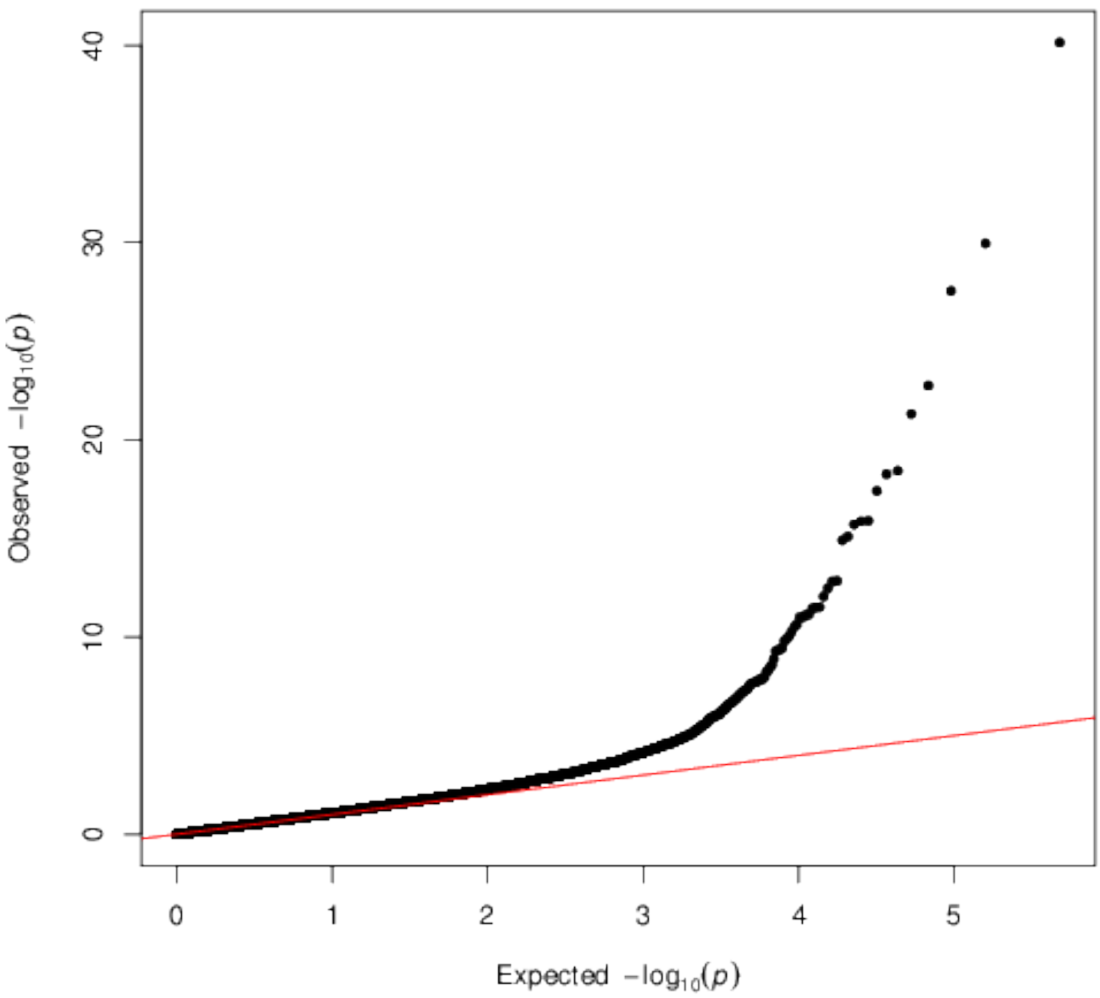

Figure S4. Raw intensity scatter plots of all genome wide significant associated variants generated using R. 00 denotes missing genotype.

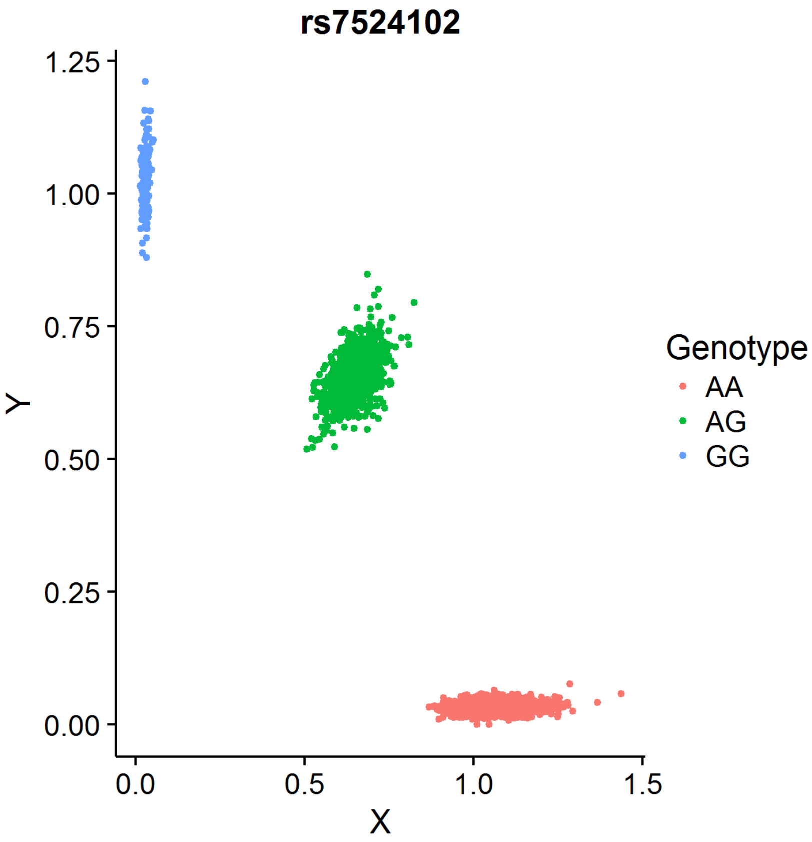

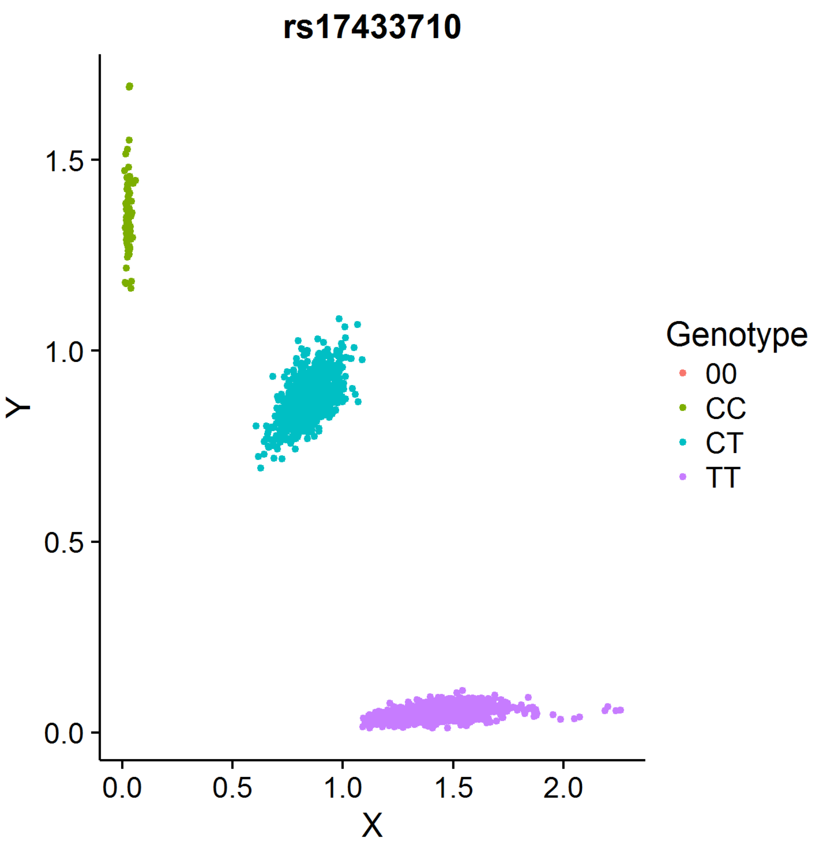
rs17433710
Y
1.5
1.0
0.5
0.0
0.0
0.5
1.0
1.5
2.0
X
Genotype
00
CC
CT
TT

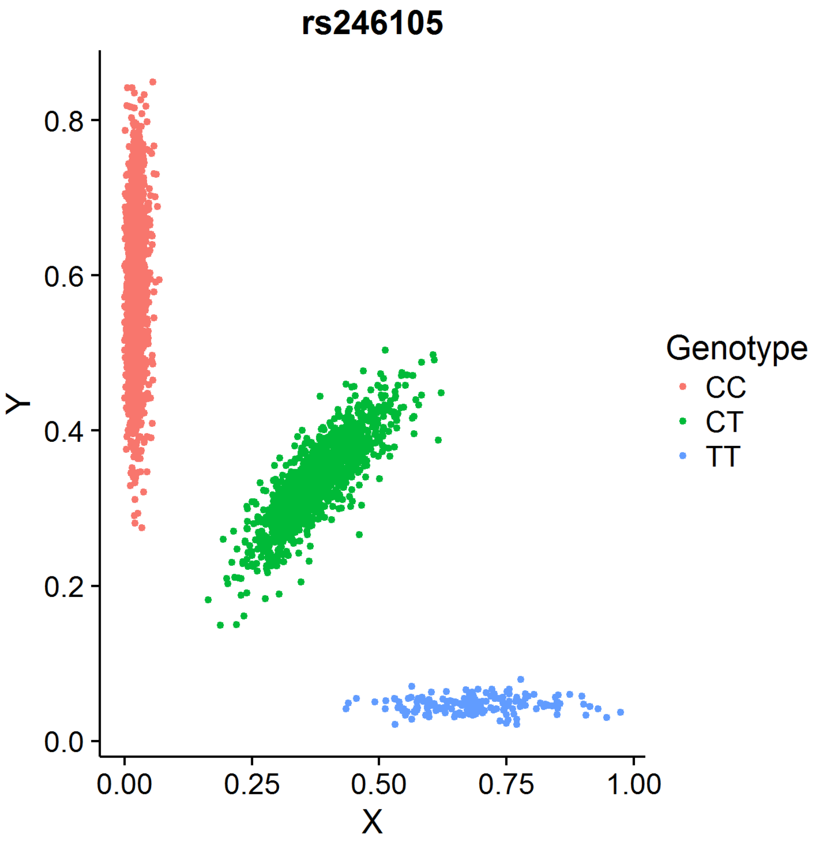
rs246105
Y
0.8
0.6
0.4
0.2
0.0
0.00
0.25
0.50
0.75
1.00
X
Genotype
CC
CT
TT

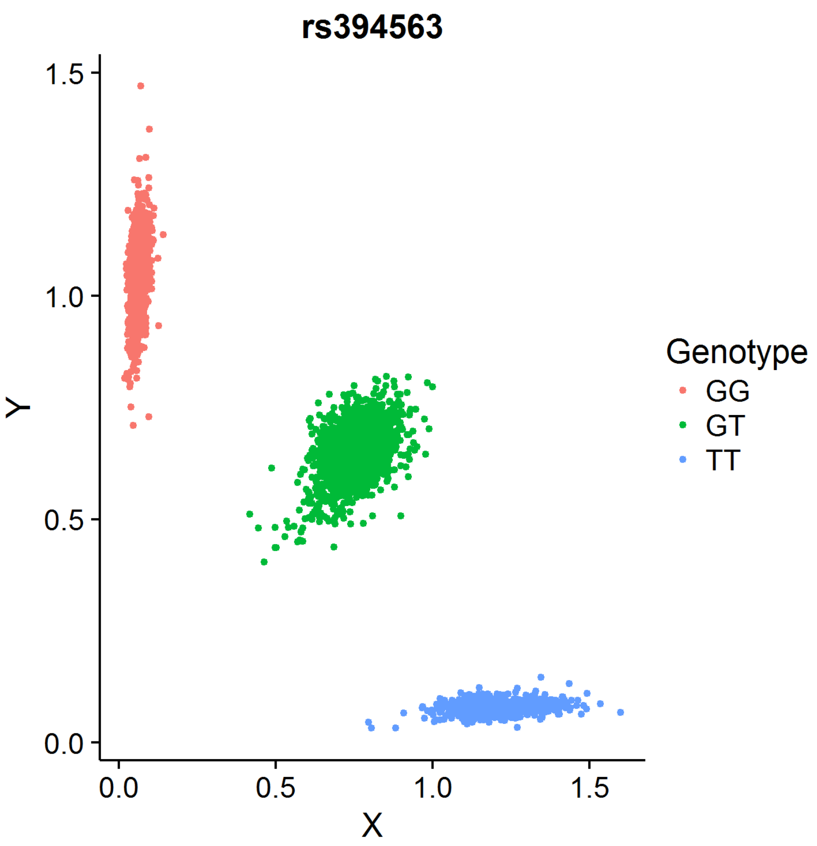
rs394563
1.5
1.0
0.5
0.0
Y
0.0
0.5
1.0
1.5
X
Genotype
GG
GT
TT

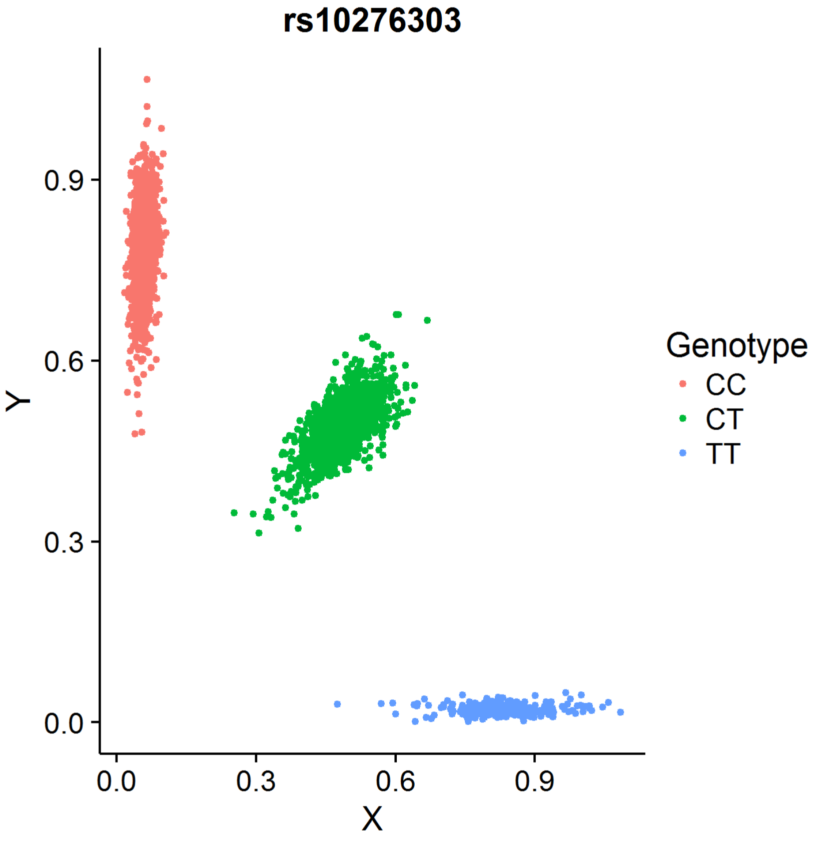
rs10276303
0.9
0.6
0.3
0.0
Y
0.0
0.3
0.6
0.9
X
Genotype
CC
CT
TT

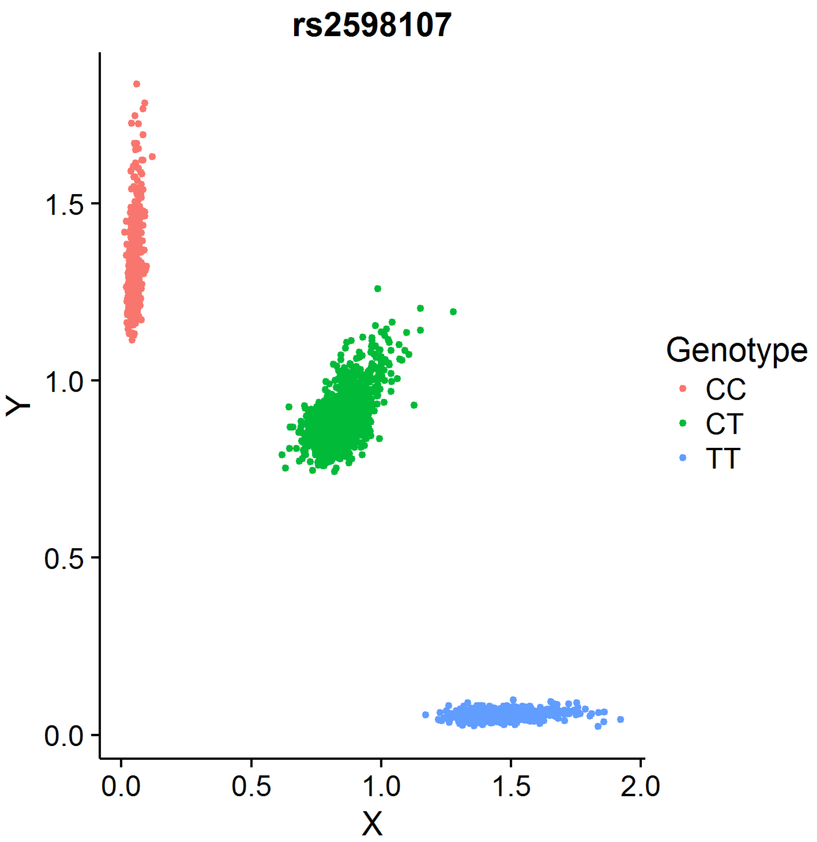
rs2598107
Y
1.5
1.0
0.5
0.0
0.0
0.5
1.0
1.5
2.0
X
Genotype
CC
CT
TT

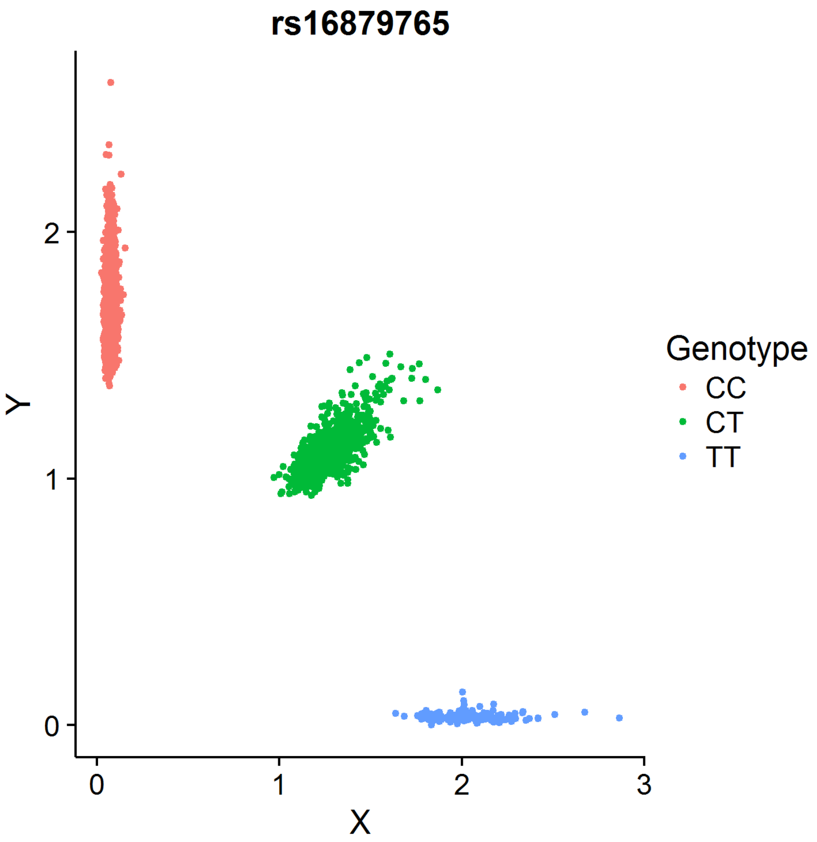
rs16879765
Y
2
1
0
0
1
2
3
X
Genotype
CC
CT
TT

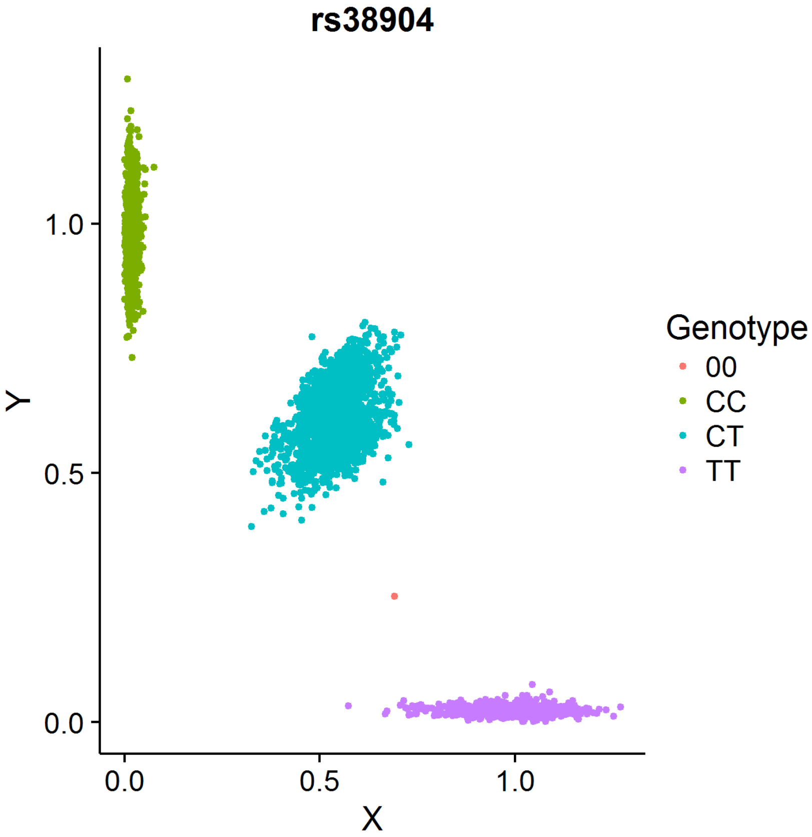
rs38904
Y
X
1.0
0.5
0.0
0.0
0.5
1.0
Genotype
00
CC
CT
TT

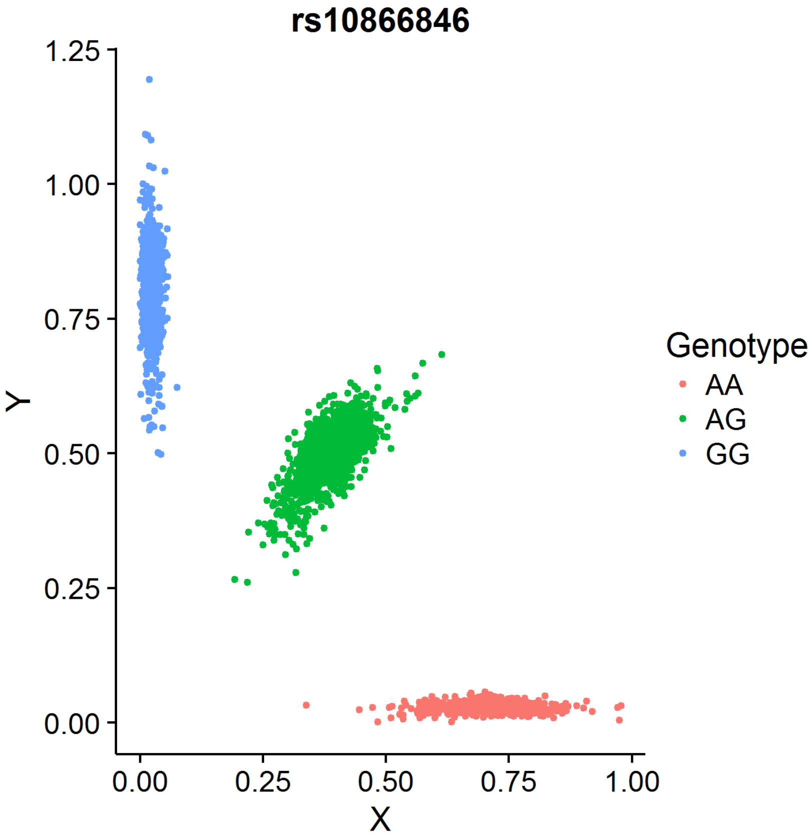
rs10866846
Y
X
1.25
1.00
0.75
0.50
0.25
0.00
0.00
0.25
0.50
0.75
1.00
Genotype
AA
AG
GG

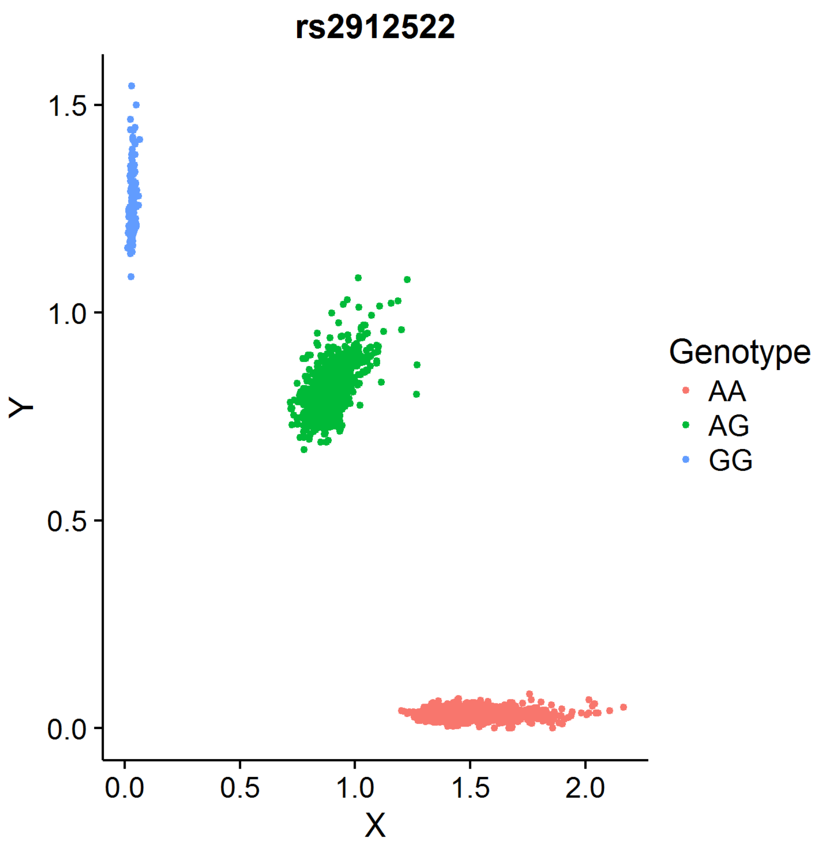
rs2912522
1.5
1.0
0.5
0.0
Y
0.0
0.5
1.0
1.5
2.0
X
Genotype
AA
AG
GG

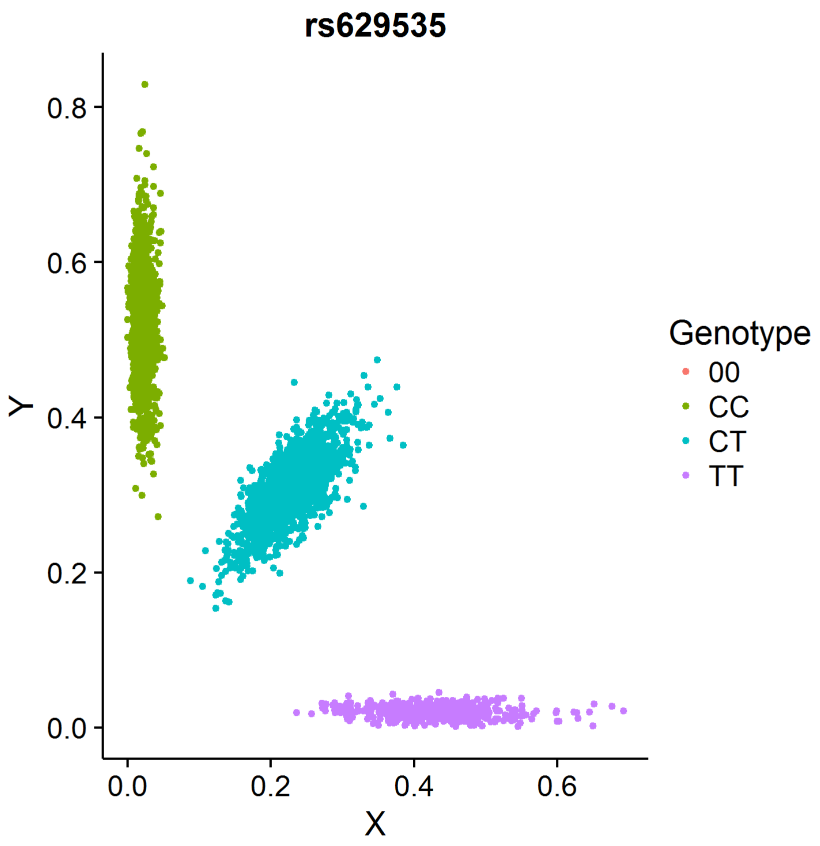
rs629535
0.8
0.6
0.4
0.2
0.0
Y
0.0
0.2
0.4
0.6
X
Genotype
00
CC
CT
TT

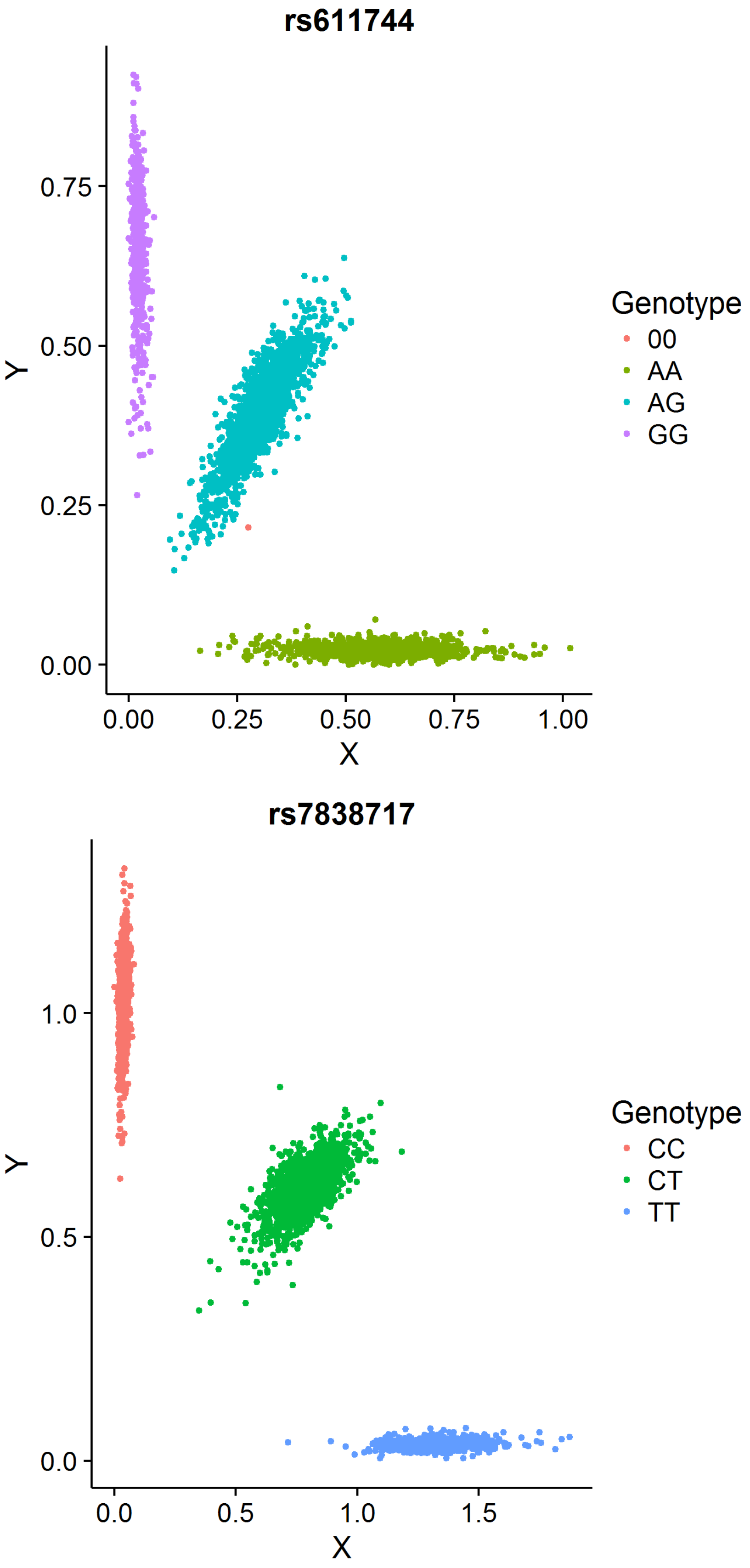
rs611744
0.00
0.25
0.50
0.75
Y
0.00
0.25
0.50
0.75
1.00
X
Genotype
00
AA
AG
GG
rs7838717
0.0
0.5
1.0
Y
0.0
0.5
1.0
1.5
X
Genotype
CC
CT
TT

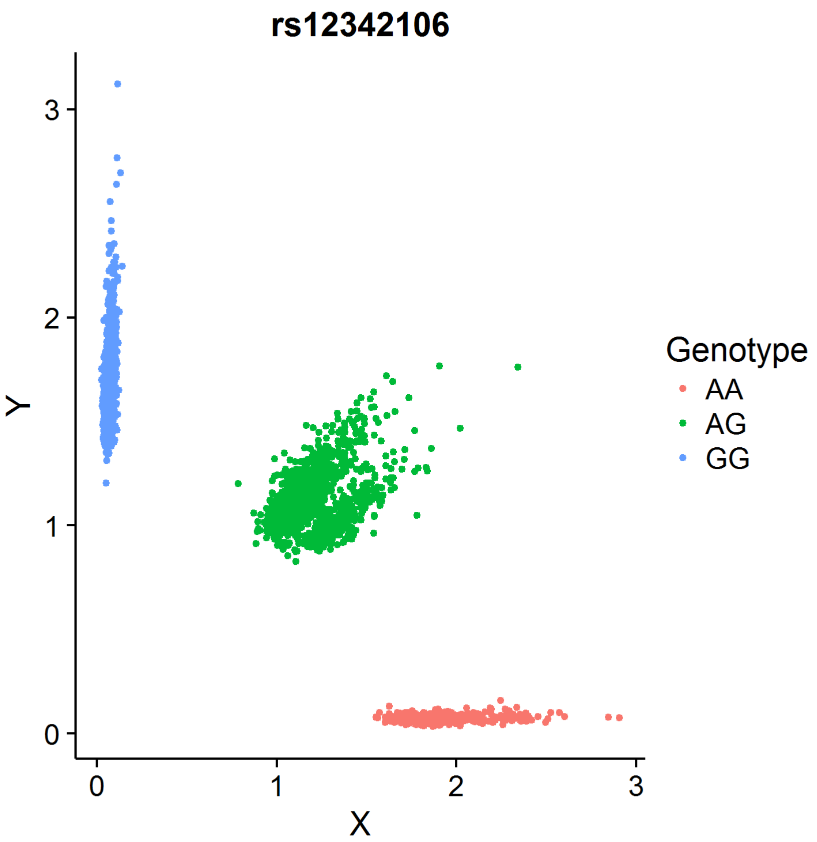
rs12342106
Y
X
Genotype
AA
AG
GG

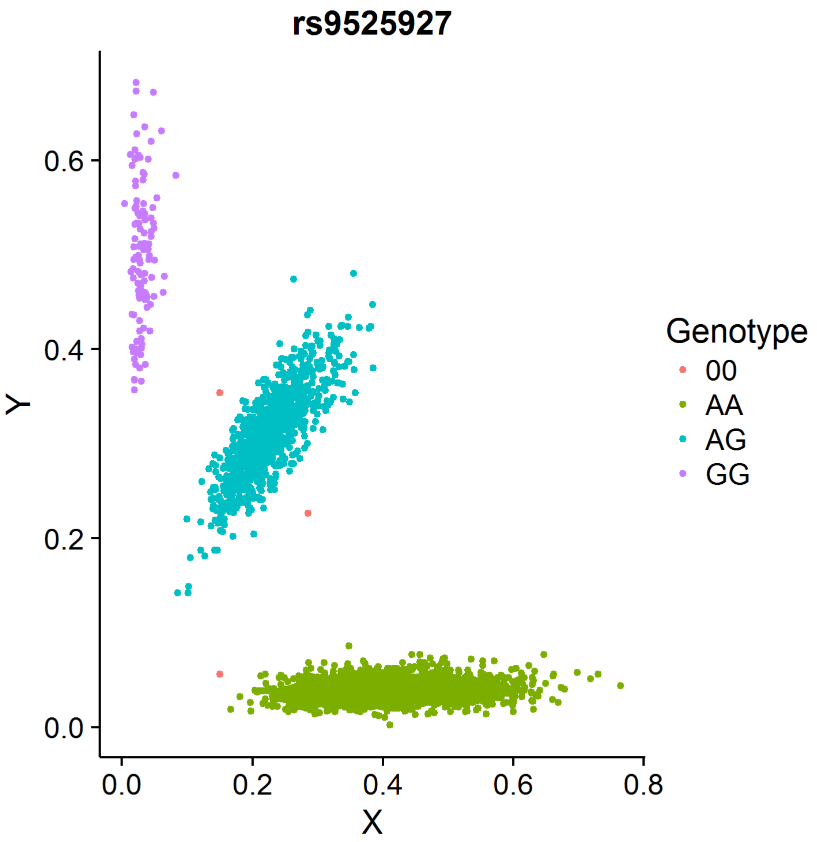
rs9525927
Y
X
Genotype
00
AA
AG
GG

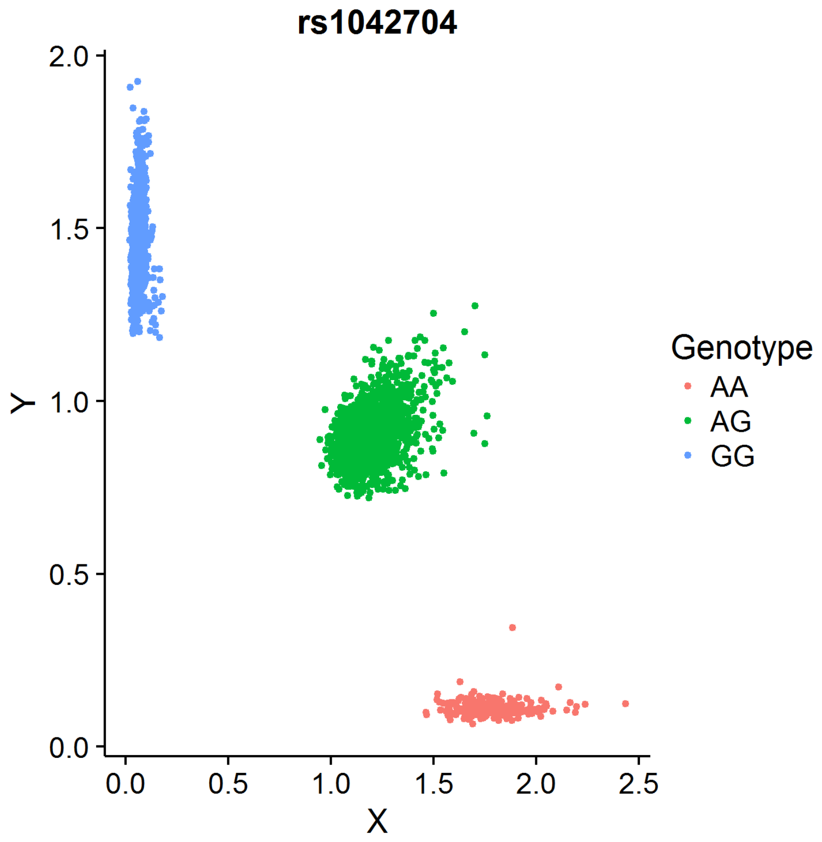
rs1042704
2.0
1.5
1.0
0.5
0.0
Y
0.0
0.5
1.0
1.5
2.0
2.5
X
Genotype
AA
AG
GG

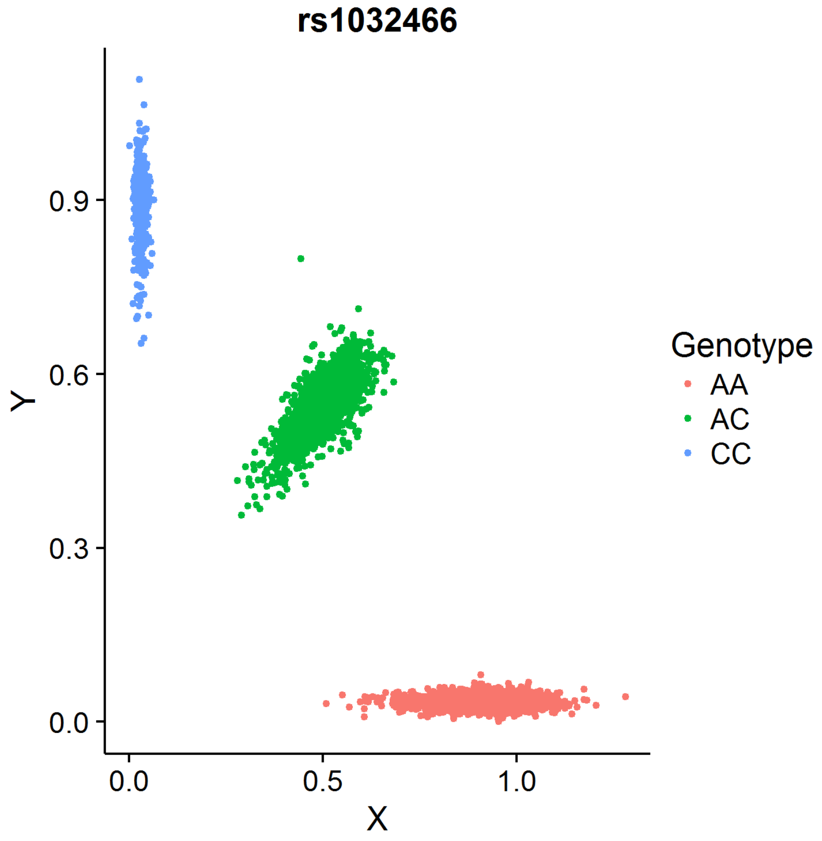
rs1032466
0.9
0.6
0.3
0.0
Y
0.0
0.5
1.0
X
Genotype
AA
AC
CC

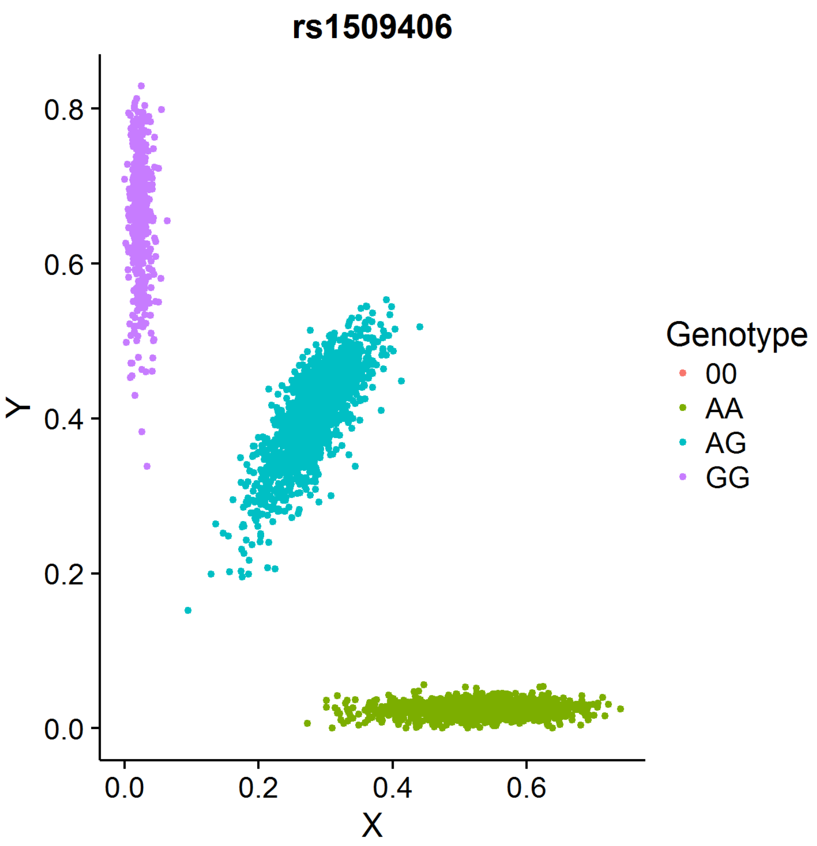
rs1509406
Y
0.0
0.2
0.4
0.6
0.8
X
0.0
0.2
0.4
0.6
Genotype
00
AA
AG
GG

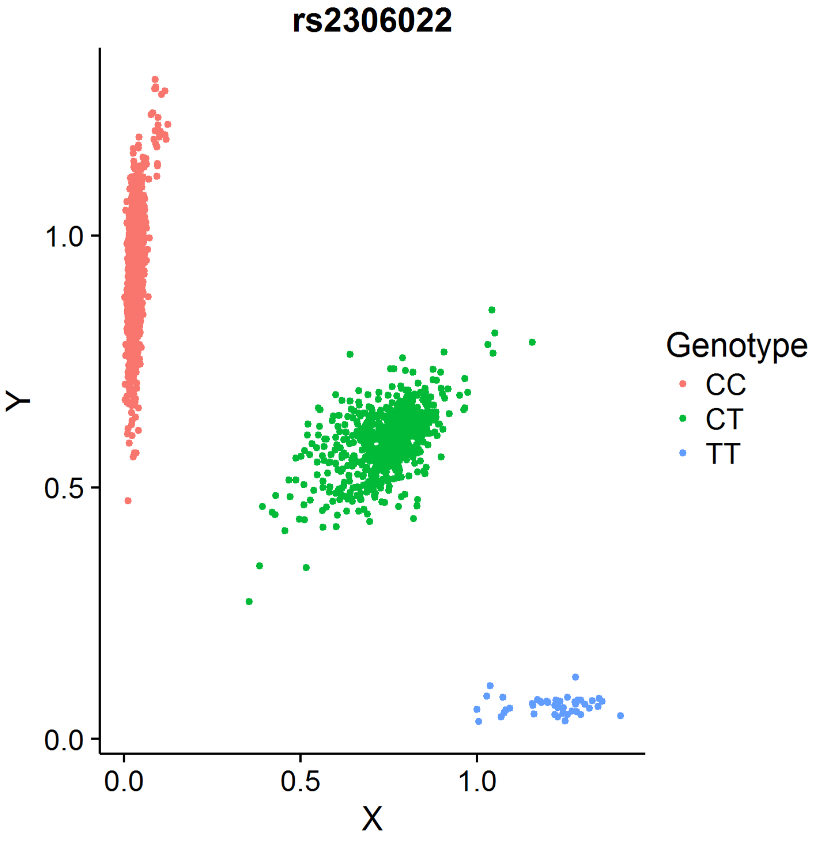
rs2306022
Y
0.0
0.5
1.0
X
0.0
0.5
1.0
Genotype
CC
CT
TT

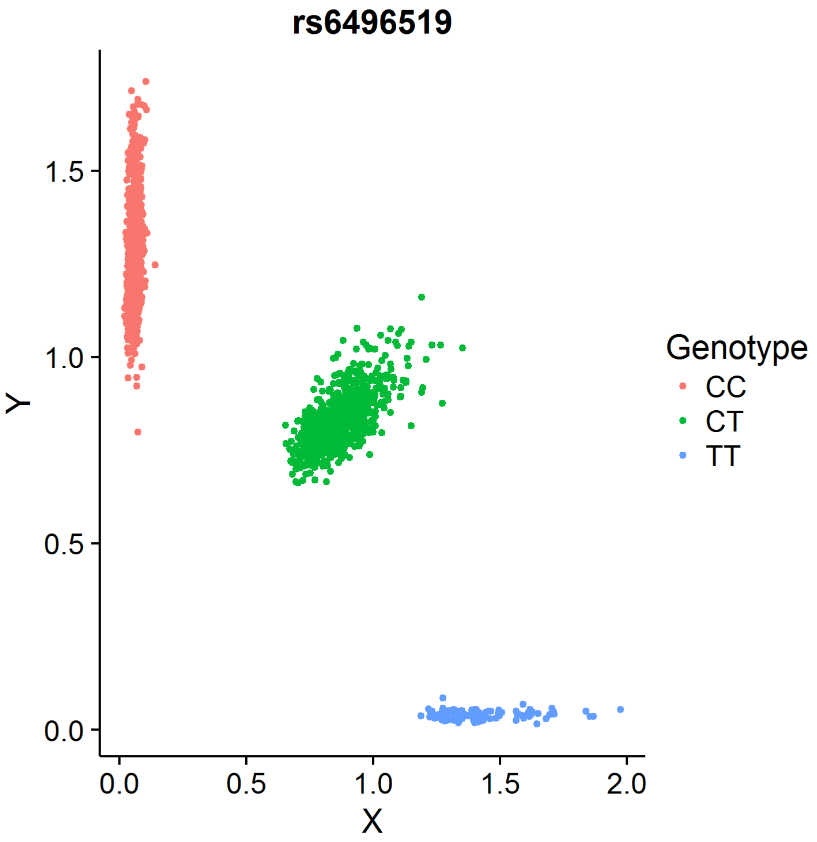
rs6496519
Y
1.5
1.0
0.5
0.0
X
0.0
0.5
1.0
1.5
2.0
Genotype
CC
CT
TT

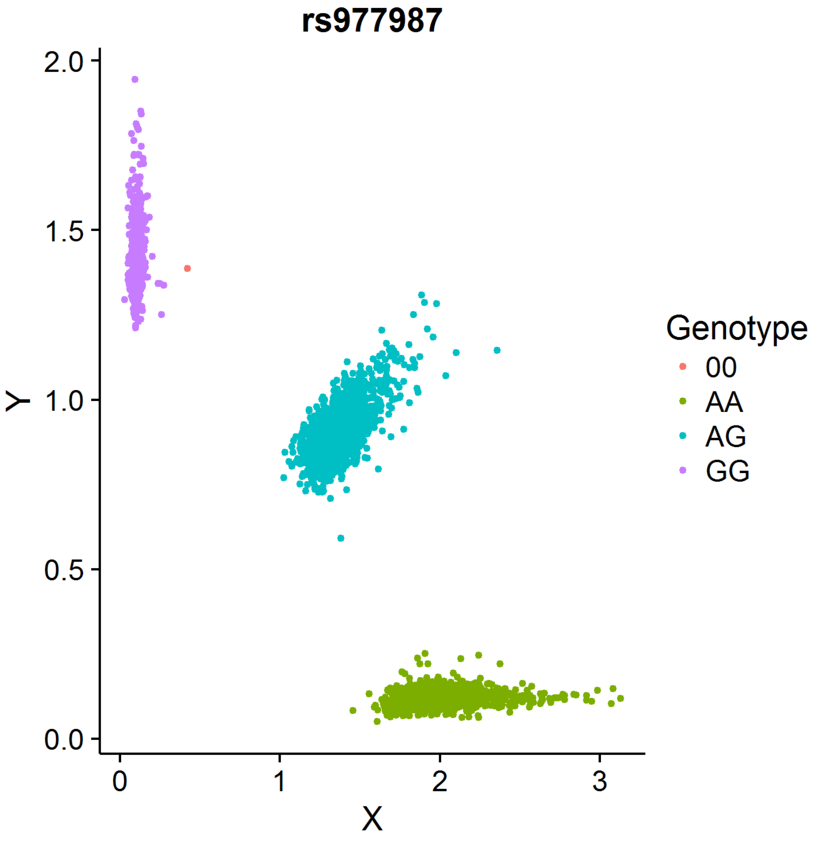
rs977987
Y
2.0
1.5
1.0
0.5
0.0
X
0
1
2
3
Genotype
00
AA
AG
GG

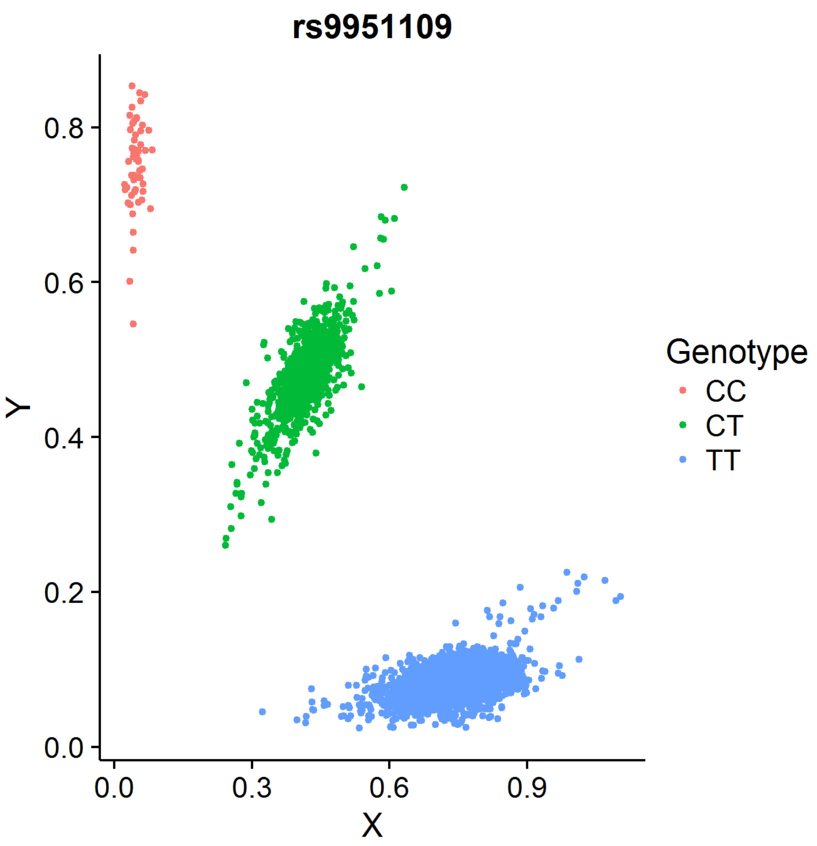
rs9951109
Y
X
0.0
0.2
0.4
0.6
0.8
0.0
0.3
0.6
0.9
Genotype
CC
CT
TT

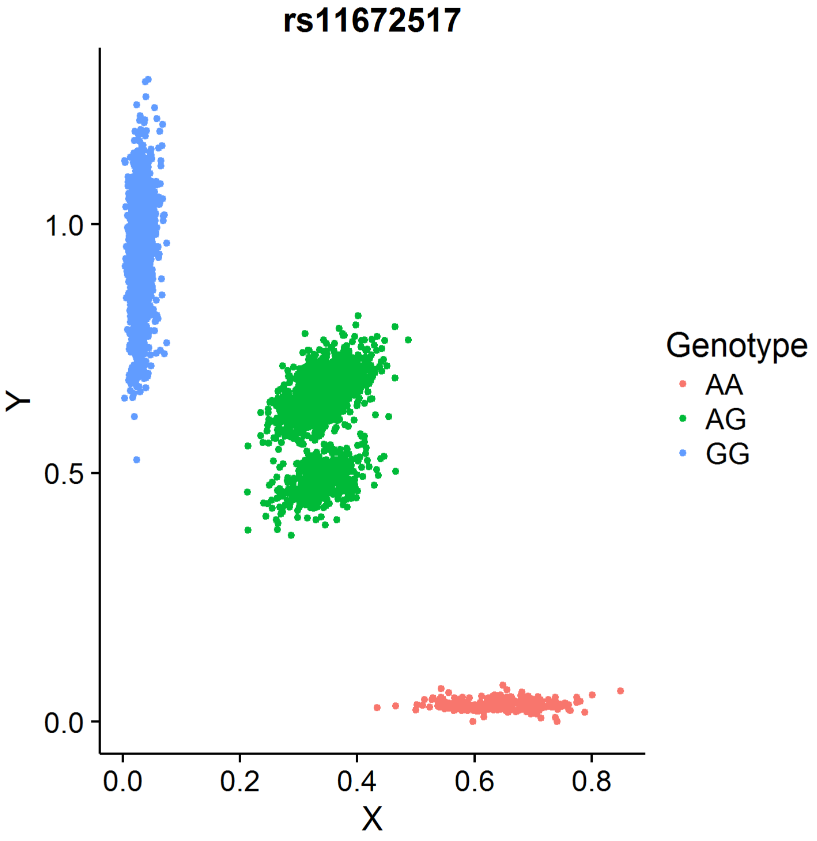
rs11672517
Y
X
0.0
0.5
1.0
0.0
0.2
0.4
0.6
0.8
Genotype
AA
AG
GG

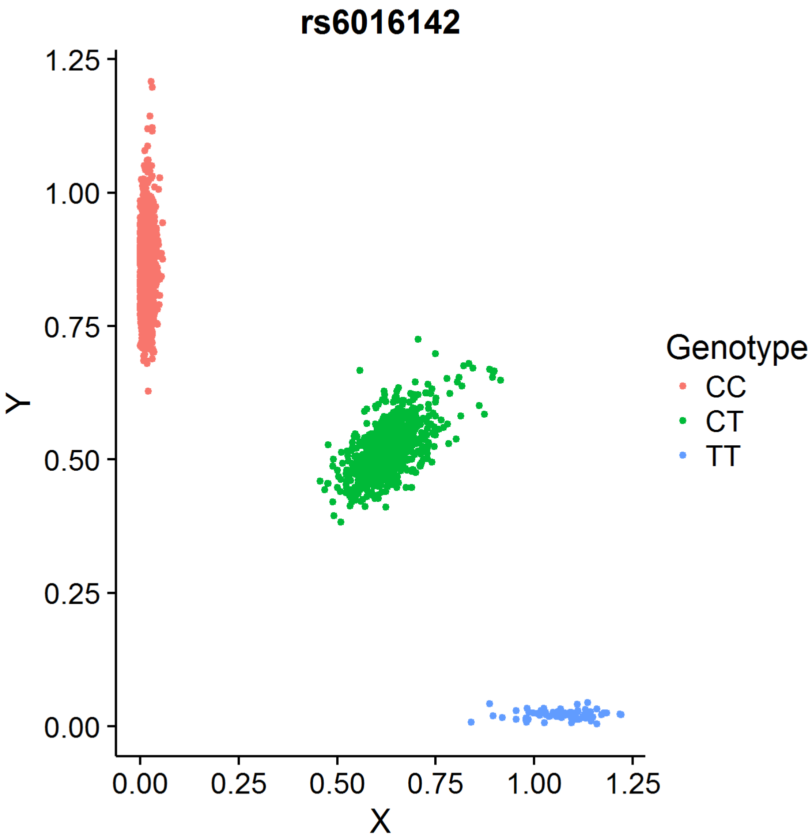
rs6016142
1.25
1.00
0.75
0.50
0.25
0.00
Y
0.00
0.25
0.50
0.75
1.00
1.25
X
Genotype
CC
CT
TT

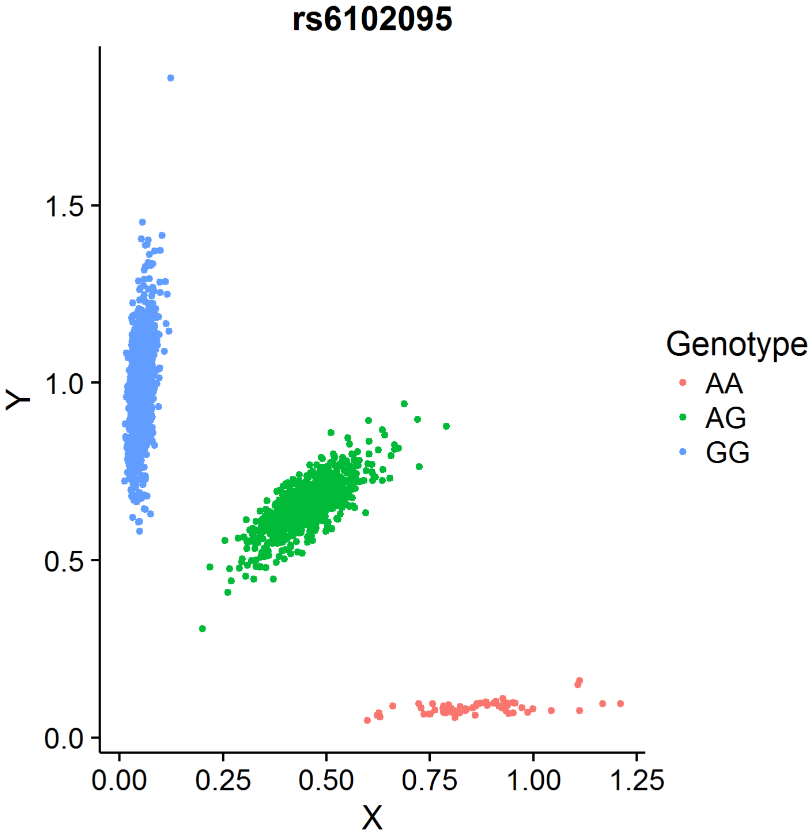
rs6102095
1.5
1.0
0.5
0.0
Y
0.00
0.25
0.50
0.75
1.00
1.25
X
Genotype
AA
AG
GG

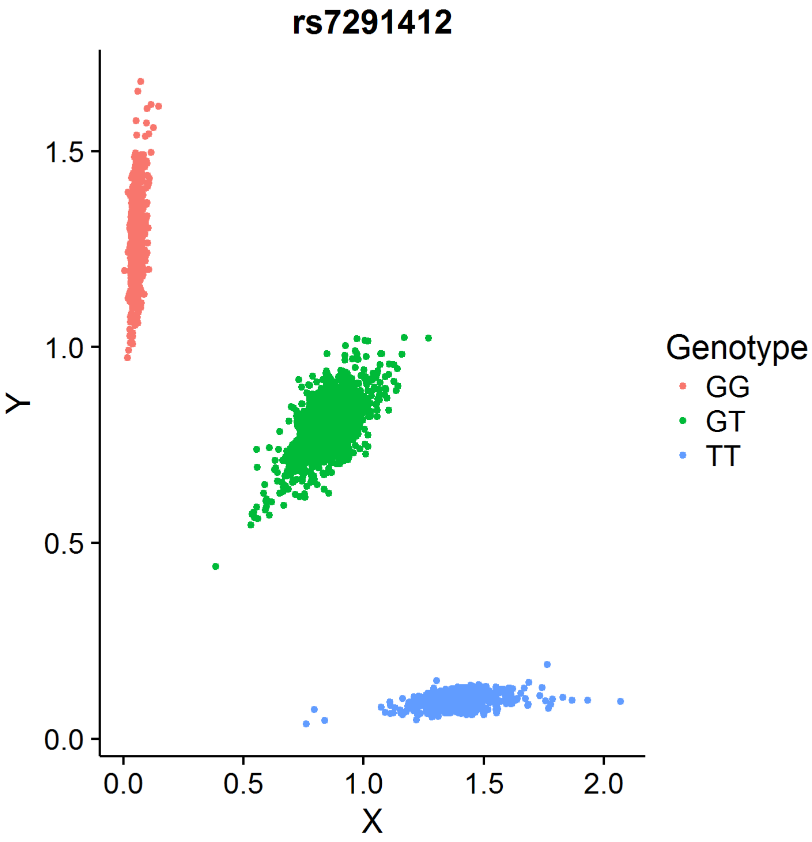

rs7291412
Y
1.5
1.0
0.5
0.0
0.0
0.5
1.0
1.5
2.0
X
Genotype
GG
GT
TT

Figure S5. Forest plots showing the magnitude and direction of odds ratio for the risk allele at all associated loci, across the different cohorts in which it was genotyped. The size of the box is proportional to the weight (size) of the dataset in the fixed-effects meta-analysis. The whiskers represent the 95% confidence intervals.

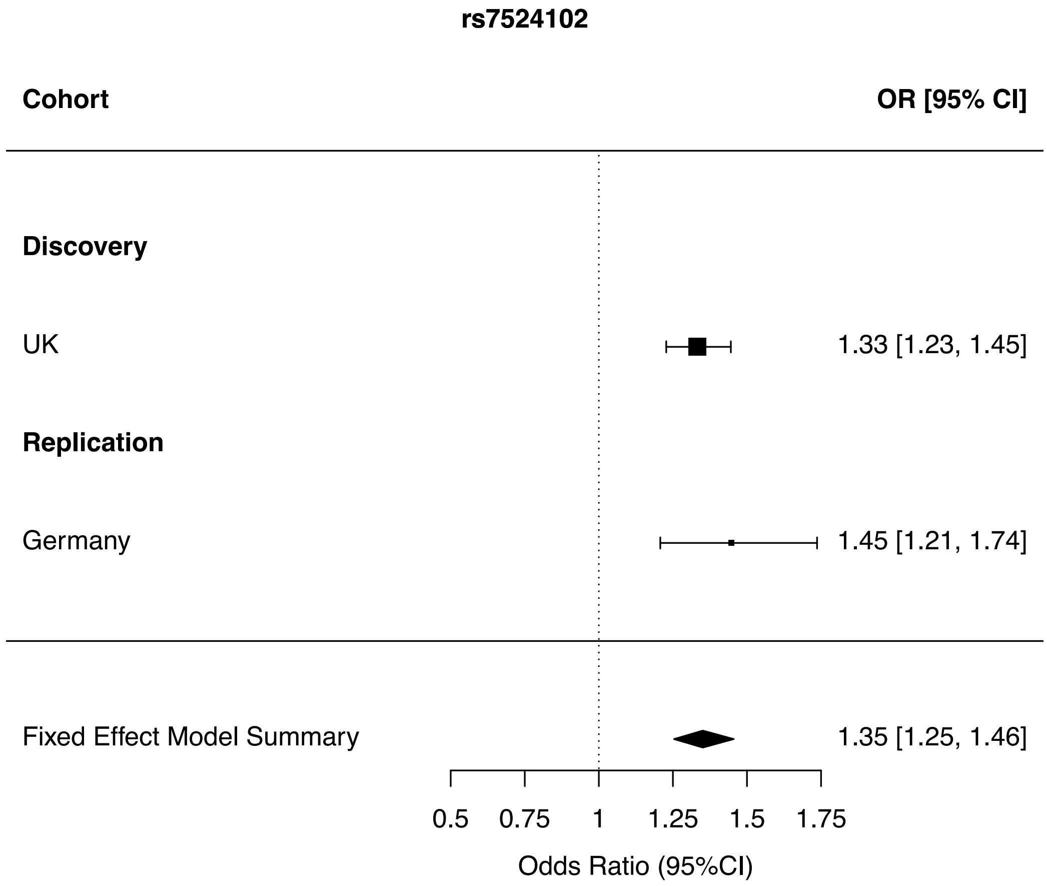

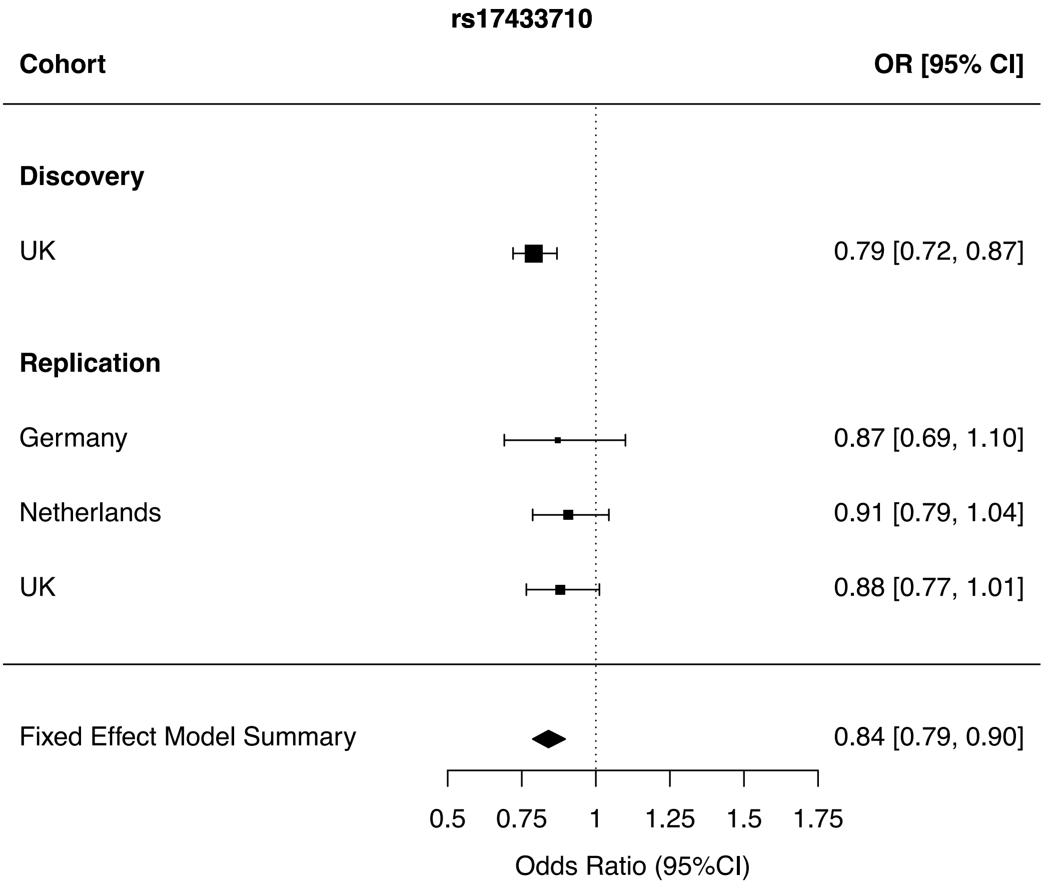
rs17433710
Cohort
OR [95% CI]
Discovery
UK
0.79 [0.72, 0.87]
Replication
Germany
0.87 [0.69, 1.10]
Netherlands
0.91 [0.79, 1.04]
UK
0.88 [0.77, 1.01]
Fixed Effect Model Summary
0.84 [0.79, 0.90]
0.5
0.75
1
1.25
1.5
1.75
Odds Ratio (95%CI)

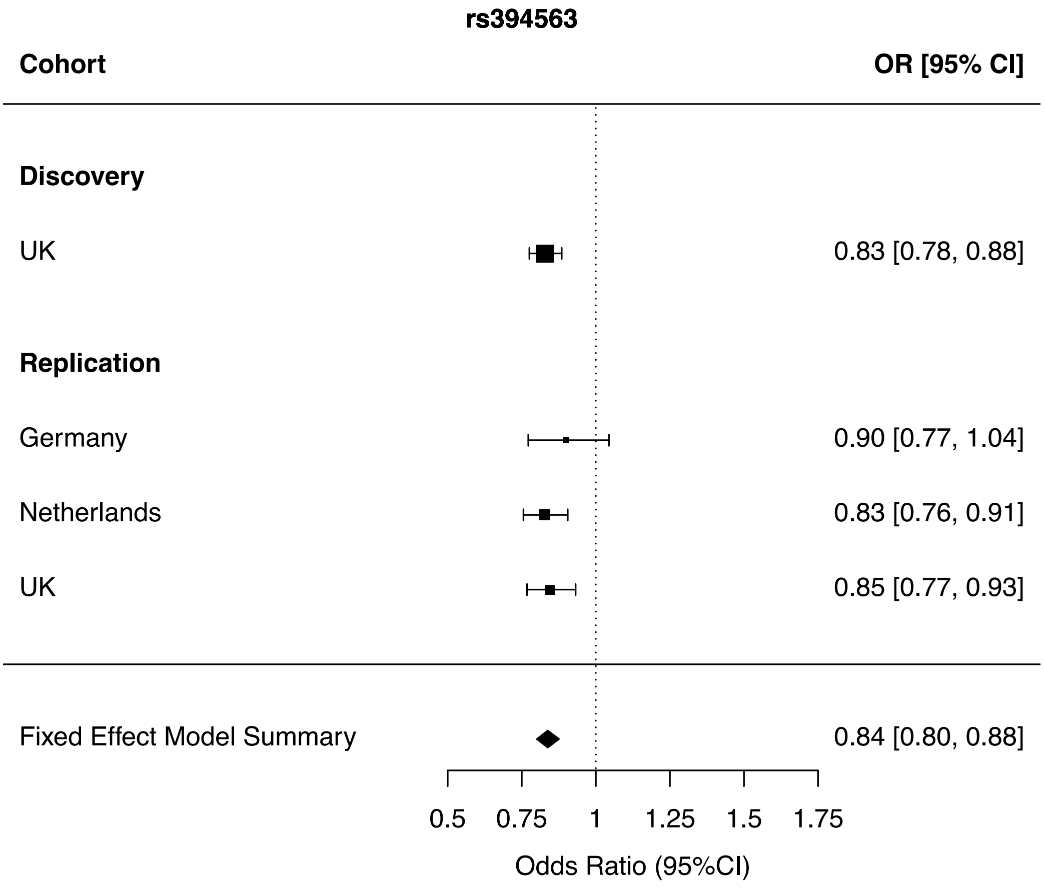
rs394563
Cohort
OR [95% CI]
Discovery
UK
0.83 [0.78, 0.88]
Replication
Germany
0.90 [0.77, 1.04]
Netherlands
0.83 [0.76, 0.91]
UK
0.85 [0.77, 0.93]
Fixed Effect Model Summary
0.84 [0.80, 0.88]
0.5
0.75
1
1.25
1.5
1.75
Odds Ratio (95%CI)

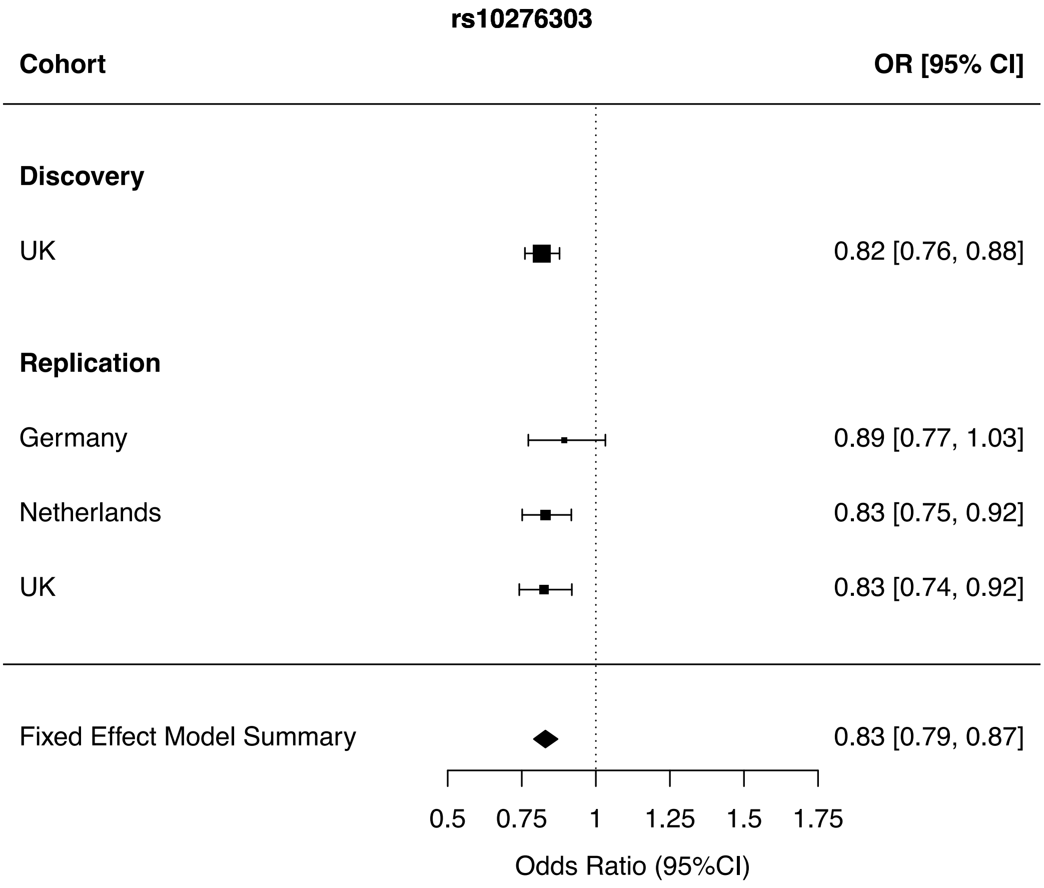
rs10276303
Cohort
OR [95% CI]
Discovery
UK
0.82 [0.76, 0.88]
Replication
Germany
0.89 [0.77, 1.03]
Netherlands
0.83 [0.75, 0.92]
UK
0.83 [0.74, 0.92]
Fixed Effect Model Summary
0.83 [0.79, 0.87]
0.5
0.75
1
1.25
1.5
1.75
Odds Ratio (95%CI)

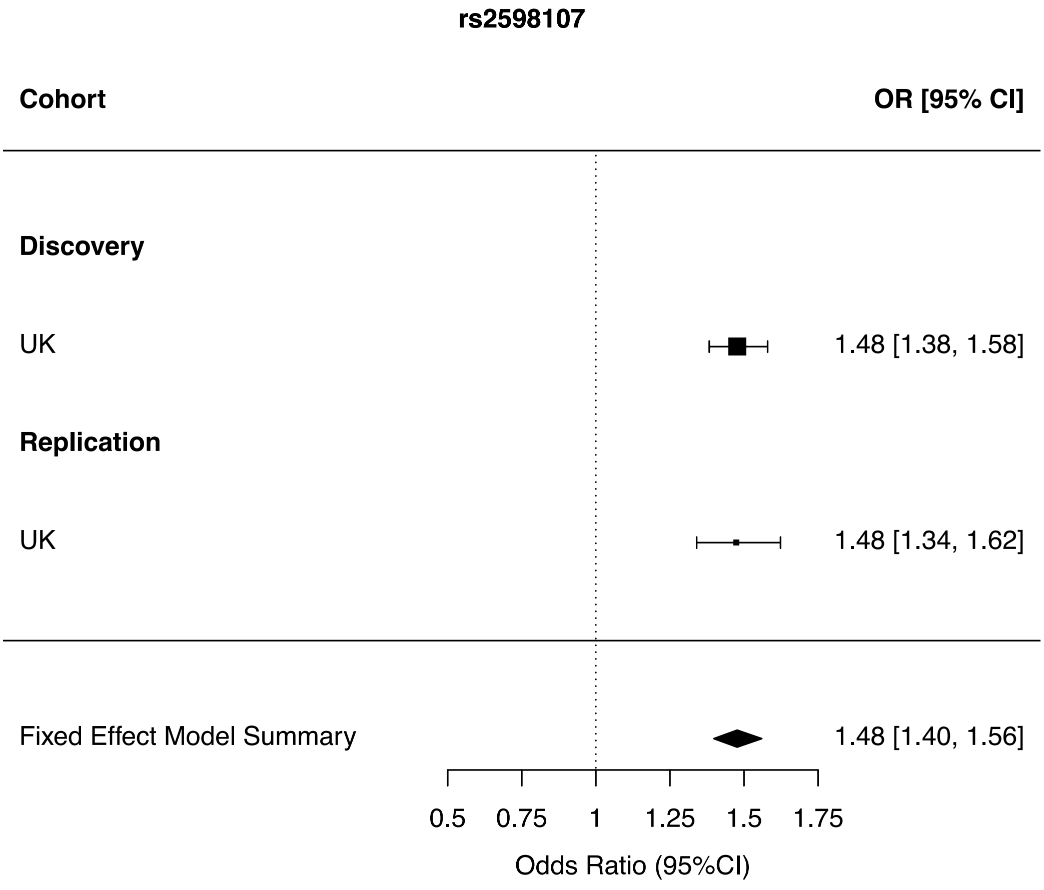
rs2598107
Cohort
OR [95% CI]
Discovery
UK
1.48 [1.38, 1.58]
Replication
UK
1.48 [1.34, 1.62]
Fixed Effect Model Summary
1.48 [1.40, 1.56]
0.5
0.75
1
1.25
1.5
1.75
Odds Ratio (95%CI)

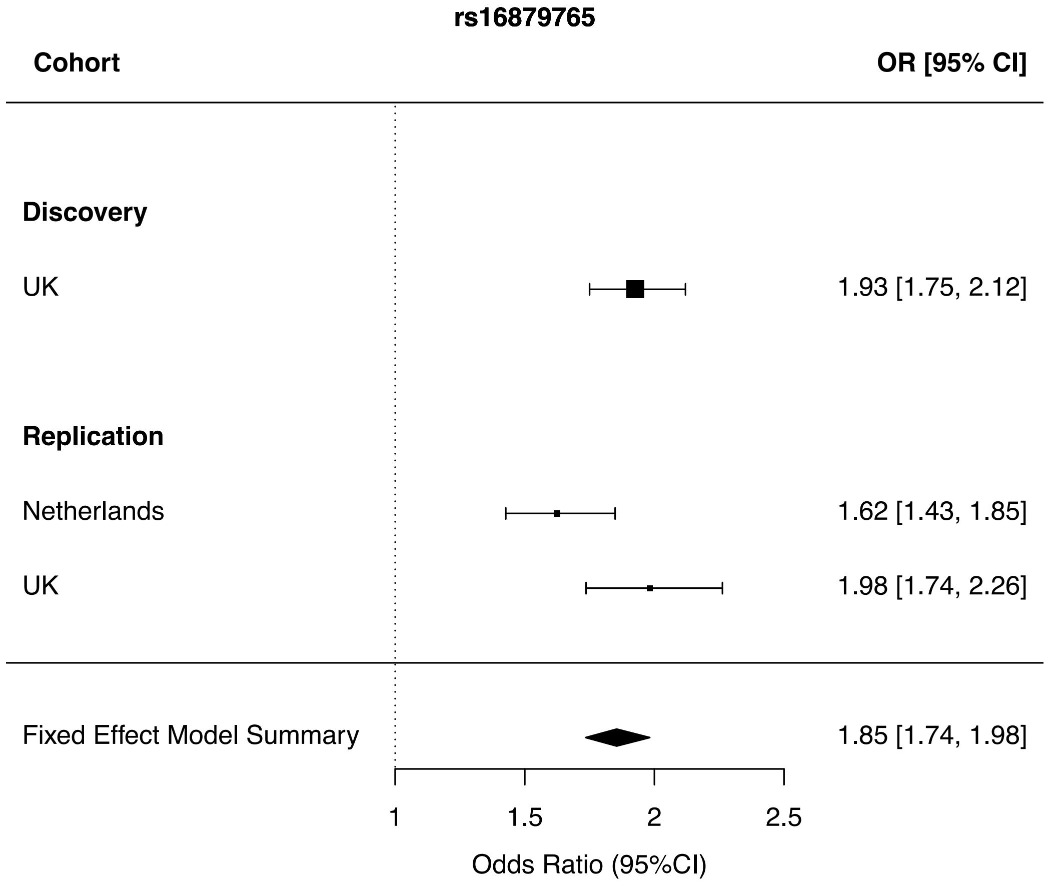
rs16879765
Cohort
OR [95% CI]
Discovery
UK
1.93 [1.75, 2.12]
Replication
Netherlands
1.62 [1.43, 1.85]
UK
1.98 [1.74, 2.26]
Fixed Effect Model Summary
1.85 [1.74, 1.98]
1
1.5
2
2.5
Odds Ratio (95%CI)

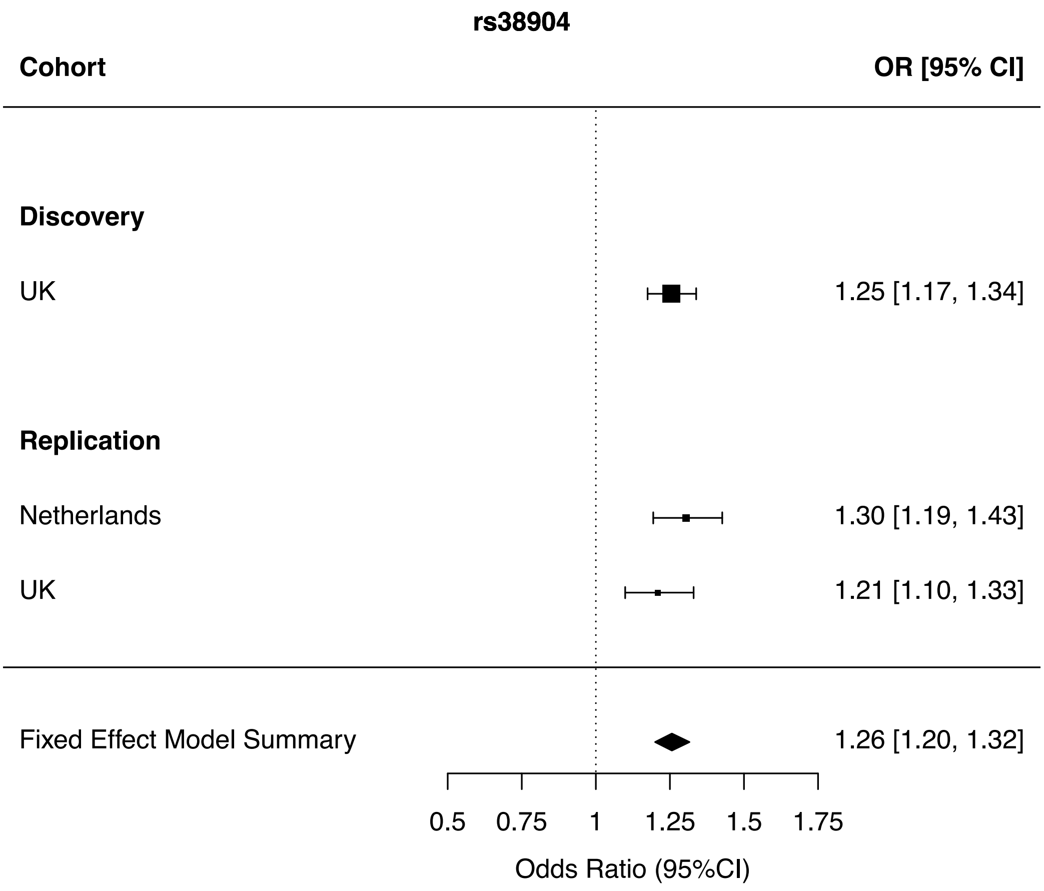
rs38904
Cohort
OR [95% CI]
Discovery
UK
1.25 [1.17, 1.34]
Replication
Netherlands
1.30 [1.19, 1.43]
UK
1.21 [1.10, 1.33]
Fixed Effect Model Summary
1.26 [1.20, 1.32]
0.5
0.75
1
1.25
1.5
1.75
Odds Ratio (95%CI)

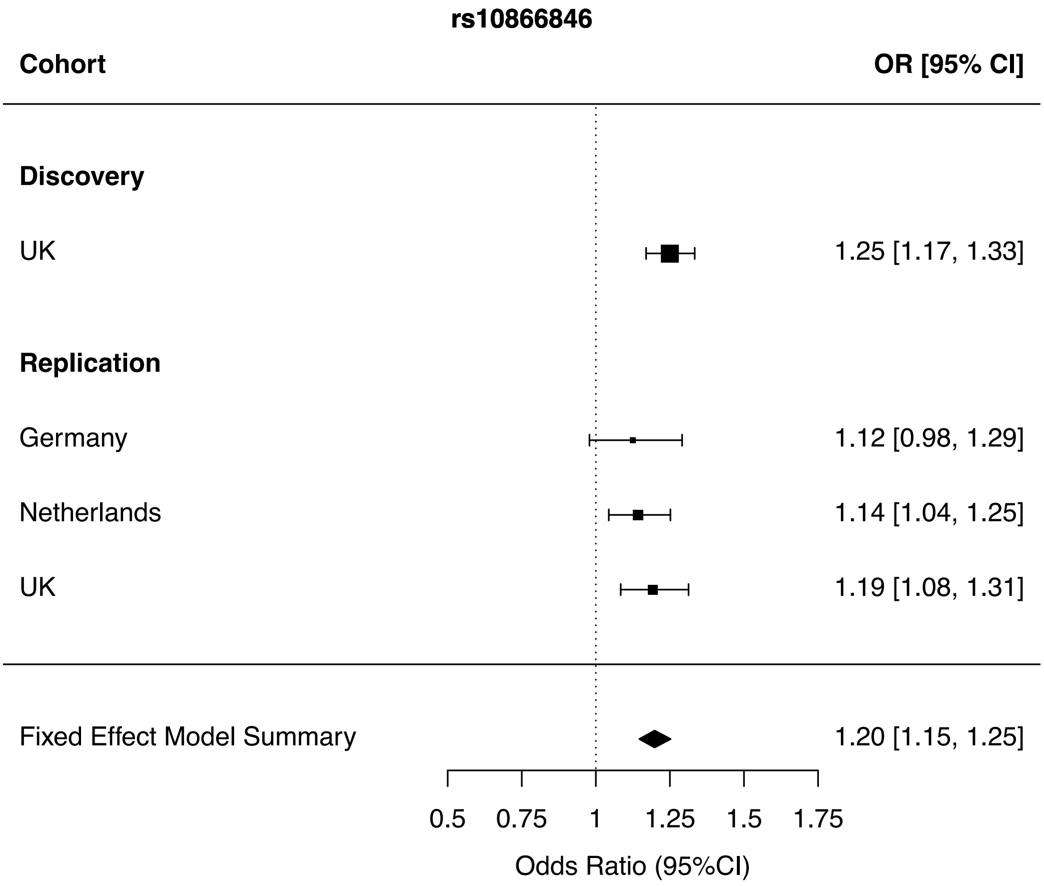
rs10866846
Cohort
OR [95% CI]
Discovery
UK
1.25 [1.17, 1.33]
Replication
Germany
1.12 [0.98, 1.29]
Netherlands
1.14 [1.04, 1.25]
UK
1.19 [1.08, 1.31]
Fixed Effect Model Summary
1.20 [1.15, 1.25]
0.5
0.75
1
1.25
1.5
1.75
Odds Ratio (95%CI)

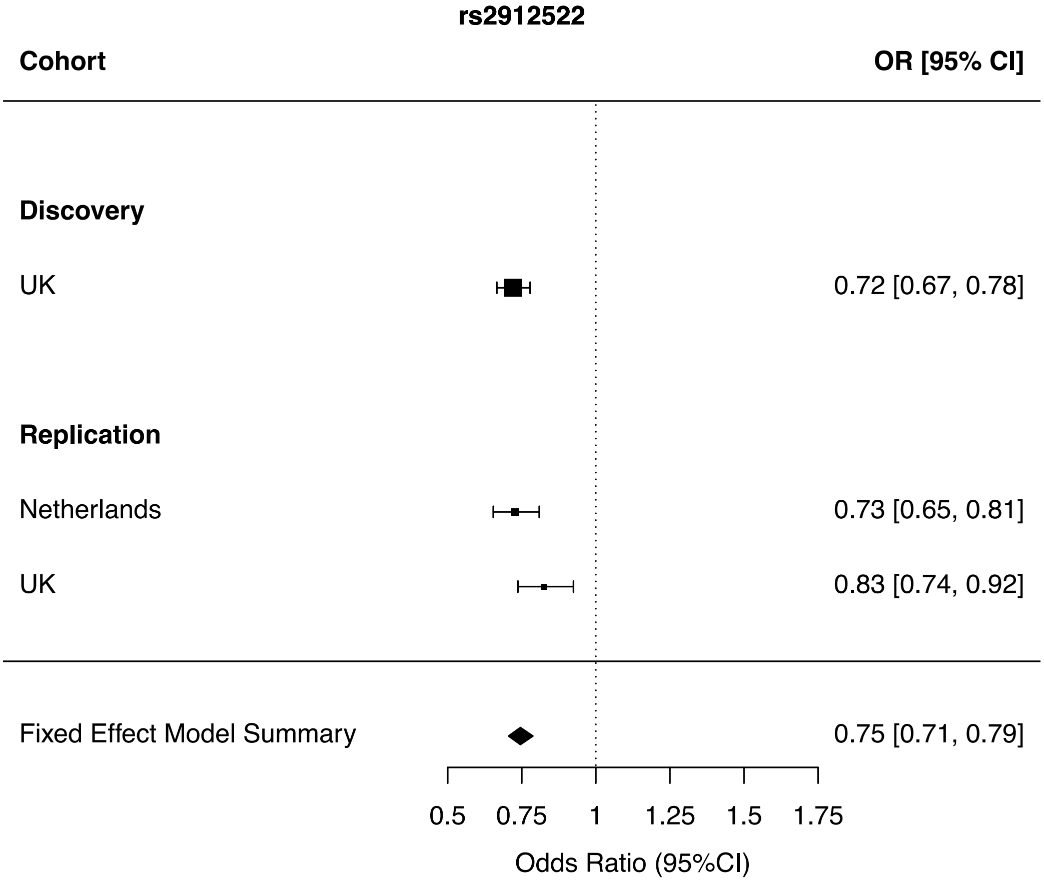
rs2912522
Cohort
OR [95% CI]
Discovery
UK
0.72 [0.67, 0.78]
Replication
Netherlands
0.73 [0.65, 0.81]
UK
0.83 [0.74, 0.92]
Fixed Effect Model Summary
0.75 [0.71, 0.79]
0.5
0.75
1
1.25
1.5
1.75
Odds Ratio (95%CI)

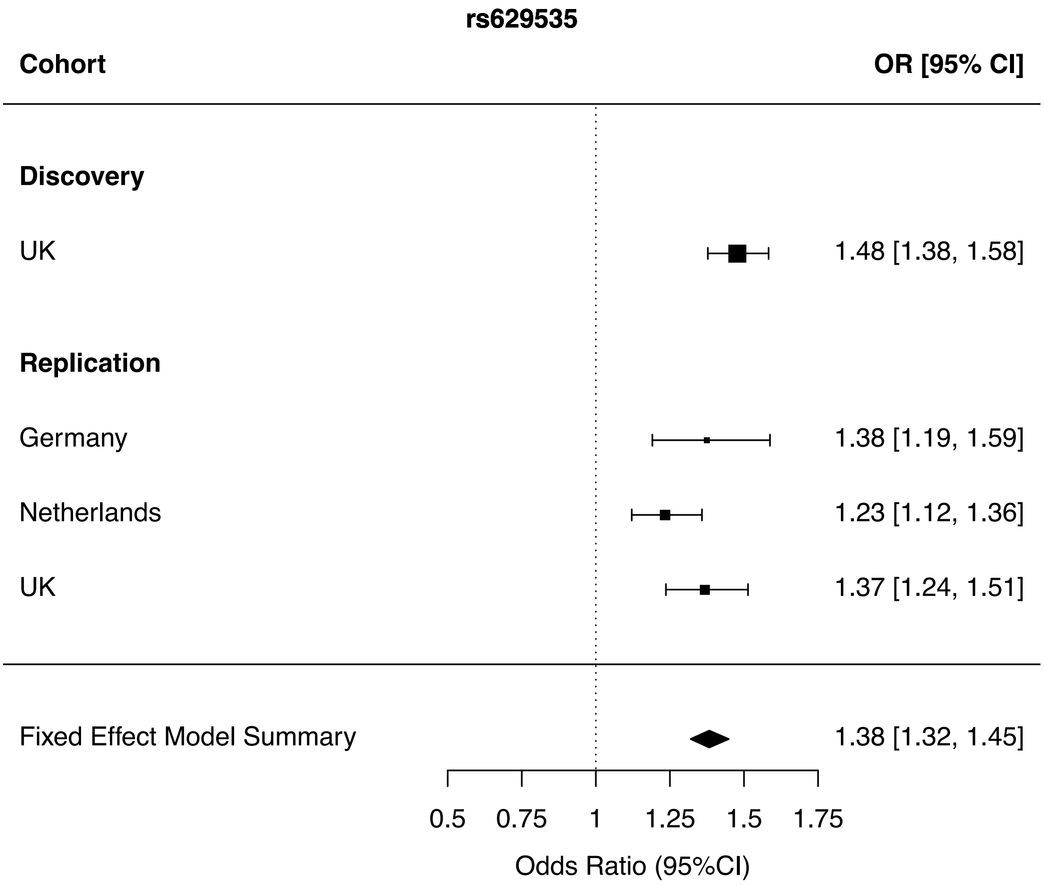
rs629535
Cohort
OR [95% CI]
Discovery
UK
1.48 [1.38, 1.58]
Replication
Germany
1.38 [1.19, 1.59]
Netherlands
1.23 [1.12, 1.36]
UK
1.37 [1.24, 1.51]
Fixed Effect Model Summary
1.38 [1.32, 1.45]
0.5
0.75
1
1.25
1.5
1.75
Odds Ratio (95%CI)

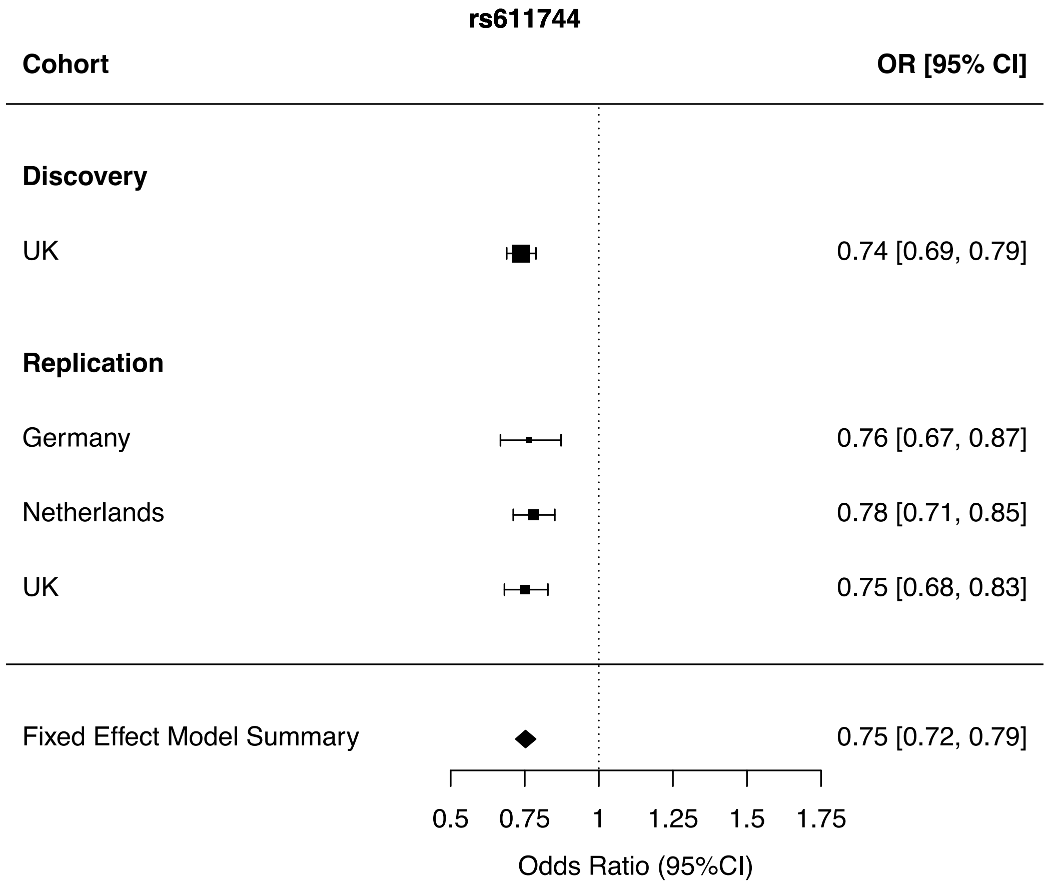
rs611744
Cohort
OR [95% CI]
Discovery
UK
0.74 [0.69, 0.79]
Replication
Germany
0.76 [0.67, 0.87]
Netherlands
0.78 [0.71, 0.85]
UK
0.75 [0.68, 0.83]
Fixed Effect Model Summary
0.75 [0.72, 0.79]
0.5
0.75
1
1.25
1.5
1.75
Odds Ratio (95%CI)

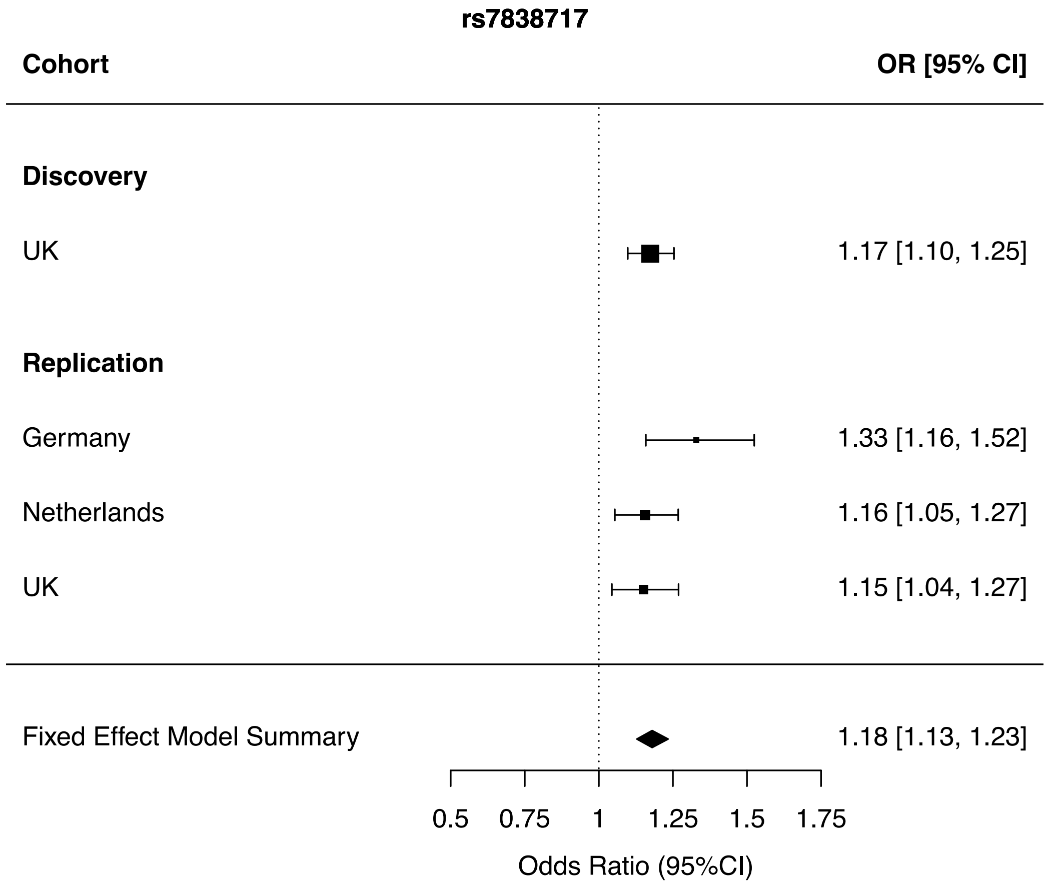
rs7838717
Cohort
OR [95% CI]
Discovery
UK
1.17 [1.10, 1.25]
Replication
Germany
1.33 [1.16, 1.52]
Netherlands
1.16 [1.05, 1.27]
UK
1.15 [1.04, 1.27]
Fixed Effect Model Summary
1.18 [1.13, 1.23]
0.5
0.75
1
1.25
1.5
1.75
Odds Ratio (95%CI)

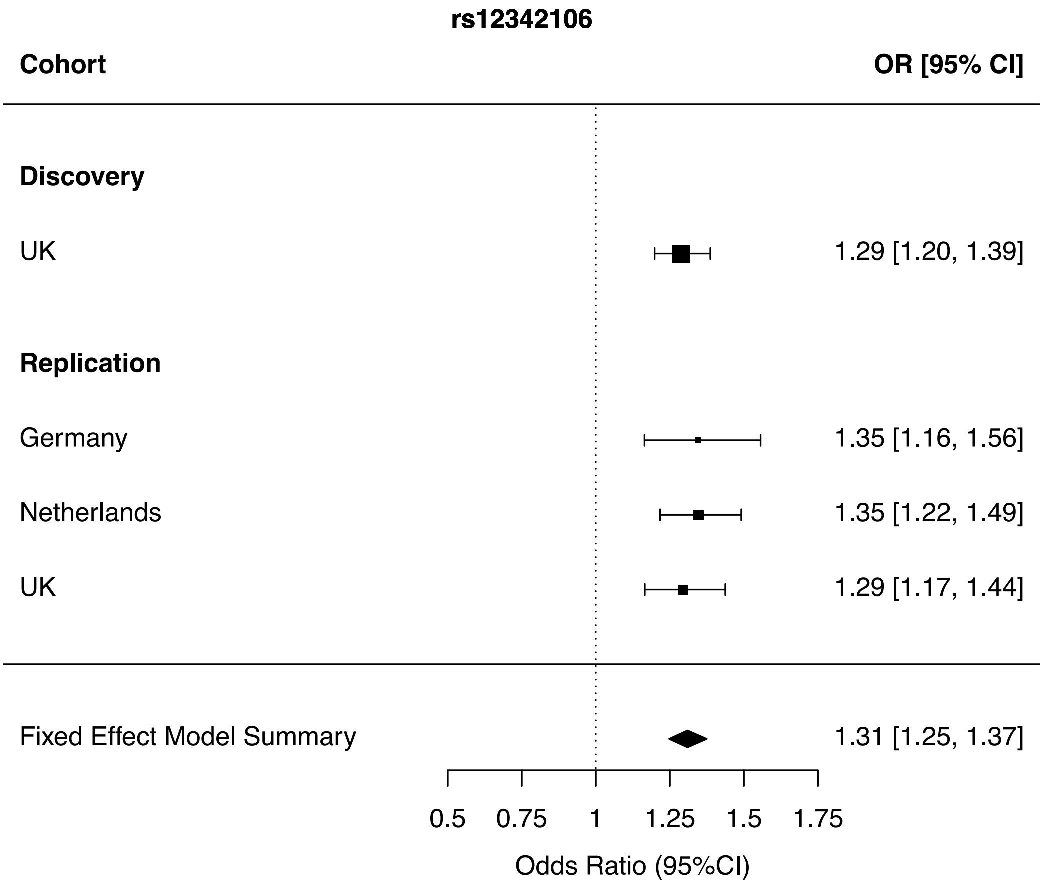
rs12342106
Cohort
OR [95% CI]
Discovery
UK
1.29 [1.20, 1.39]
Replication
Germany
1.35 [1.16, 1.56]
Netherlands
1.35 [1.22, 1.49]
UK
1.29 [1.17, 1.44]
Fixed Effect Model Summary
1.31 [1.25, 1.37]
0.5
0.75
1
1.25
1.5
1.75
Odds Ratio (95%CI)

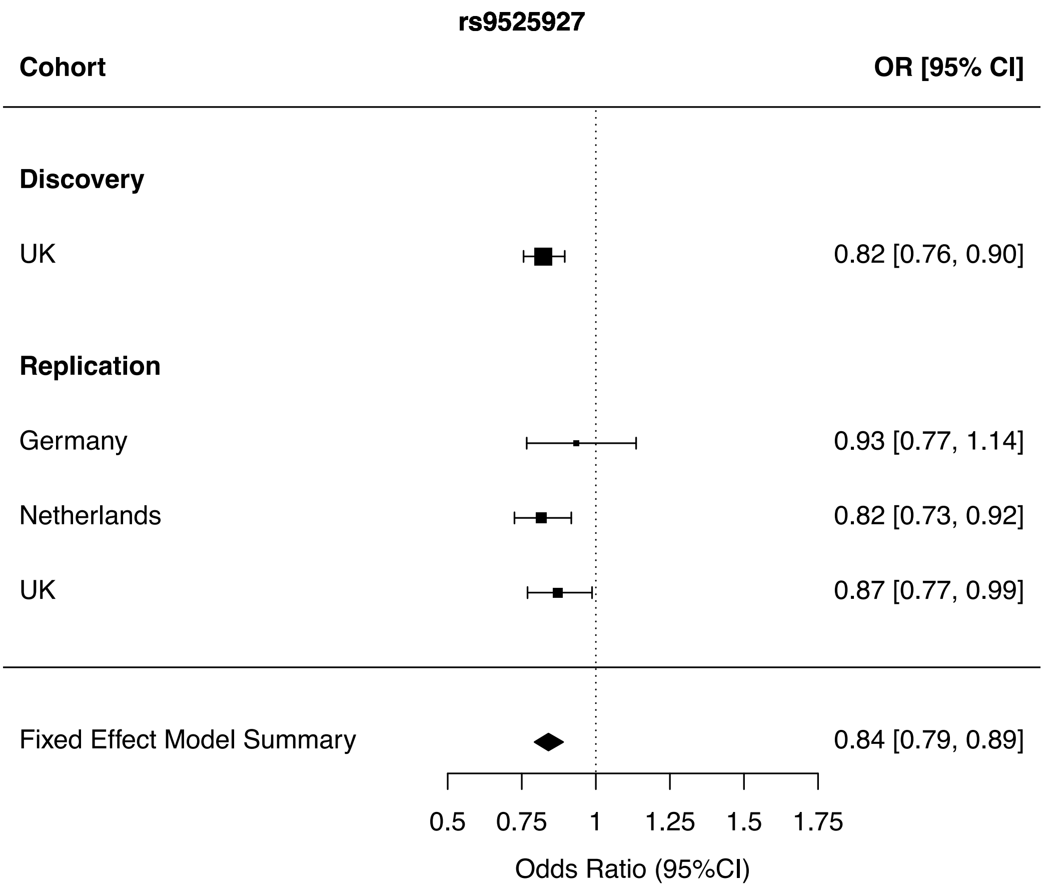
rs9525927
Cohort
OR [95% CI]
Discovery
UK
0.82 [0.76, 0.90]
Replication
Germany
0.93 [0.77, 1.14]
Netherlands
0.82 [0.73, 0.92]
UK
0.87 [0.77, 0.99]
Fixed Effect Model Summary
0.84 [0.79, 0.89]
0.5
0.75
1
1.25
1.5
1.75
Odds Ratio (95%CI)

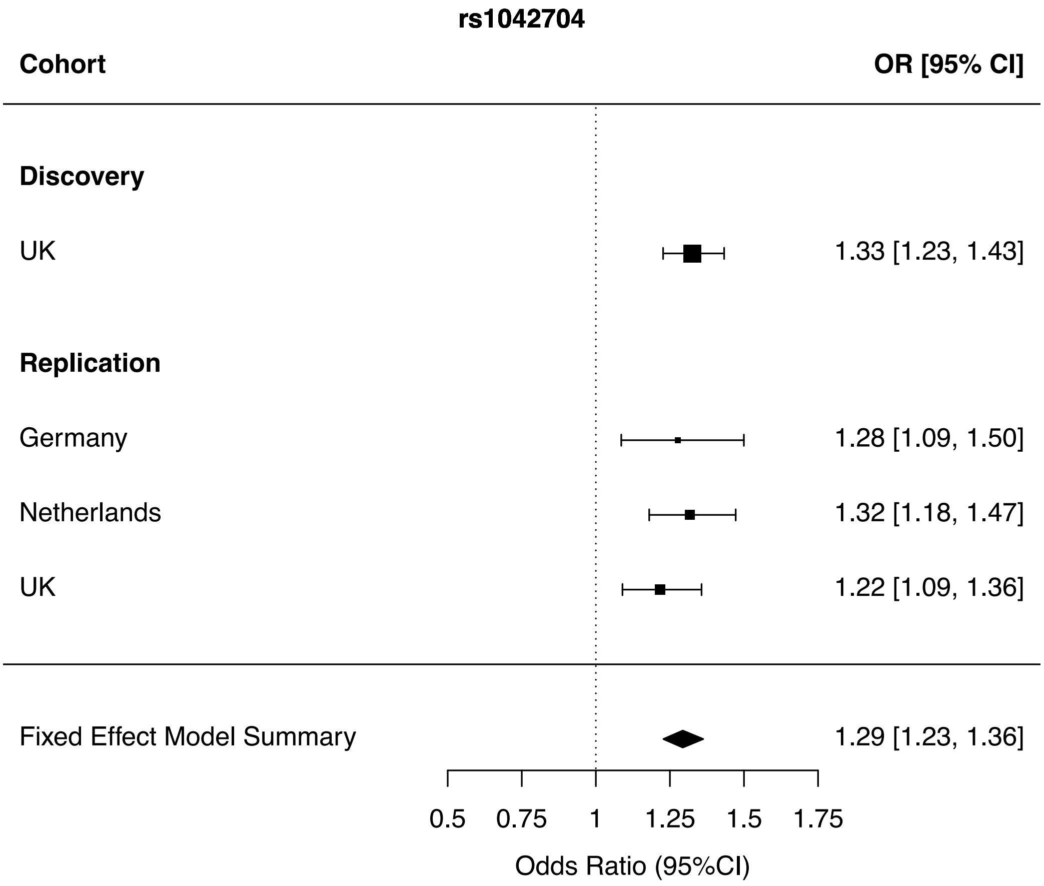
rs1042704
Cohort
OR [95% CI]
Discovery
UK
1.33 [1.23, 1.43]
Replication
Germany
1.28 [1.09, 1.50]
Netherlands
1.32 [1.18, 1.47]
UK
1.22 [1.09, 1.36]
Fixed Effect Model Summary
1.29 [1.23, 1.36]
0.5
0.75
1
1.25
1.5
1.75
Odds Ratio (95%CI)

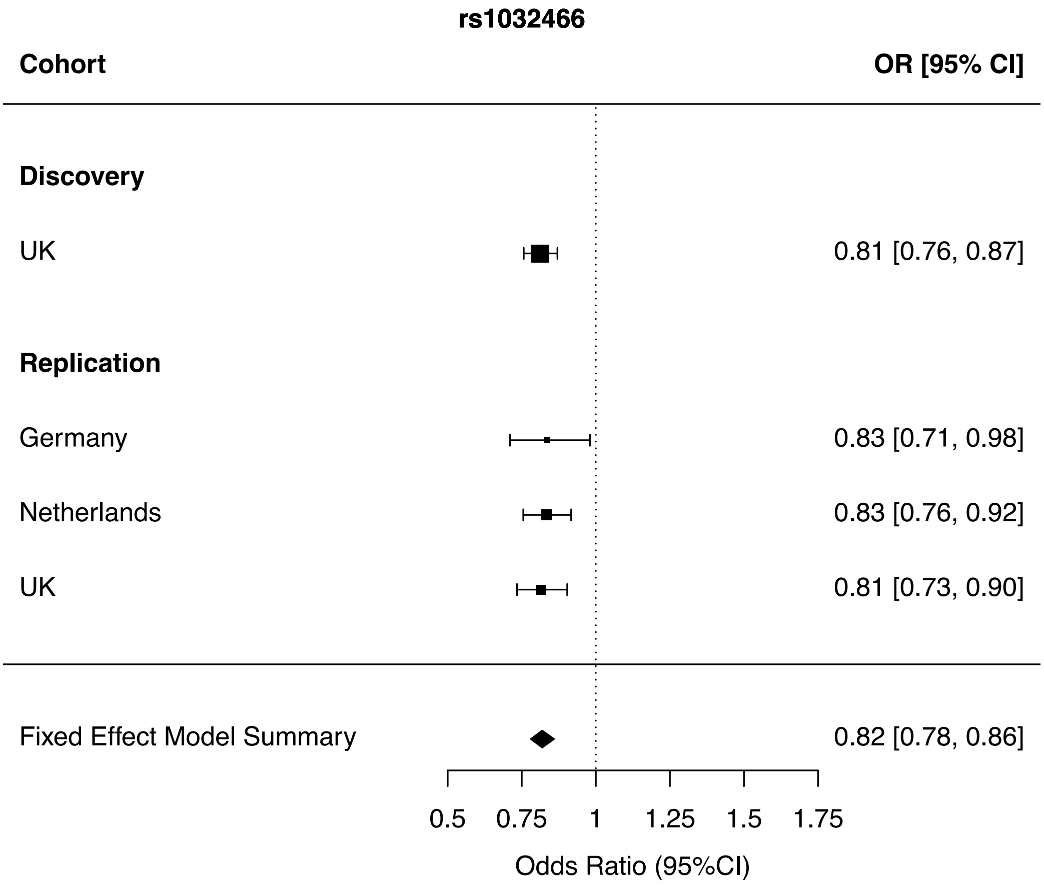
rs1032466
Cohort
OR [95% CI]
Discovery
UK
0.81 [0.76, 0.87]
Replication
Germany
0.83 [0.71, 0.98]
Netherlands
0.83 [0.76, 0.92]
UK
0.81 [0.73, 0.90]
Fixed Effect Model Summary
0.82 [0.78, 0.86]
0.5
0.75
1
1.25
1.5
1.75
Odds Ratio (95%CI)

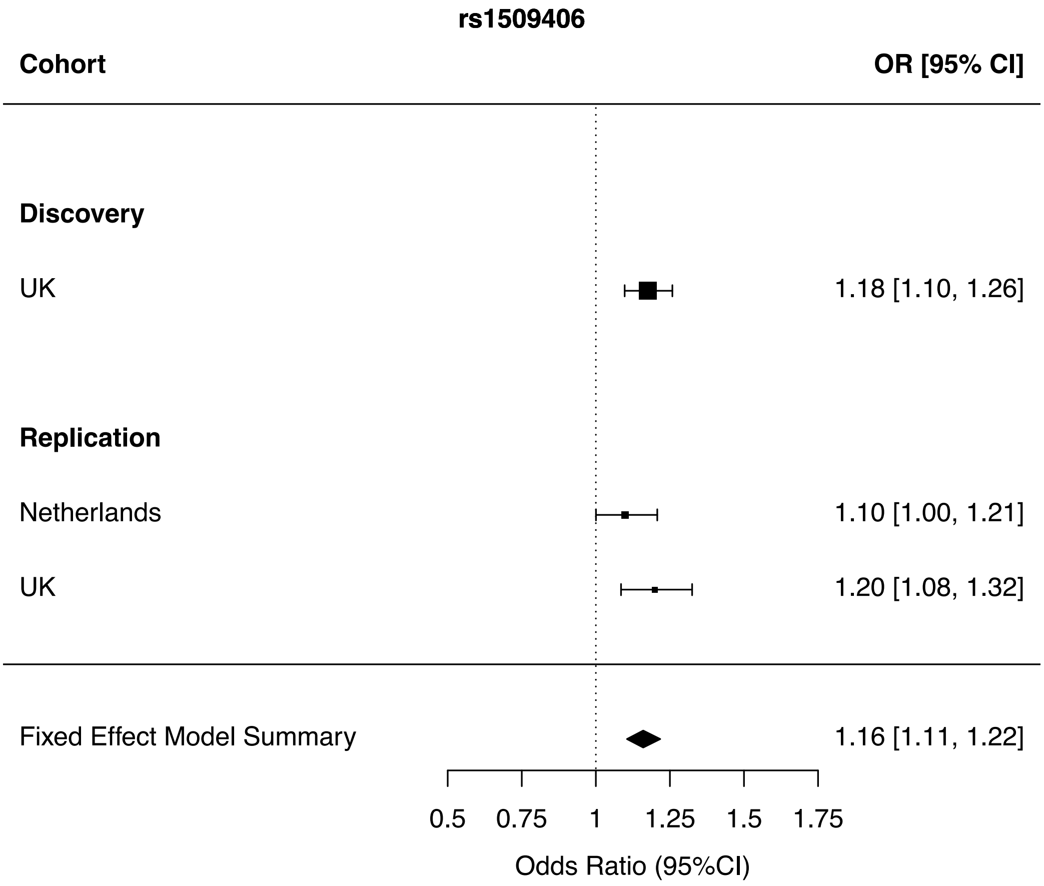
rs1509406
Cohort
OR [95% CI]
Discovery
UK
1.18 [1.10, 1.26]
Replication
Netherlands
1.10 [1.00, 1.21]
UK
1.20 [1.08, 1.32]
Fixed Effect Model Summary
1.16 [1.11, 1.22]
0.5
0.75
1
1.25
1.5
1.75
Odds Ratio (95%CI)

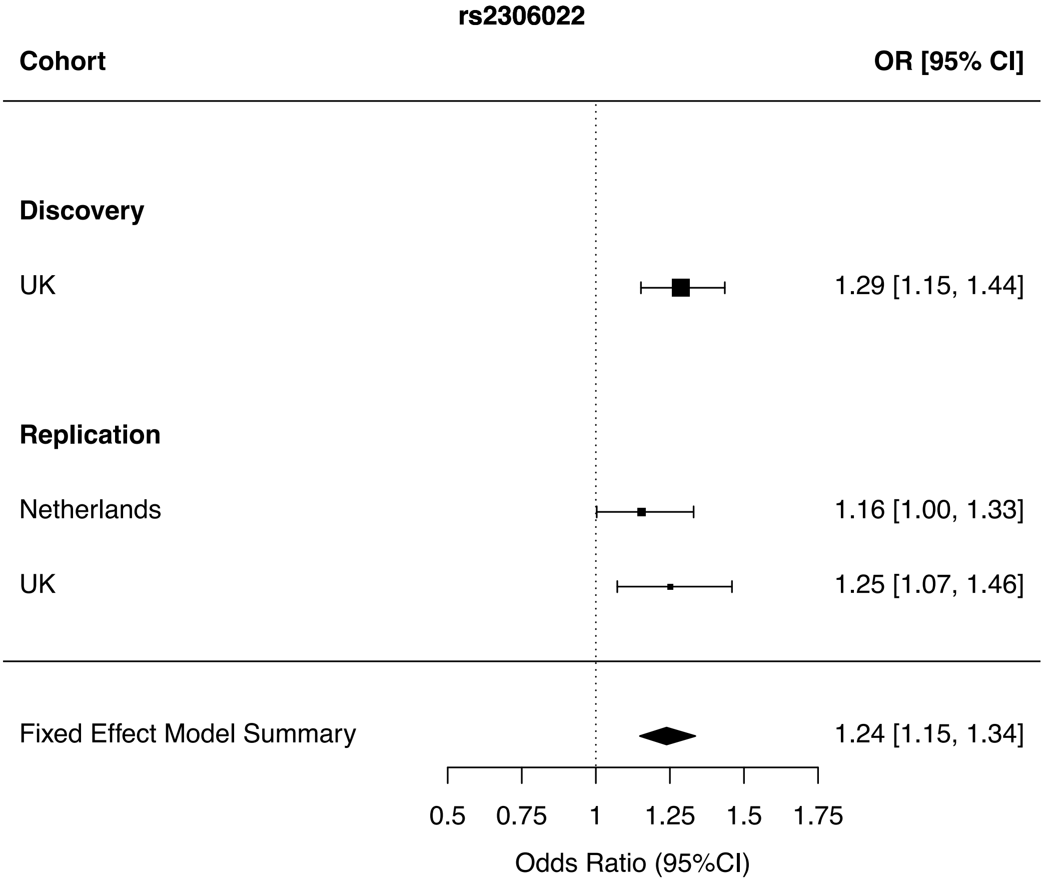

rs2306022
Cohort
OR [95% CI]
Discovery
UK
1.29 [1.15, 1.44]
Replication
Netherlands
1.16 [1.00, 1.33]
UK
1.25 [1.07, 1.46]
Fixed Effect Model Summary
1.24 [1.15, 1.34]
0.5
0.75
1
1.25
1.5
1.75
Odds Ratio (95%CI)

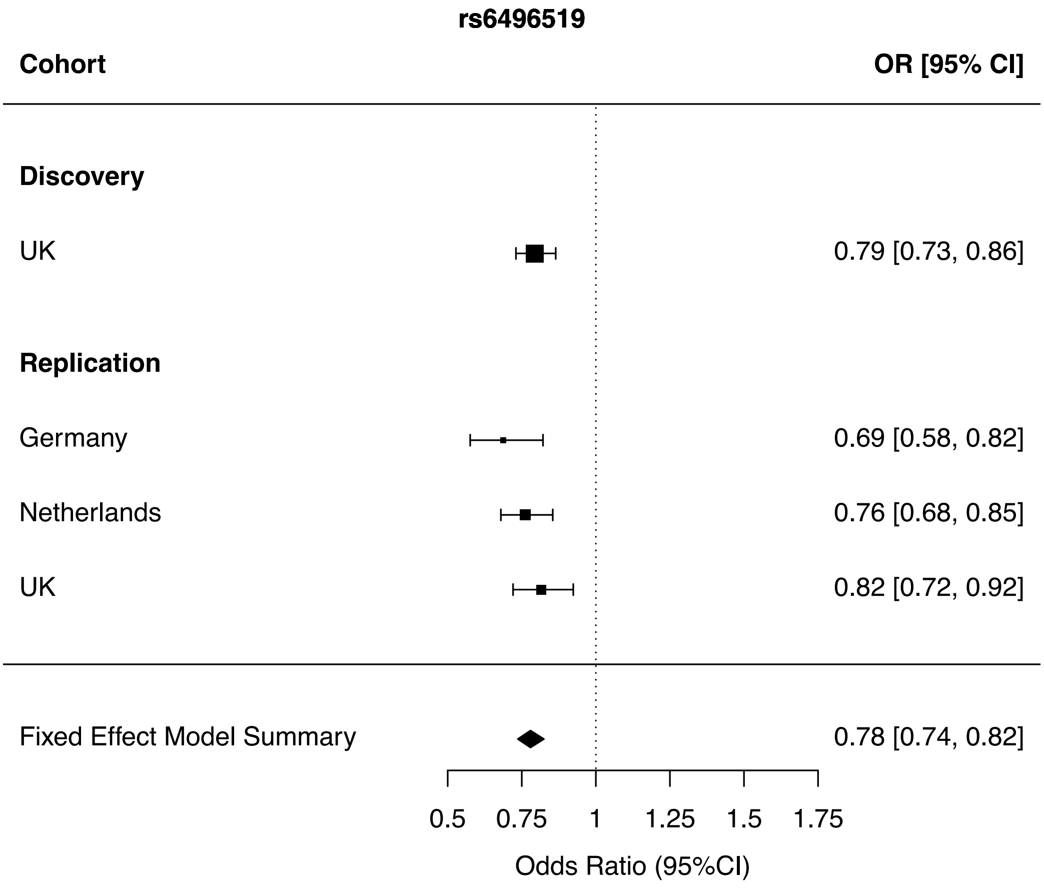
rs6496519
Cohort
OR [95% CI]
Discovery
UK
0.79 [0.73, 0.86]
Replication
Germany
0.69 [0.58, 0.82]
Netherlands
0.76 [0.68, 0.85]
UK
0.82 [0.72, 0.92]
Fixed Effect Model Summary
0.78 [0.74, 0.82]
0.5
0.75
1
1.25
1.5
1.75
Odds Ratio (95%CI)

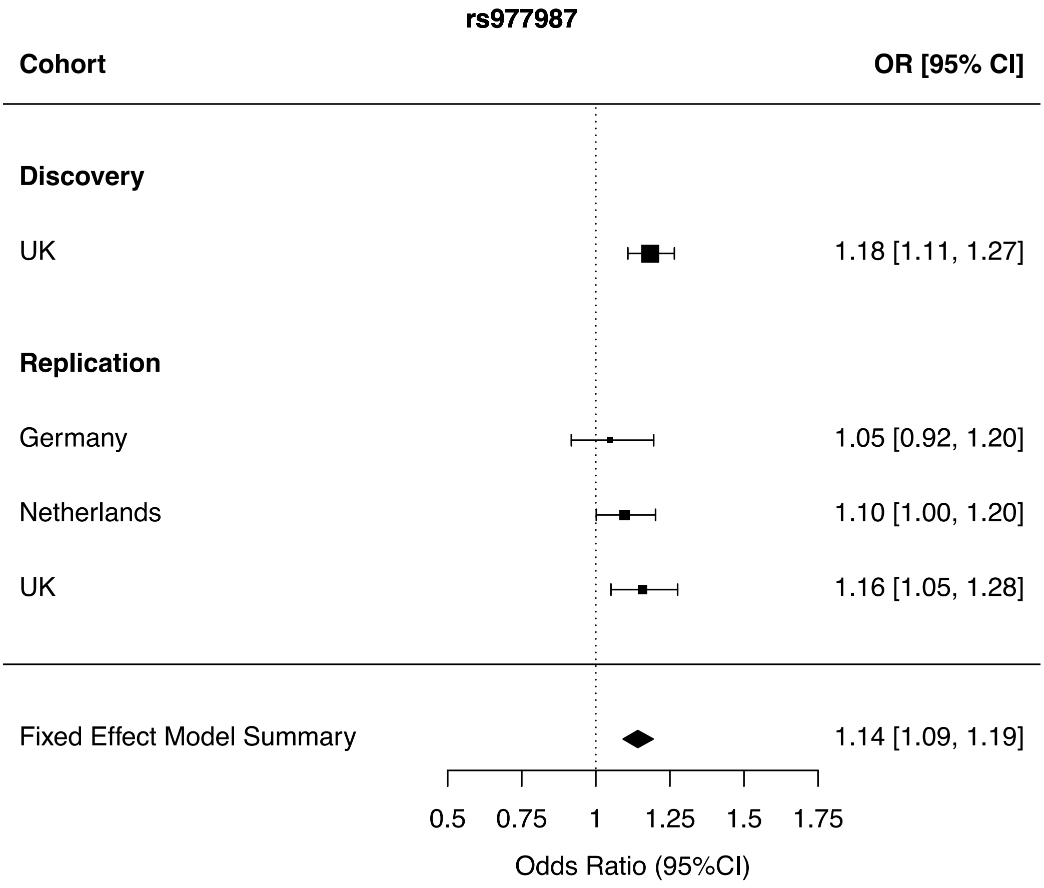
rs977987
Cohort
OR [95% CI]
Discovery
UK
1.18 [1.11, 1.27]
Replication
Germany
1.05 [0.92, 1.20]
Netherlands
1.10 [1.00, 1.20]
UK
1.16 [1.05, 1.28]
Fixed Effect Model Summary
1.14 [1.09, 1.19]
0.5
0.75
1
1.25
1.5
1.75
Odds Ratio (95%CI)

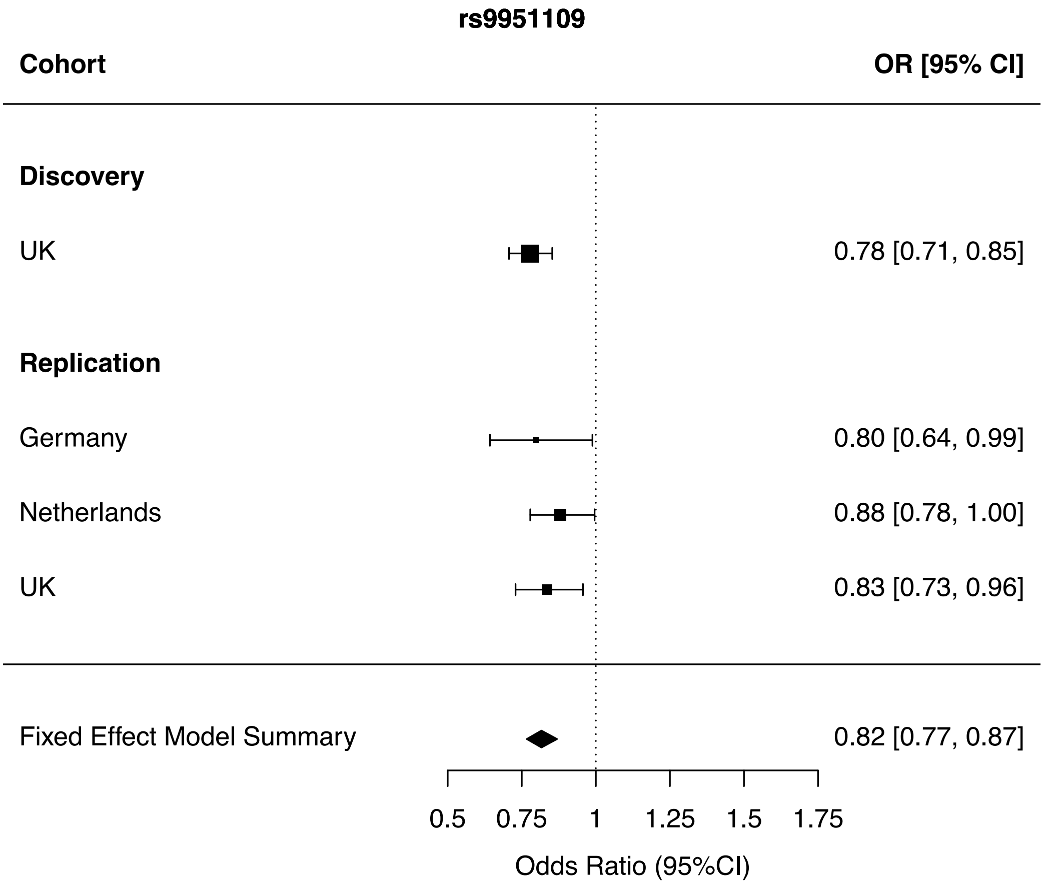
rs9951109
Cohort
OR [95% CI]
Discovery
UK
0.78 [0.71, 0.85]
Replication
Germany
0.80 [0.64, 0.99]
Netherlands
0.88 [0.78, 1.00]
UK
0.83 [0.73, 0.96]
Fixed Effect Model Summary
0.82 [0.77, 0.87]
0.5
0.75
1
1.25
1.5
1.75
Odds Ratio (95%CI)

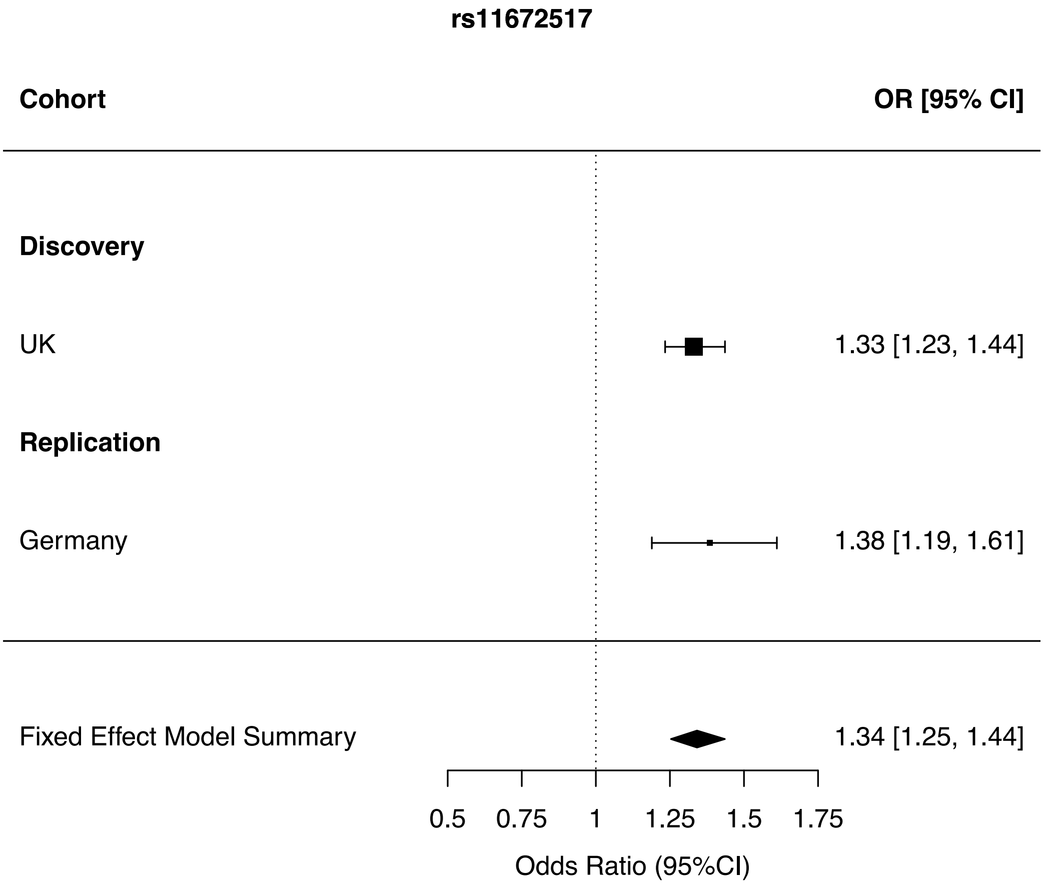
rs11672517
Cohort
OR [95% CI]
Discovery
UK
1.33 [1.23, 1.44]
Replication
Germany
1.38 [1.19, 1.61]
Fixed Effect Model Summary
1.34 [1.25, 1.44]
0.5
0.75
1
1.25
1.5
1.75
Odds Ratio (95%CI)

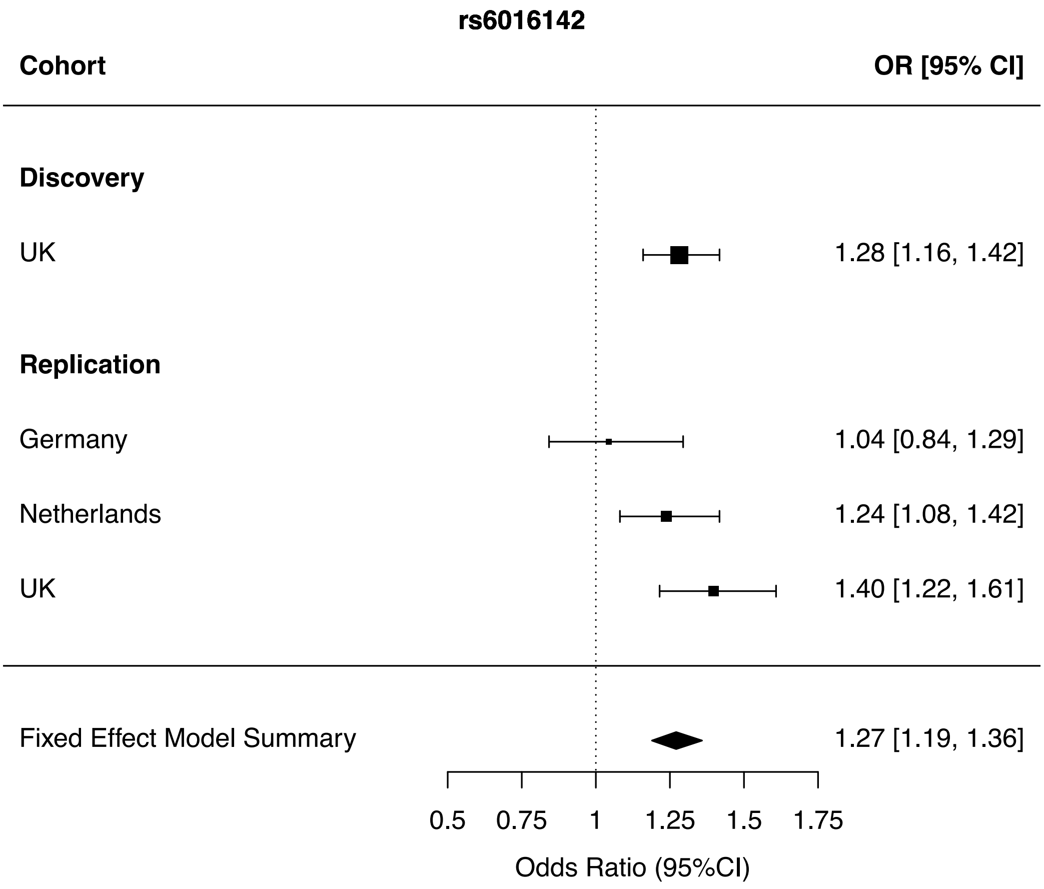
rs6016142
Cohort
OR [95% CI]
Discovery
UK
1.28 [1.16, 1.42]
Replication
Germany
1.04 [0.84, 1.29]
Netherlands
1.24 [1.08, 1.42]
UK
1.40 [1.22, 1.61]
Fixed Effect Model Summary
1.27 [1.19, 1.36]
0.5
0.75
1
1.25
1.5
1.75
Odds Ratio (95%CI)

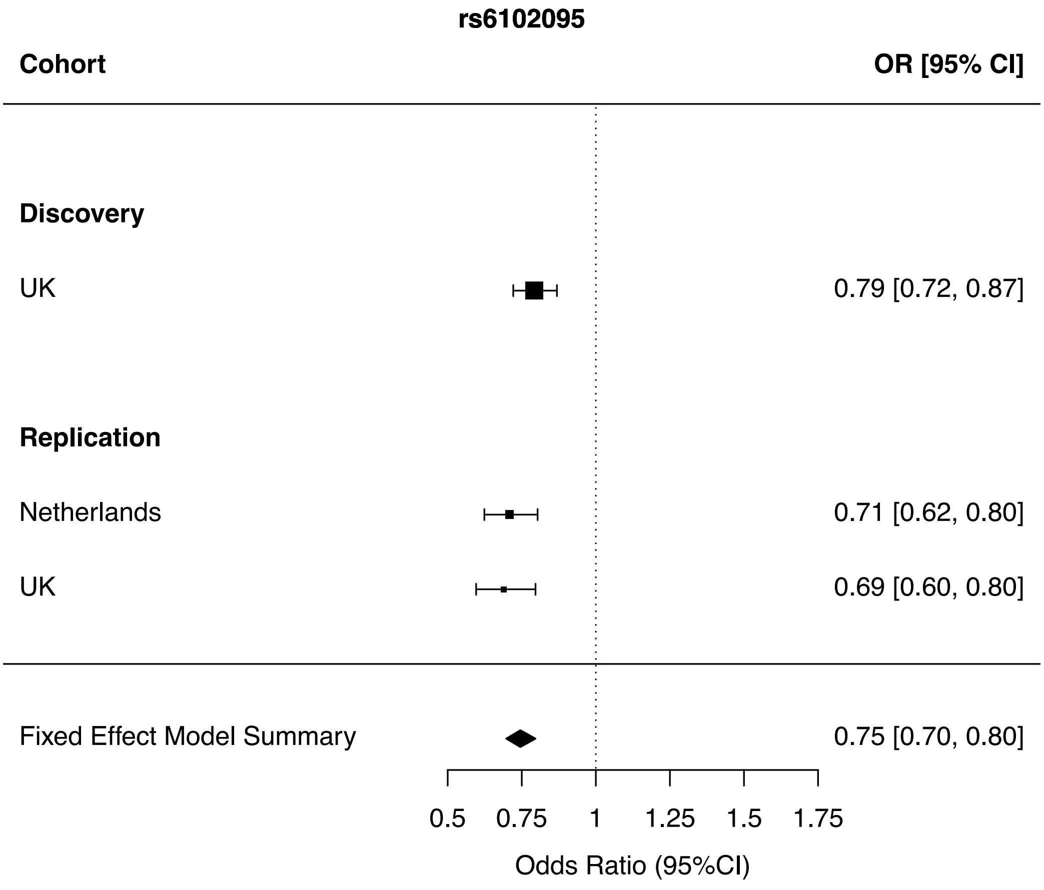
rs6102095
Cohort
OR [95% CI]
Discovery
UK
0.79 [0.72, 0.87]
Replication
Netherlands
0.71 [0.62, 0.80]
UK
0.69 [0.60, 0.80]
Fixed Effect Model Summary
0.75 [0.70, 0.80]
0.5
0.75
1
1.25
1.5
1.75
Odds Ratio (95%CI)

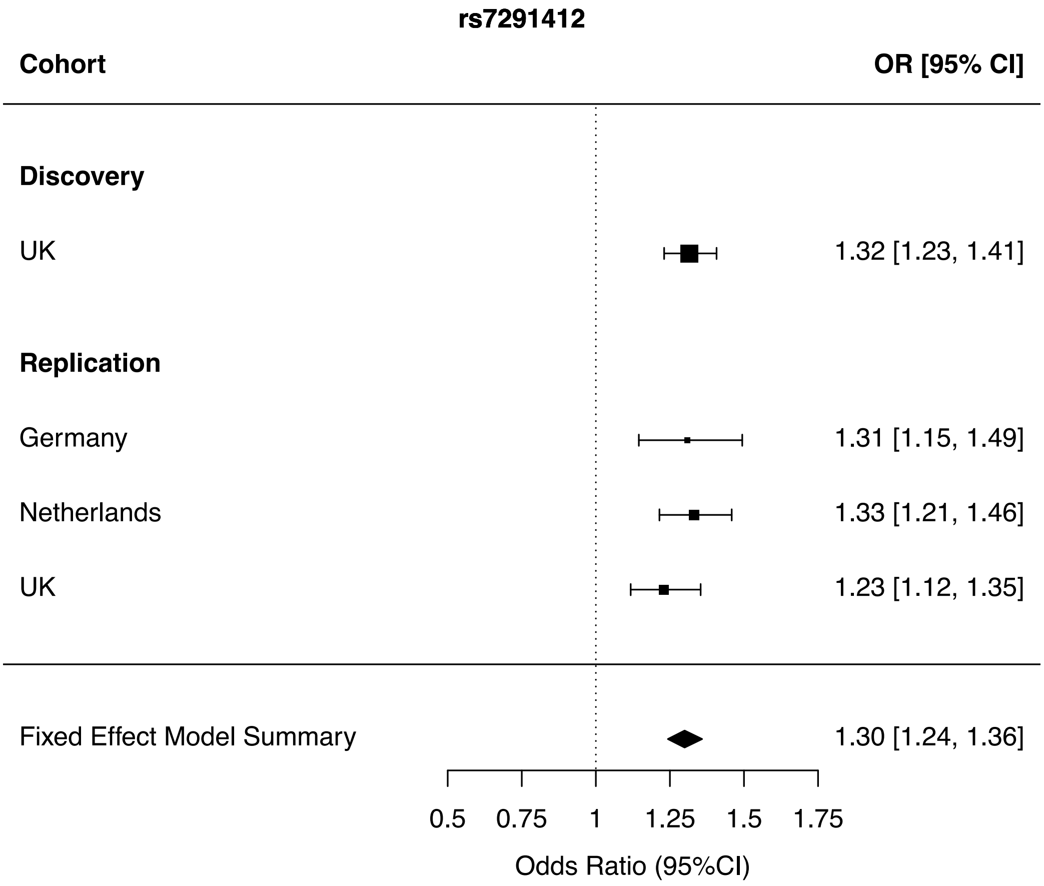

rs7291412
Cohort
OR [95% CI]
Discovery
UK
1.32 [1.23, 1.41]
Replication
Germany
1.31 [1.15, 1.49]
Netherlands
1.33 [1.21, 1.46]
UK
1.23 [1.12, 1.35]
Fixed Effect Model Summary
1.30 [1.24, 1.36]
0.5
0.75
1
1.25
1.5
1.75
Odds Ratio (95%CI)

Figure S6. Annotated regional association plots for all 26 variants significantly associated with Dupuytren's disease ($p \leq 5x10^{-8}$) after meta-analysis of discovery and replication cohorts, plotted with LocusZoom [1]. Recombination rate was calculated within the software using data from HapMap genotypes. In the lower panel of each figure, genes within 500kb of the index SNP are shown. The position on each chromosome is shown in relation to Human Genome build hg19.

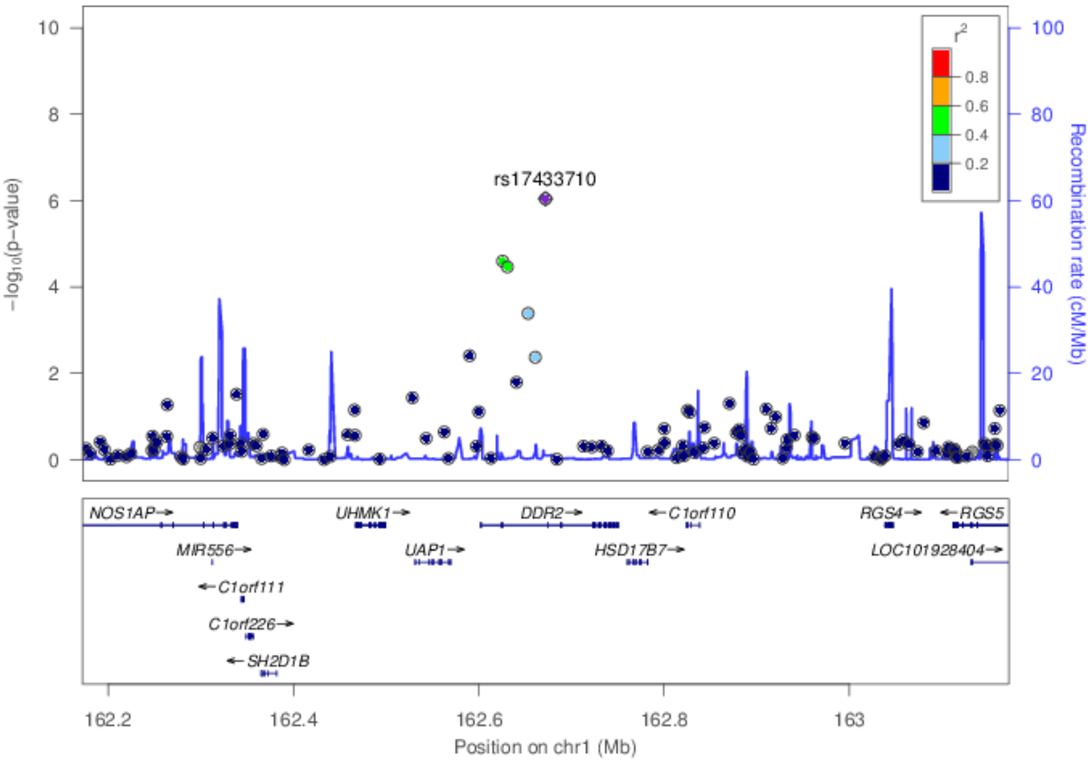

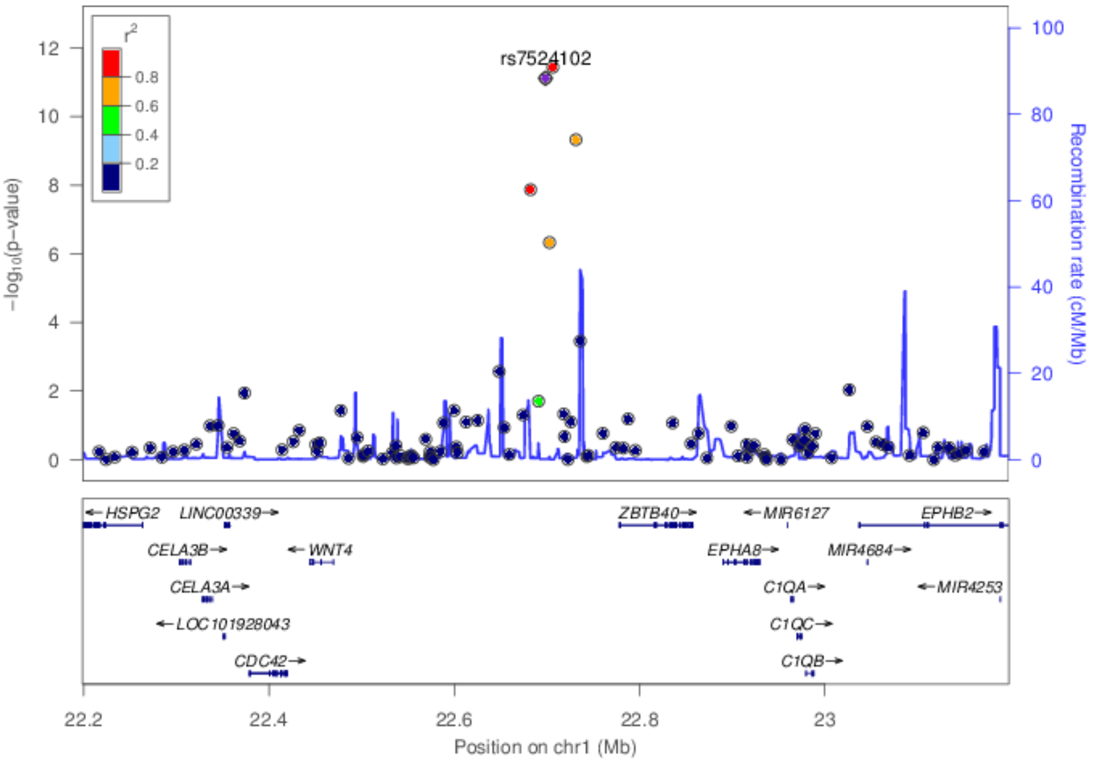
rs7524102
r2
0.8
0.6
0.4
0.2
−log10(p−value)
Recombination rate (cM/Mb)
HSPG2
LINC00339
CELA3B
WNT4
CELA3A
LOC101928043
CDC42
ZBTB40
MIR6127
EPHB2
EPHA8
MIR4684
C1QA
MIR4253
C1QC
C1QB
Position on chr1 (Mb)

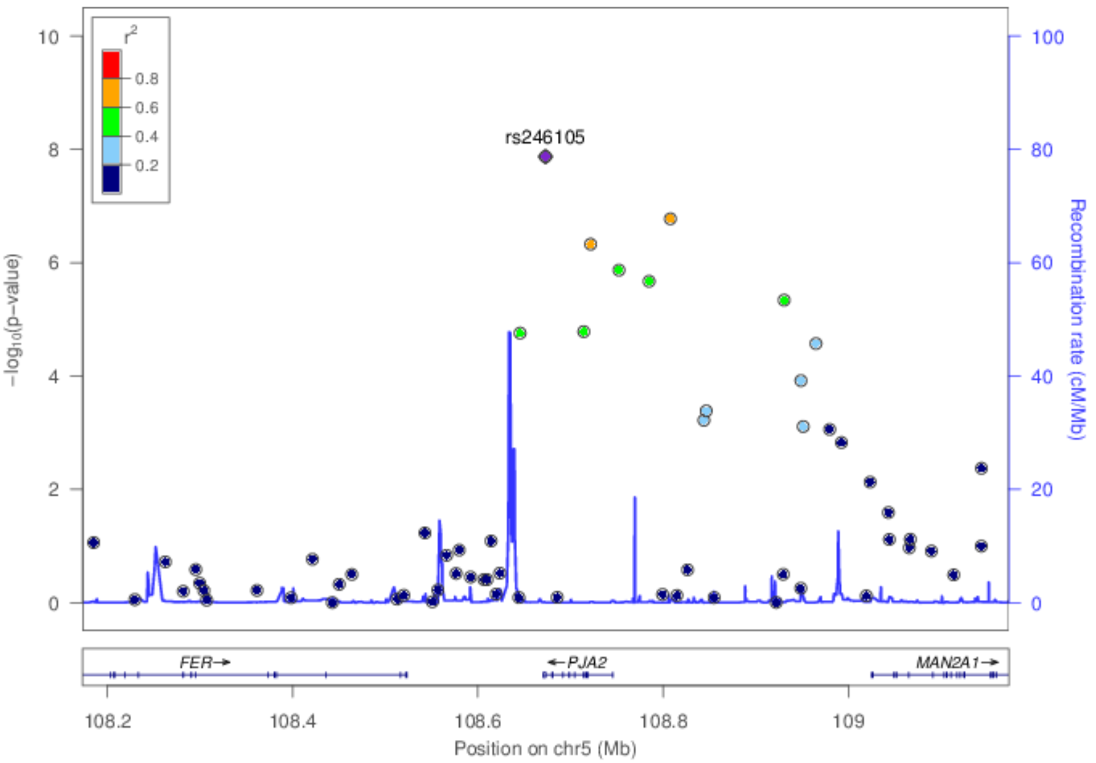
rs246105
r2
0.8
0.6
0.4
0.2
−log10(p−value)
Recombination rate (cM/Mb)
FER
PJA2
MAN2A1
Position on chr5 (Mb)

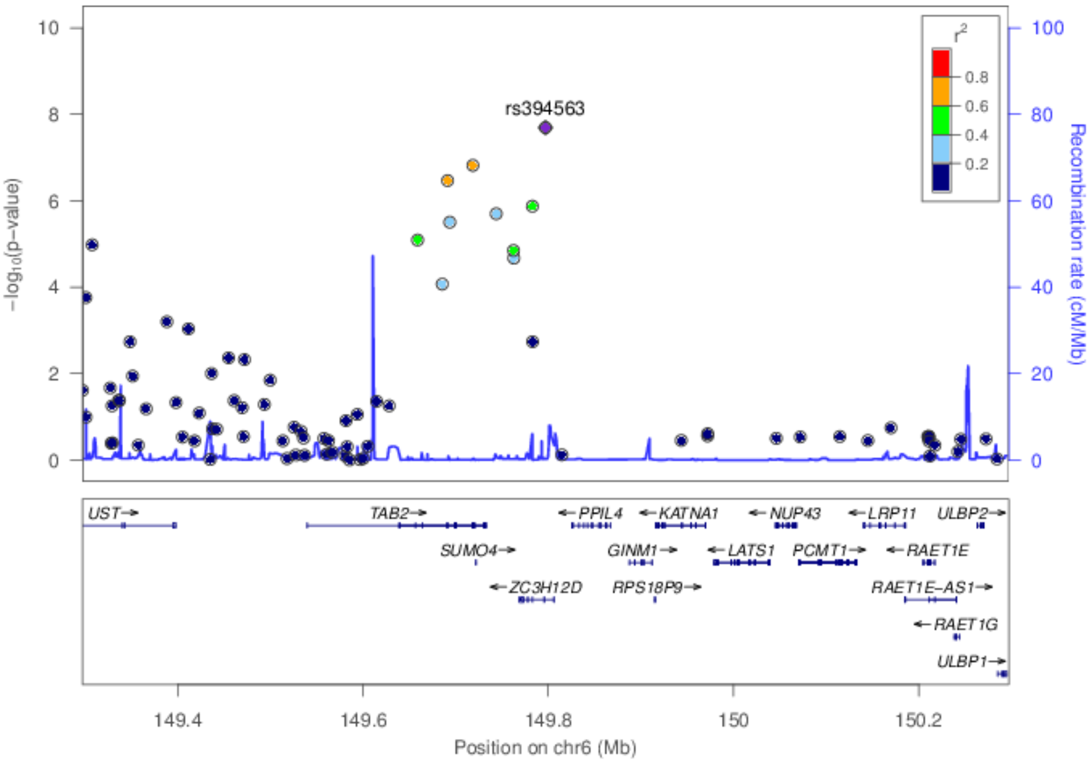

rs394563
r²
0.8
0.6
0.4
0.2
−log10(p−value)
Recombination rate (cM/Mb)
UST
TAB2
SUMO4
ZC3H12D
PPIL4
GINM1
RPS18P9
KATNA1
LATS1
NUP43
PCMT1
LRP11
RAET1E
RAET1E-AS1
RAET1G
ULBP2
ULBP1
149.4
149.6
149.8
150
150.2
Position on chr6 (Mb)

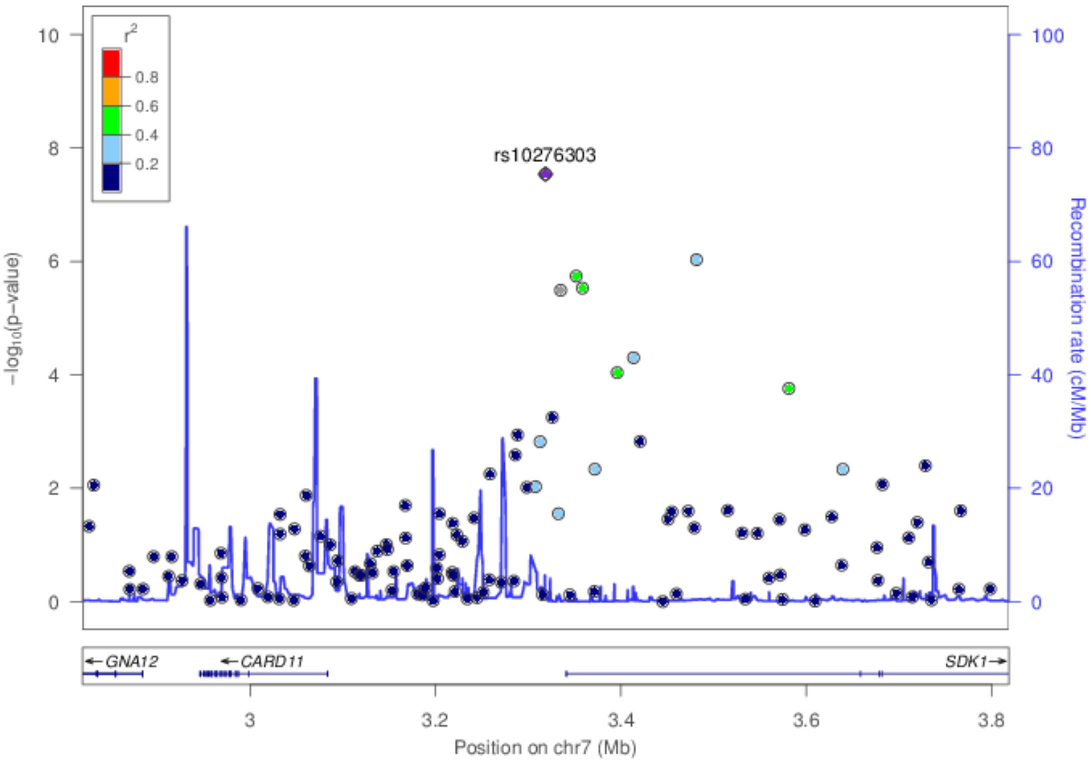

rs10276303
r²
0.8
0.6
0.4
0.2
−log10(p−value)
Recombination rate (cM/Mb)
GNA12
CARD11
SDK1
3
3.2
3.4
3.6
3.8
Position on chr7 (Mb)

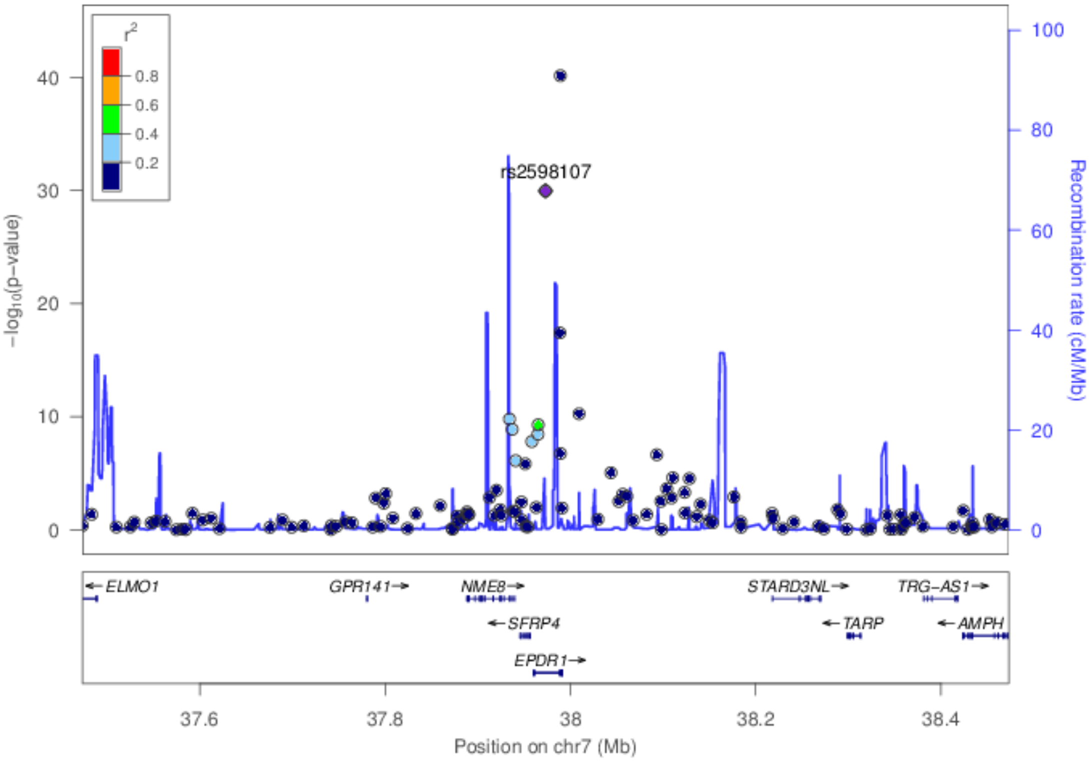

r2
0.8
0.6
0.4
0.2
rs2598107
−log10(p−value)
Recombination rate (cM/Mb)
ELMO1
GPR141
NME8
SFRP4
EPDR1
STARD3NL
TARP
TRG−AS1
AMPH
Position on chr7 (Mb)

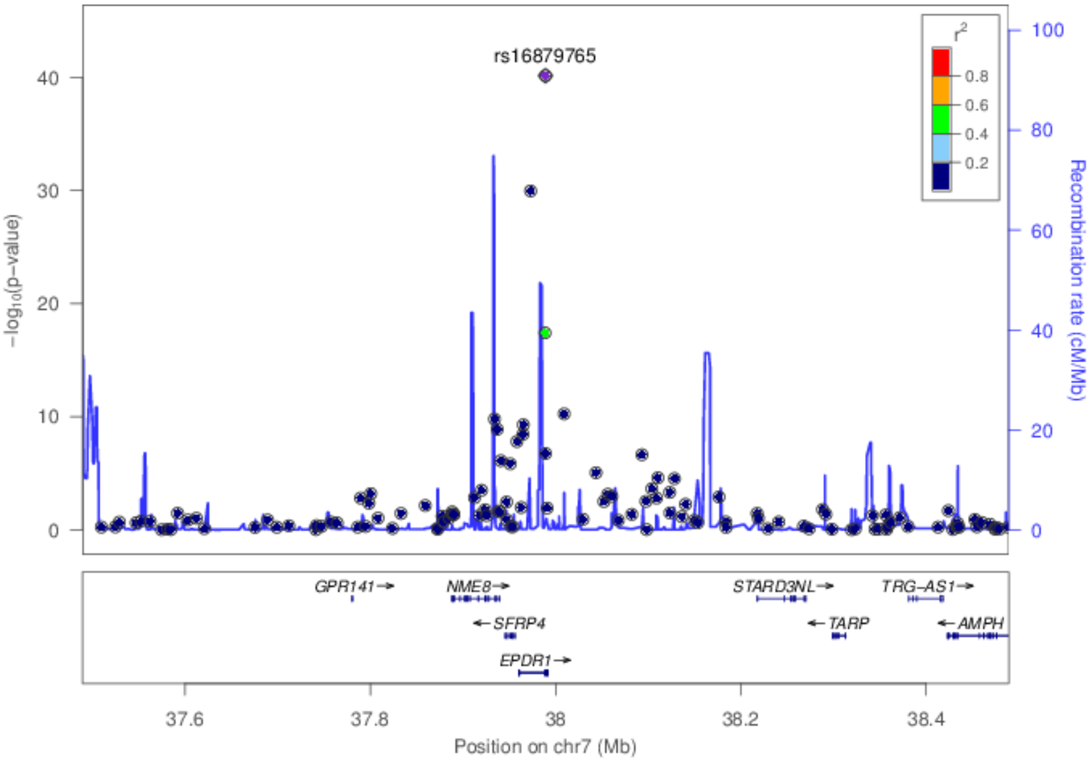

r2
0.8
0.6
0.4
0.2
rs16879765
−log10(p−value)
Recombination rate (cM/Mb)
GPR141
NME8
SFRP4
EPDR1
STARD3NL
TARP
TRG−AS1
AMPH
Position on chr7 (Mb)

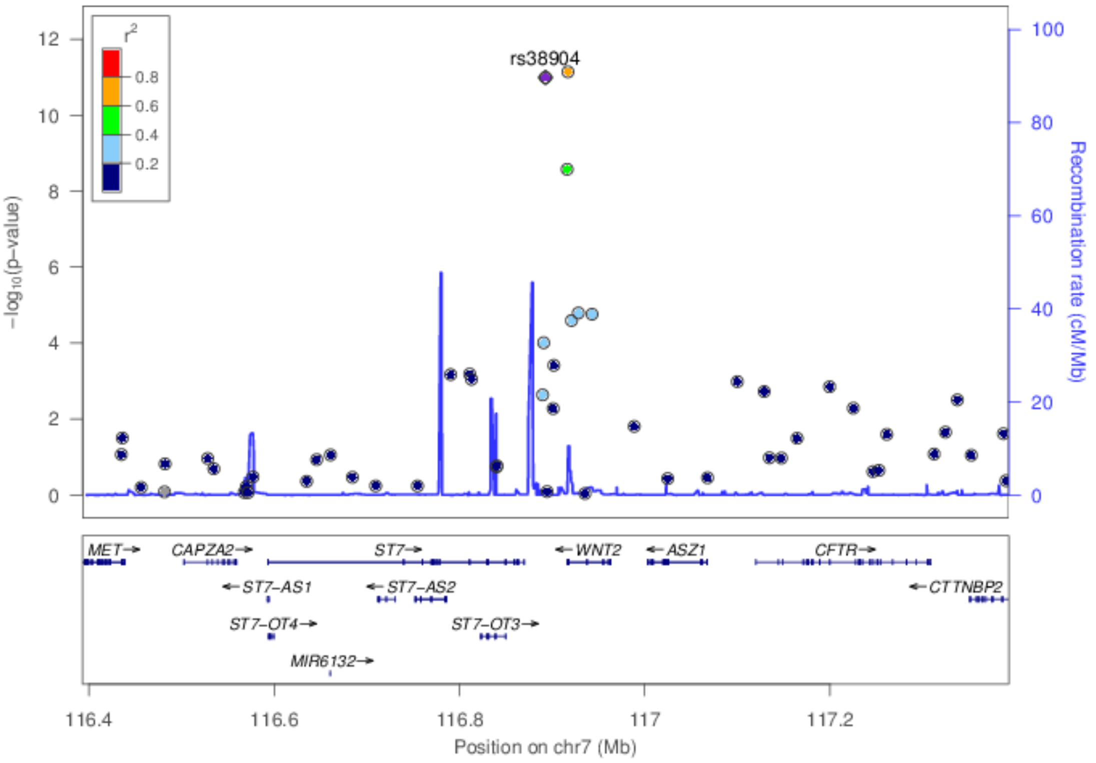

rs38904
r2
0.8
0.6
0.4
0.2
−log10(p−value)
Recombination rate (cM/Mb)
MET
CAPZA2
ST7
WNT2
ASZ1
CFTR
ST7−AS1
ST7−AS2
CTTNBP2
ST7−OT4
ST7−OT3
MIR6132
Position on chr7 (Mb)

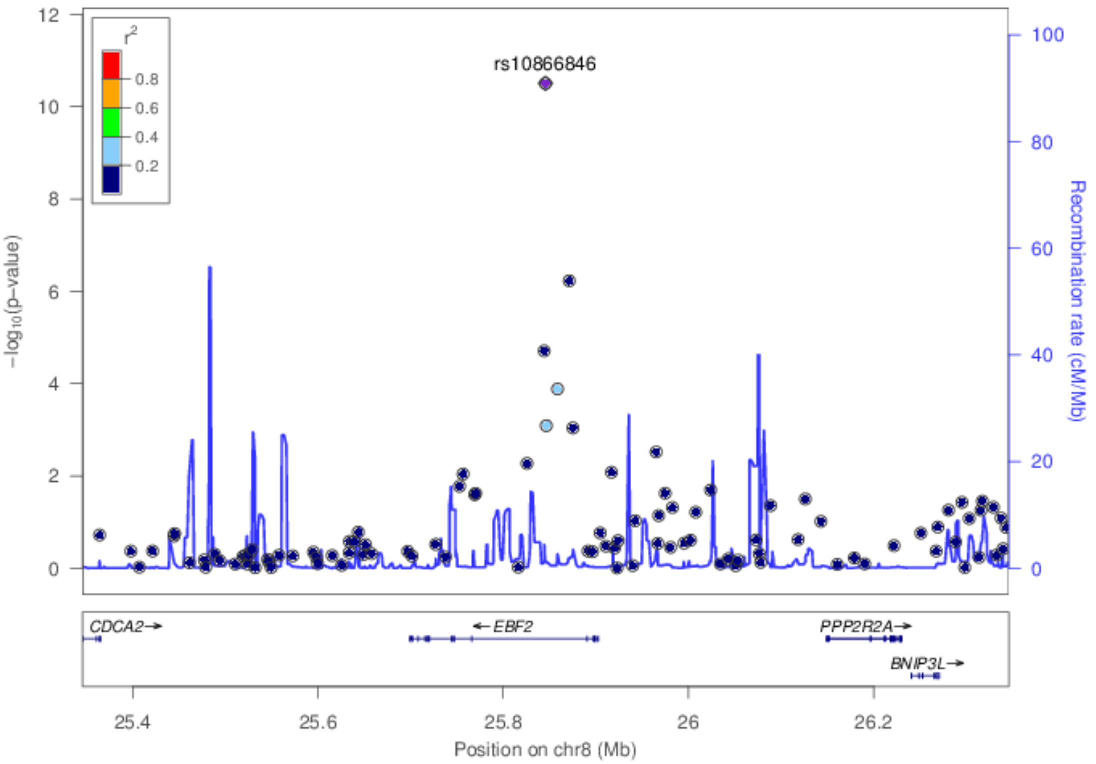

rs10866846
r2
0.8
0.6
0.4
0.2
−log10(p−value)
Recombination rate (cM/Mb)
CDCA2
EBF2
PPP2R2A
BNIP3L
Position on chr8 (Mb)

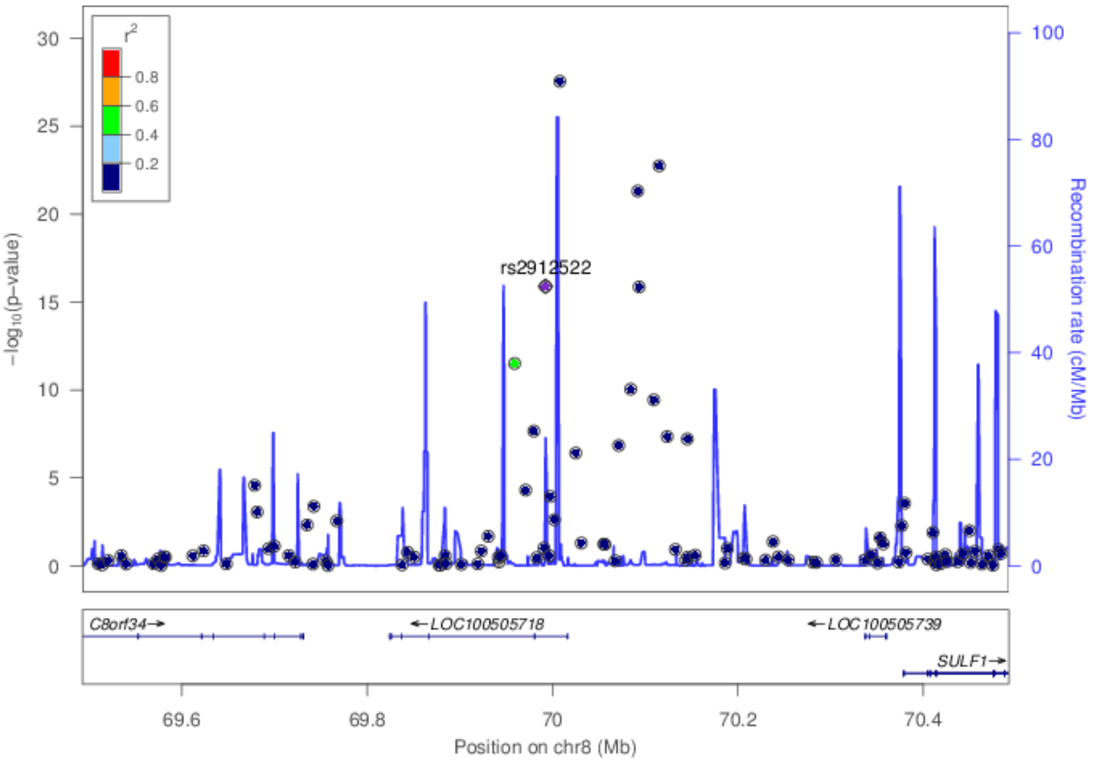

r2
0.8
0.6
0.4
0.2
rs2912522
−log10(p−value)
Recombination rate (cM/Mb)
C8orf34
LOC100505718
LOC100505739
SULF1
69.6
69.8
70
70.2
70.4
Position on chr8 (Mb)

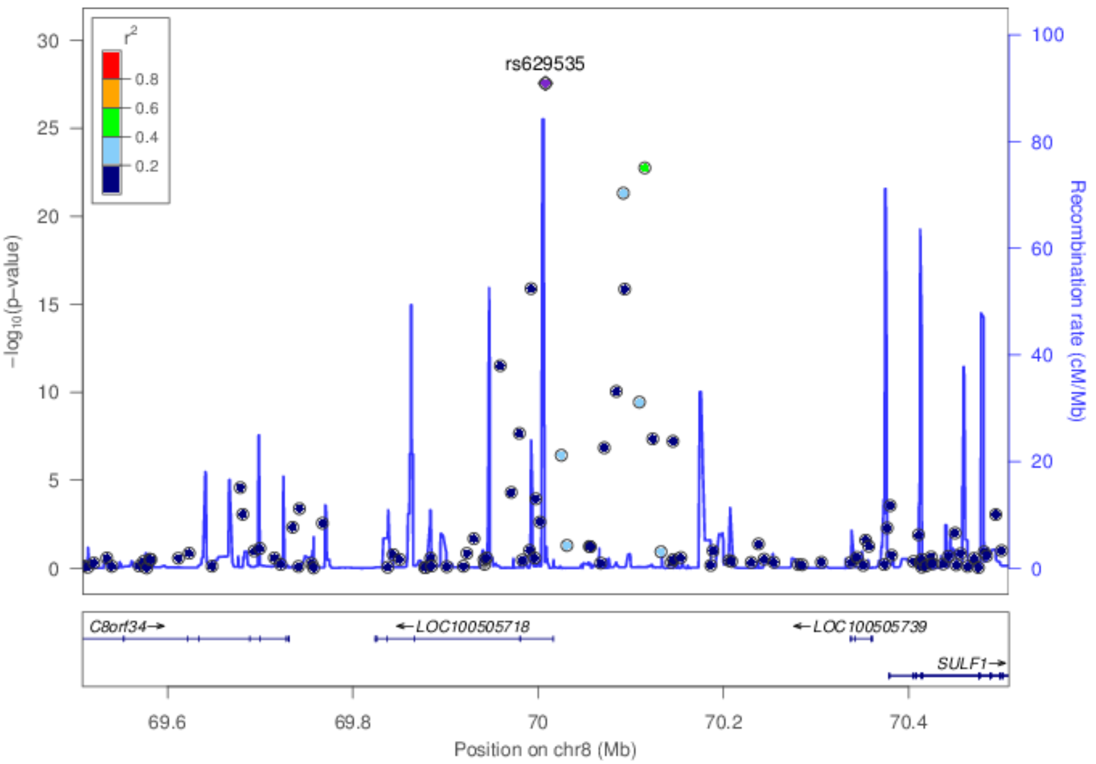

r2
0.8
0.6
0.4
0.2
rs629535
−log10(p−value)
Recombination rate (cM/Mb)
C8orf34
LOC100505718
LOC100505739
SULF1
69.6
69.8
70
70.2
70.4
Position on chr8 (Mb)

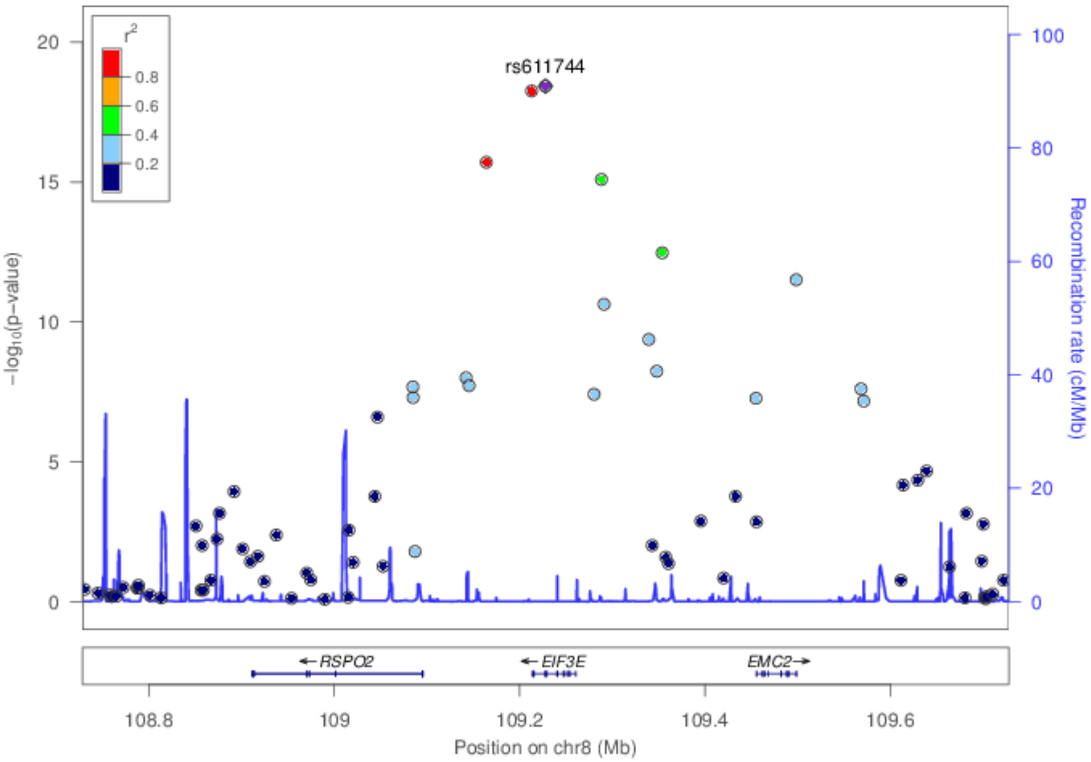
rs611744
r²
0.8
0.6
0.4
0.2
−log₁₀(p−value)
Recombination rate (cM/Mb)
RSPO2
EIF3E
EMC2
108.8
109
109.2
109.4
109.6
Position on chr8 (Mb)

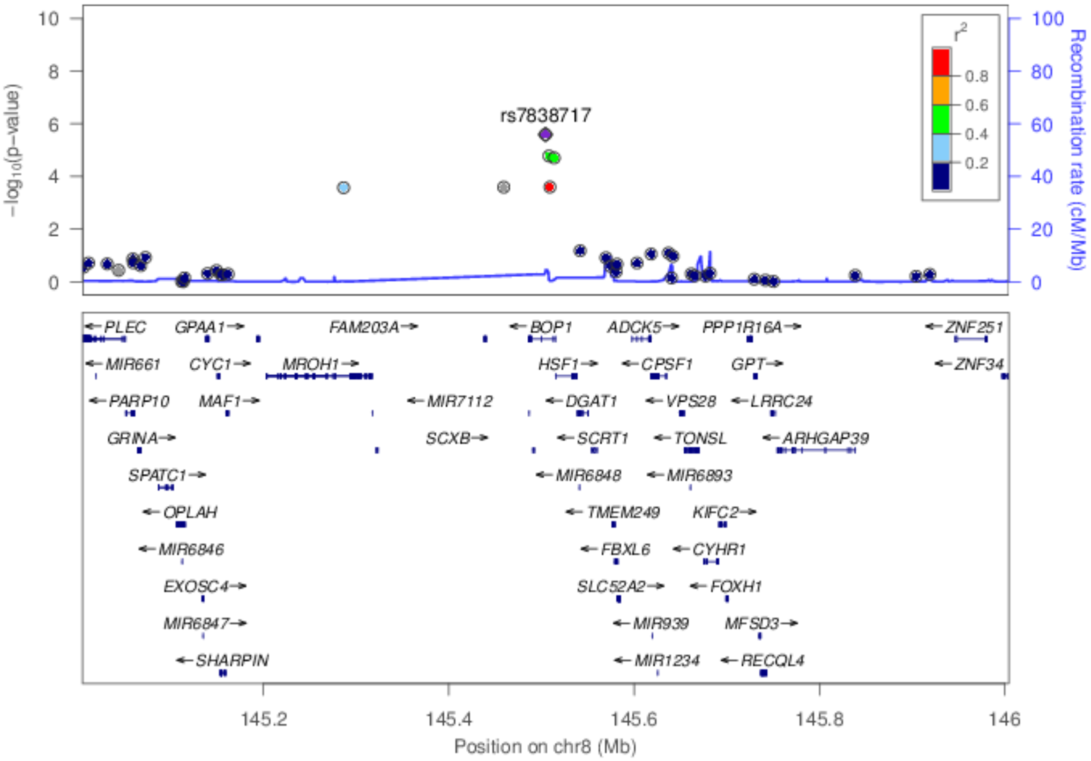
rs7838717
r²
0.8
0.6
0.4
0.2
−log₁₀(p−value)
Recombination rate (cM/Mb)
PLEC
GPAA1
FAM203A
BOP1
ADCK5
PPP1R16A
ZNF251
MIR661
CYC1
MROH1
HSF1
CPSF1
GPT
ZNF34
PARP10
MAF1
MIR7112
DGAT1
VPS28
LRRC24
GRINA
SCXB
SCRT1
TONSL
ARHGAP39
SPATC1
MIR6848
MIR6893
OPLAH
TMEM249
KIFC2
MIR6846
FBXL6
CYHR1
EXOSC4
SLC52A2
FOXH1
MIR6847
MIR939
MFSD3
SHARPIN
MIR1234
RECQL4
145.2
145.4
145.6
145.8
146
Position on chr8 (Mb)

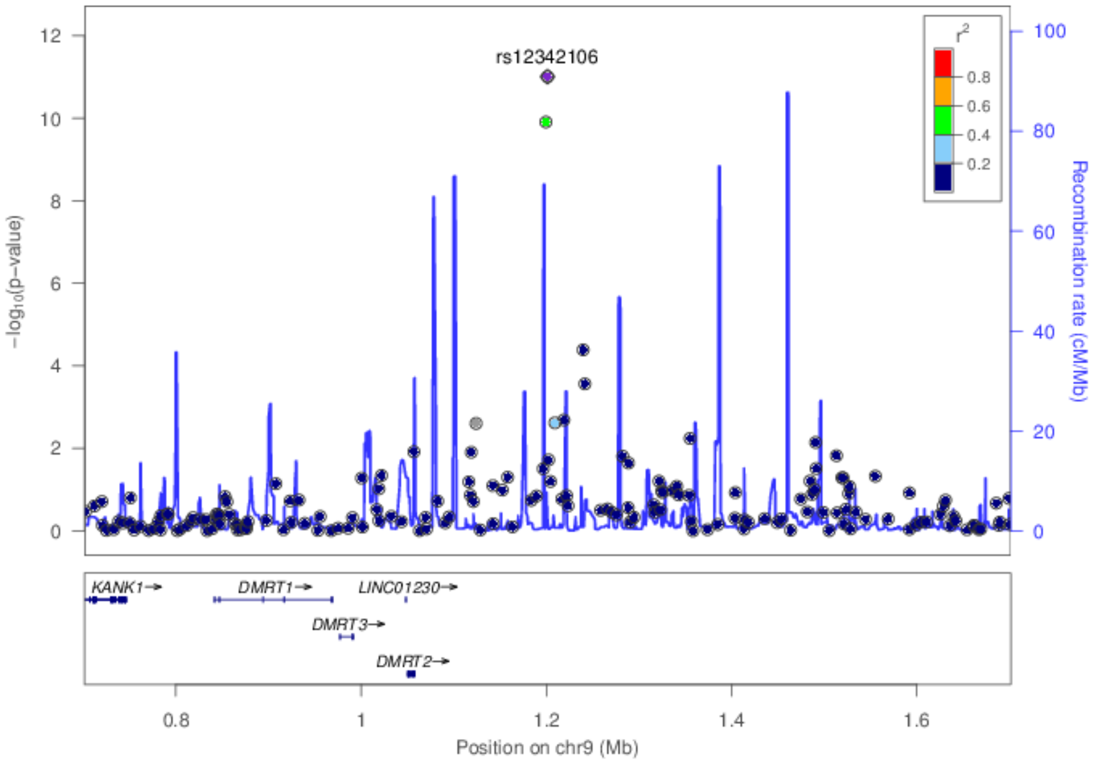

rs12342106
r2
0.8
0.6
0.4
0.2
−log10(p−value)
Recombination rate (cM/Mb)
KANK1→
DMRT1→
LINC01230→
DMRT3→
DMRT2→
Position on chr9 (Mb)

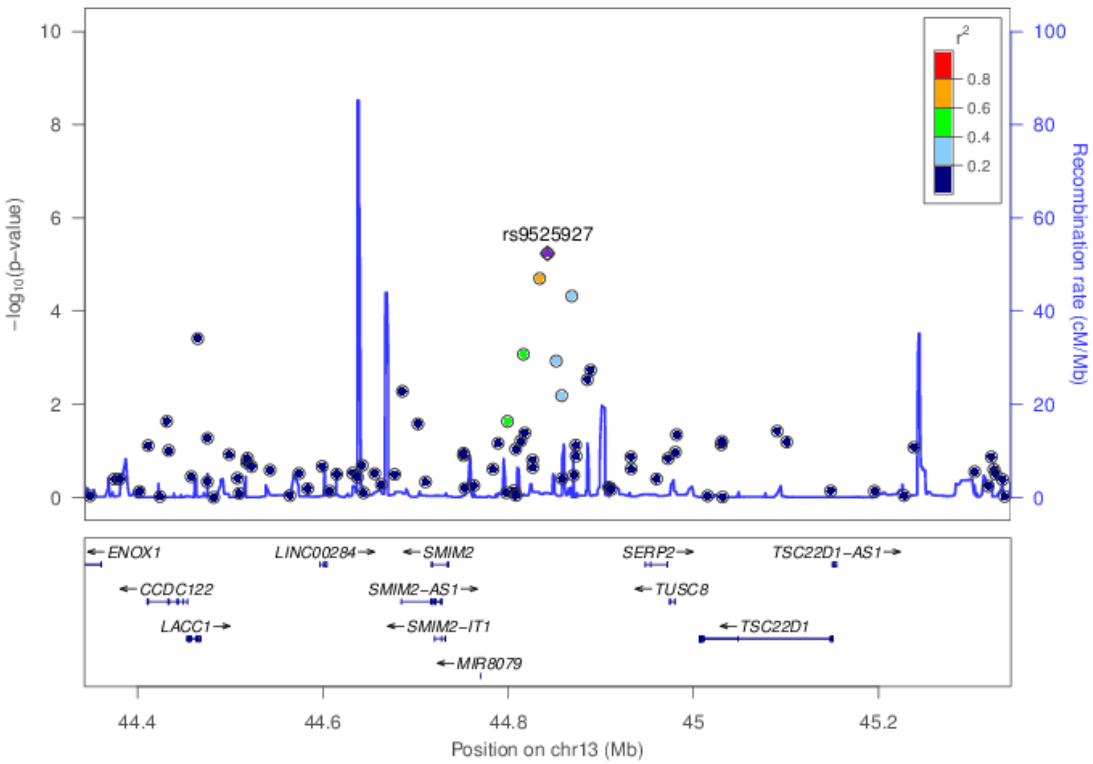

rs9525927
r2
0.8
0.6
0.4
0.2
−log10(p−value)
Recombination rate (cM/Mb)
←ENOX1
LINC00284→
←SMIM2
SERP2→
TSC22D1−AS1→
←CCDC122
SMIM2−AS1→
←TUSC8
LACC1→
←SMIM2−IT1
←TSC22D1
←MIR8079
Position on chr13 (Mb)

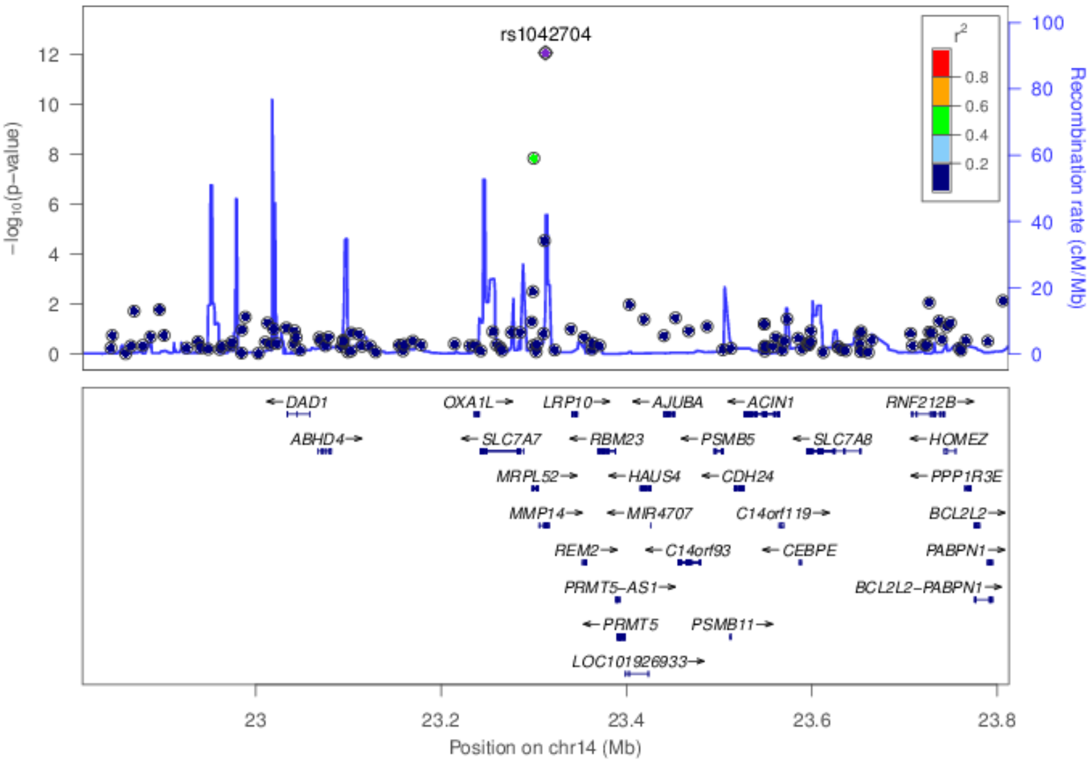

rs1042704
r2
0.8
0.6
0.4
0.2
−log10(p−value)
Recombination rate (cM/Mb)
DAD1
ABHD4
OXA1L
SLC7A7
MRPL52
MMP14
LRP10
RBM23
REM2
PRMT5−AS1
PRMT5
LOC101926933
AJUBA
HAUS4
MIR4707
C14orf93
PSMB5
CDH24
PSMB11
ACIN1
C14orf119
CEBPE
SLC7A8
RNF212B
HOMEZ
PPP1R3E
BCL2L2
PABPN1
BCL2L2−PABPN1
Position on chr14 (Mb)

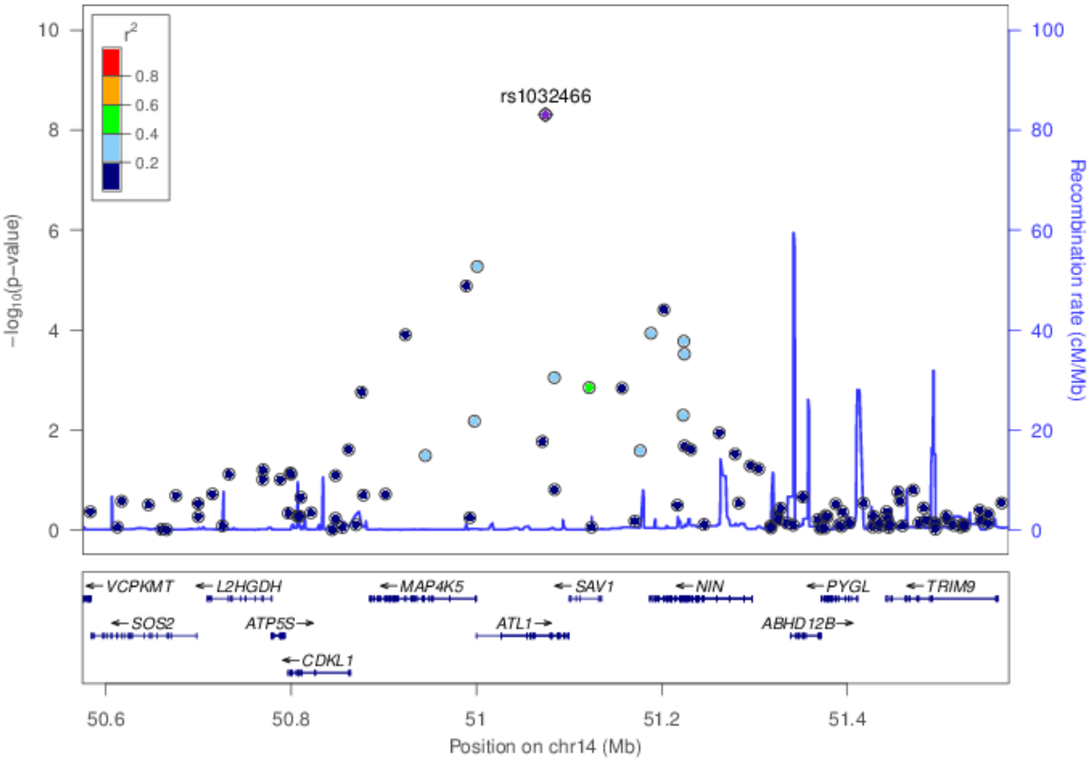

rs1032466
r2
0.8
0.6
0.4
0.2
−log10(p−value)
Recombination rate (cM/Mb)
VCPKMT
SOS2
L2HGDH
ATP5S
CDKL1
MAP4K5
ATL1
SAV1
NIN
PYGL
ABHD12B
TRIM9
Position on chr14 (Mb)

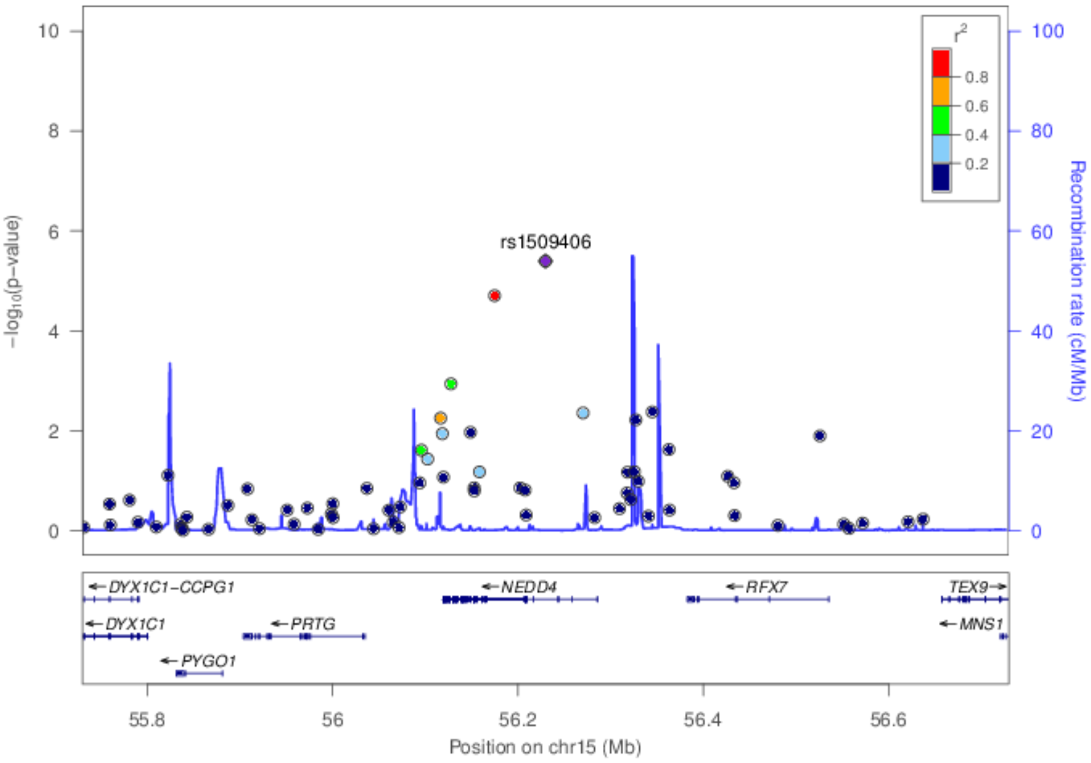
rs1509406
r2
0.8
0.6
0.4
0.2
−log10(p−value)
Recombination rate (cM/Mb)
DYX1C1−CCPG1
DYX1C1
PYGO1
PRTG
NEDD4
RFX7
TEX9
MNS1
Position on chr15 (Mb)

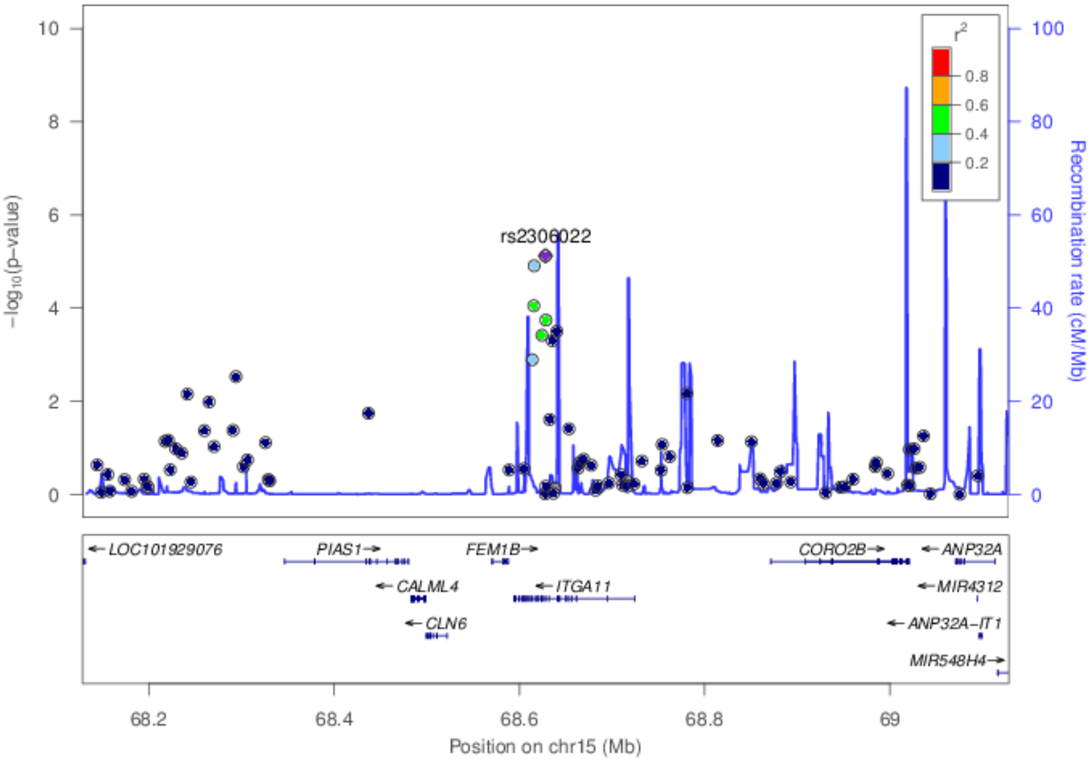
rs2306022
r2
0.8
0.6
0.4
0.2
−log10(p−value)
Recombination rate (cM/Mb)
LOC101929076
PIAS1
CALML4
CLN6
FEM1B
ITGA11
CORO2B
ANP32A
MIR4312
ANP32A−IT1
MIR548H4
Position on chr15 (Mb)

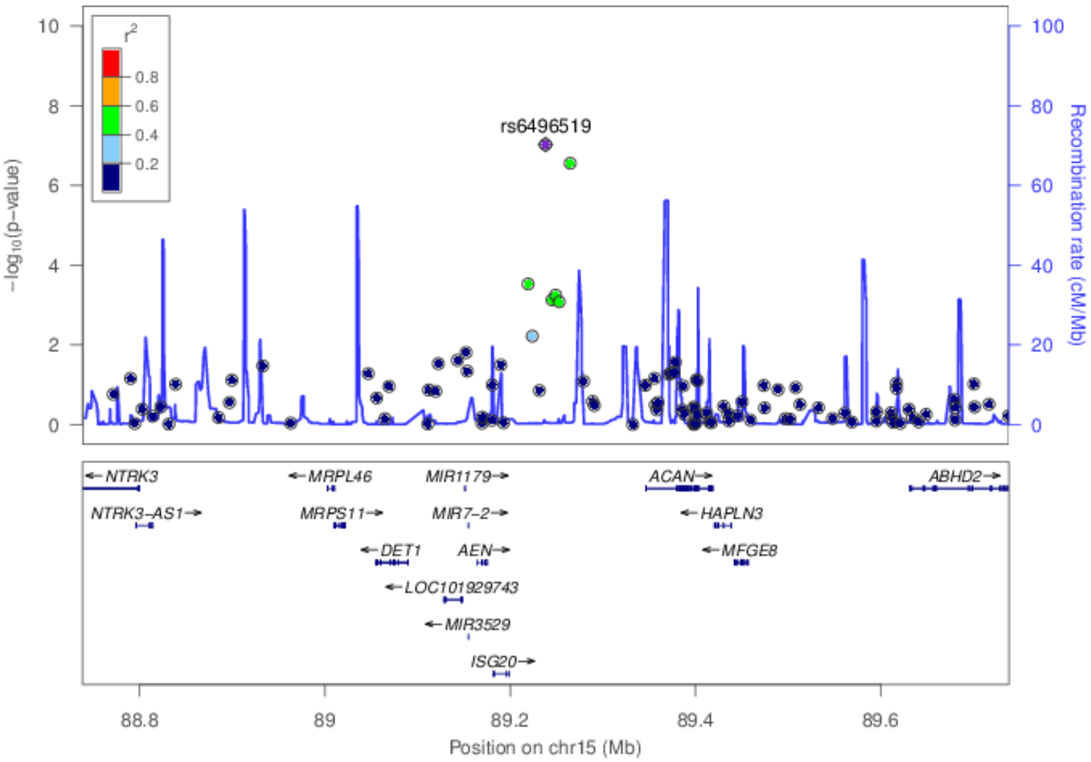

rs6496519
r2
0.8
0.6
0.4
0.2
−log10(p−value)
Recombination rate (cM/Mb)
NTRK3
NTRK3−AS1
MRPL46
MRPS11
DET1
LOC101929743
MIR1179
MIR7−2
AEN
MIR3529
ISG20
ACAN
HAPLN3
MFGE8
ABHD2
Position on chr15 (Mb)

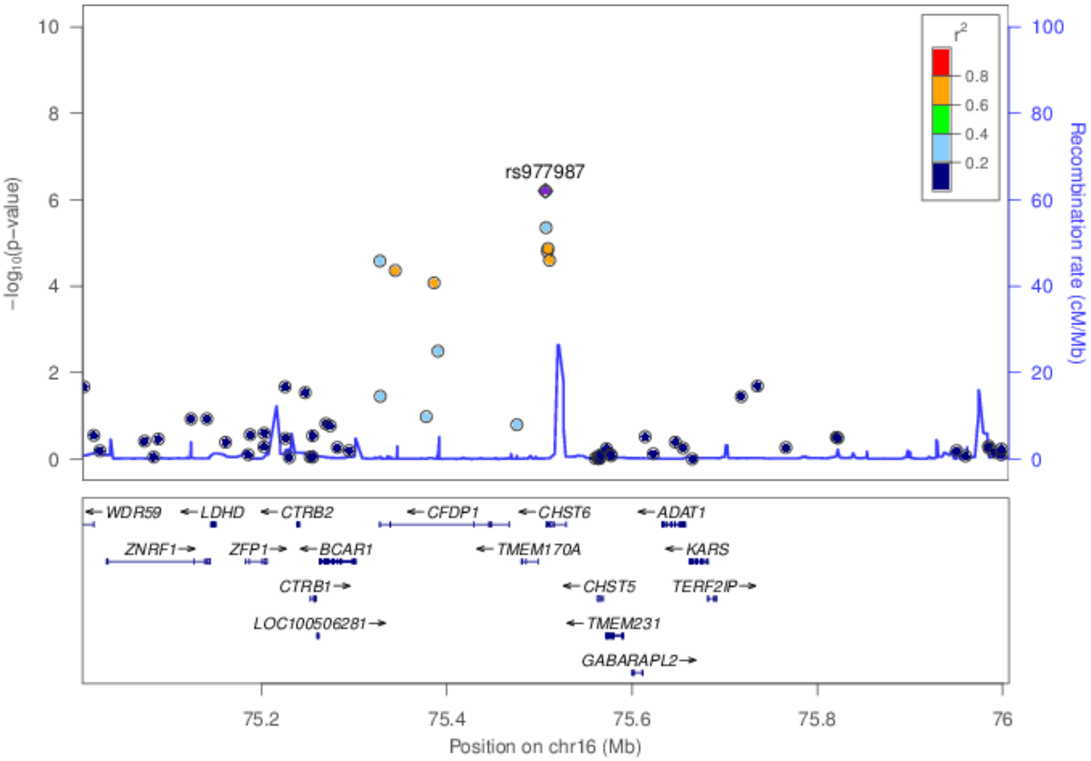

rs977987
r2
0.8
0.6
0.4
0.2
−log10(p−value)
Recombination rate (cM/Mb)
WDR59
LDHD
CTRB2
CFDP1
CHST6
ADAT1
ZNRF1
ZFP1
BCAR1
TMEM170A
KARS
CTRB1
CHST5
TERF2IP
LOC100506281
TMEM231
GABARAPL2
Position on chr16 (Mb)

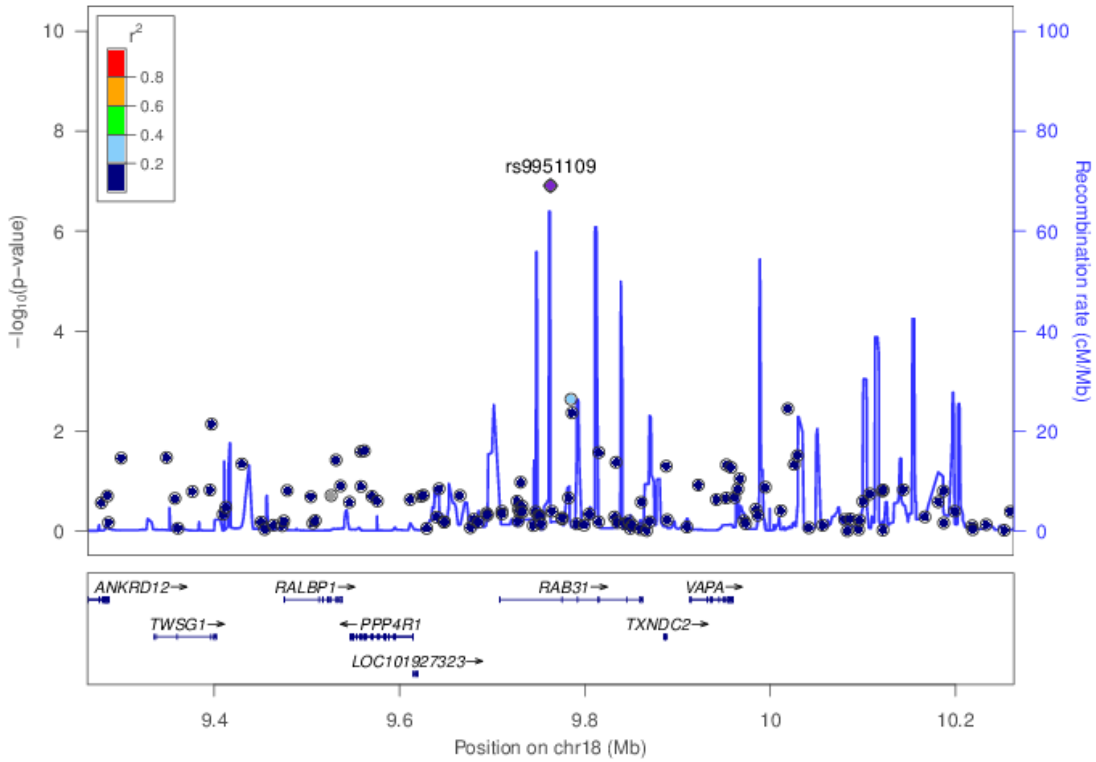
rs9951109
r²
0.8
0.6
0.4
0.2
−log10(p−value)
Recombination rate (cM/Mb)
ANKRD12
TWSG1
RALBP1
PPP4R1
LOC101927323
RAB31
TXNDC2
VAPA
9.4
9.6
9.8
10
10.2
Position on chr18 (Mb)

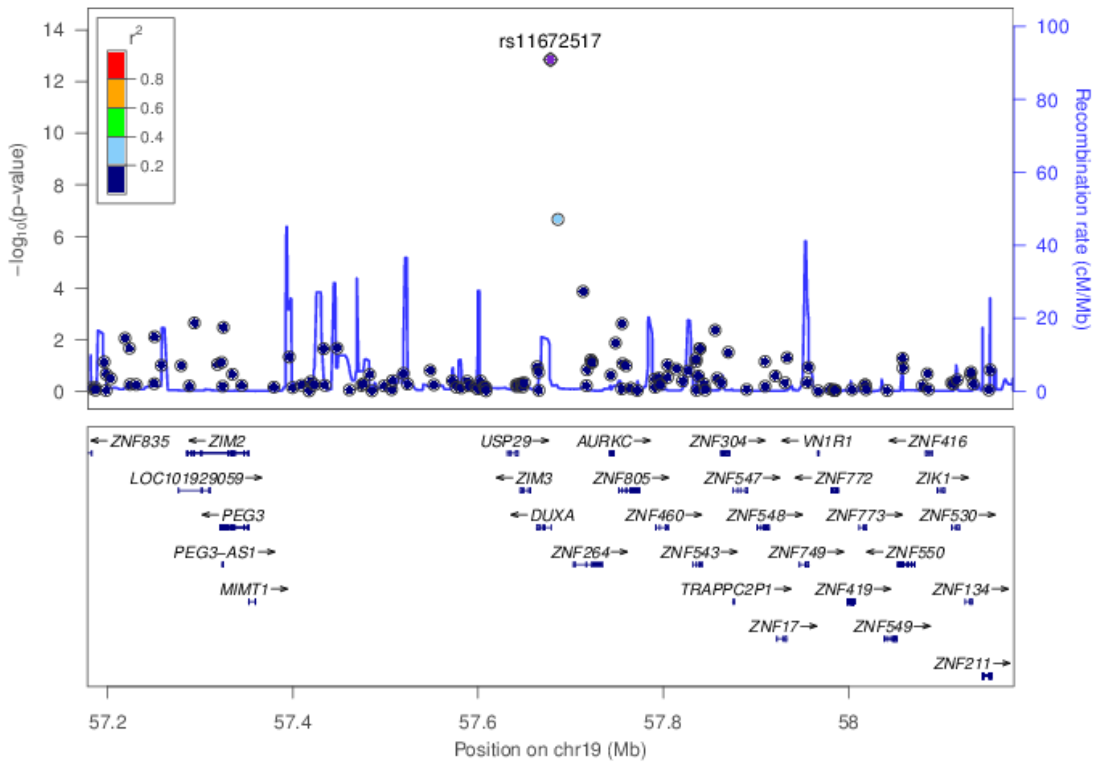
rs11672517
r²
0.8
0.6
0.4
0.2
−log10(p−value)
Recombination rate (cM/Mb)
ZNF835
ZIM2
LOC101929059
PEG3
PEG3−AS1
MIMT1
USP29
ZIM3
DUXA
ZNF264
AURKC
ZNF805
ZNF460
ZNF543
TRAPPC2P1
ZNF304
ZNF547
ZNF548
ZNF749
ZNF17
VN1R1
ZNF772
ZNF773
ZNF419
ZNF549
ZNF416
ZIK1
ZNF530
ZNF550
ZNF134
ZNF211
57.2
57.4
57.6
57.8
58
Position on chr19 (Mb)

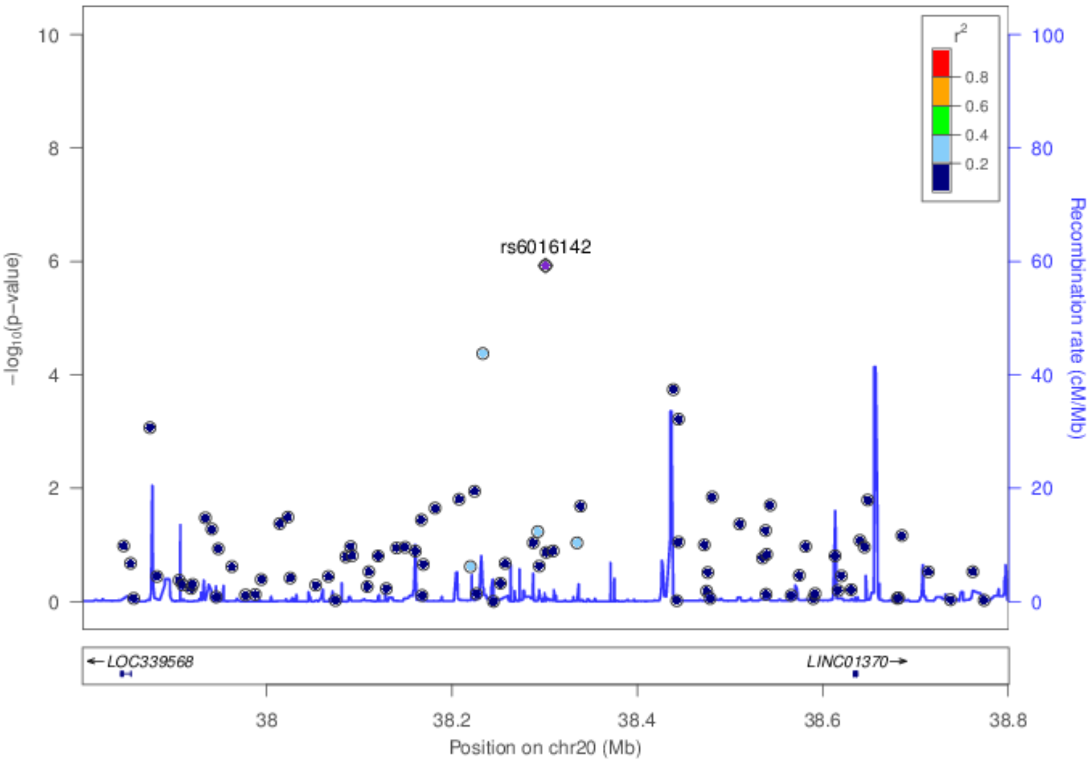
rs6016142
r2
0.8
0.6
0.4
0.2
−log10(p−value)
Recombination rate (cM/Mb)
LOC339568
LINC01370
Position on chr20 (Mb)

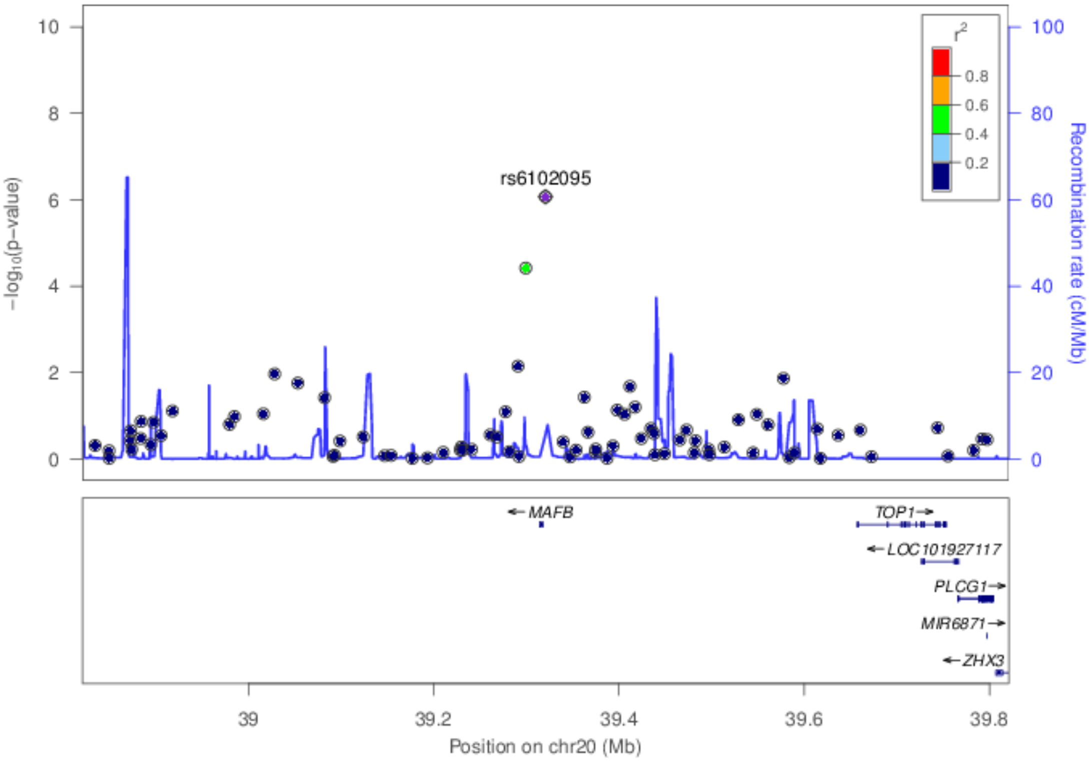
rs6102095
r2
0.8
0.6
0.4
0.2
−log10(p−value)
Recombination rate (cM/Mb)
MAFB
TOP1
LOC101927117
PLCG1
MIR6871
ZHX3
Position on chr20 (Mb)

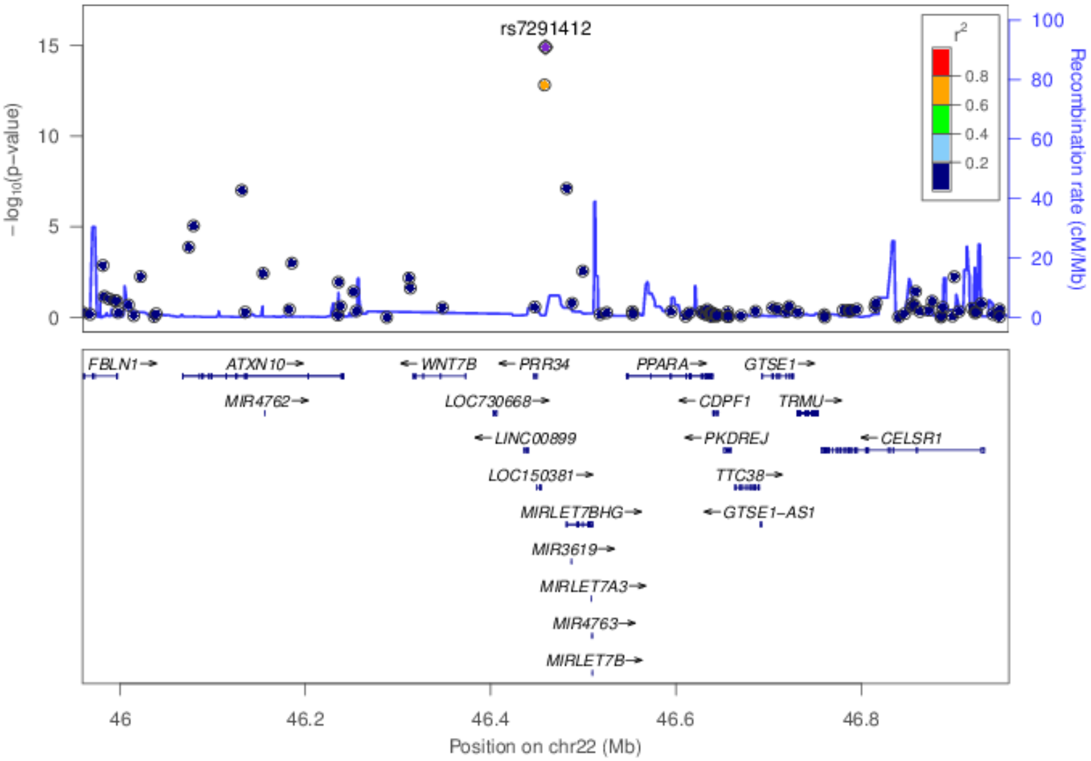

rs7291412
−log10(p−value)
Recombination rate (cM/Mb)
r2
0.8
0.6
0.4
0.2
FBLN1
ATXN10
WNT7B
PRR34
PPARA
GTSE1
MIR4762
LOC730668
CDPF1
TRMU
LINC00899
PKDREJ
CELSR1
LOC150381
TTC38
MIRLET7BHG
GTSE1−AS1
MIR3619
MIRLET7A3
MIR4763
MIRLET7B
Position on chr22 (Mb)

Figure S7. Graphical illustration of the 99% credible sets for all 26 associated regions. For each regional plot, the SNP with the highest posterior probability of association is labeled and depicted with a red diamond. Other SNPs that make up the 99% credible set (Table S2) are depicted with blue diamonds. Imputed SNPs that do not form part of the 99% credible set are depicted with grey circles. Recombination peaks are depicted in light blue and are calculated from HapMap genotypes. The position on each chromosome is shown in relation to Human Genome build hg19.

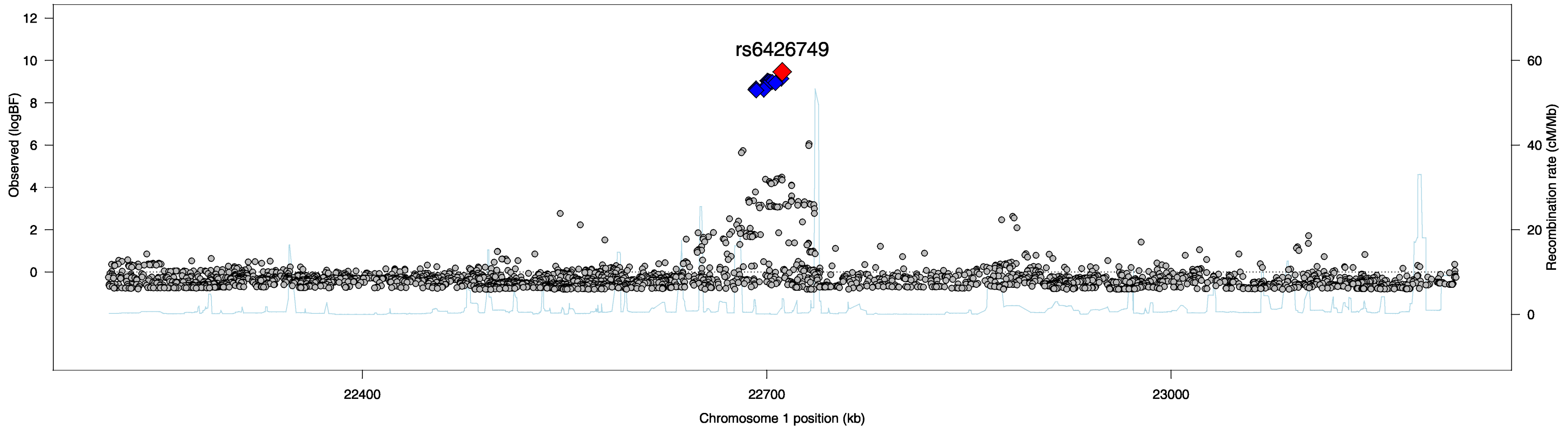
rs6426749
Observed (logBF)
Recombination rate (cM/Mb)
Chromosome 1 position (kb)
22400
22700
23000
0
2
4
6
8
10
12
20
40
60

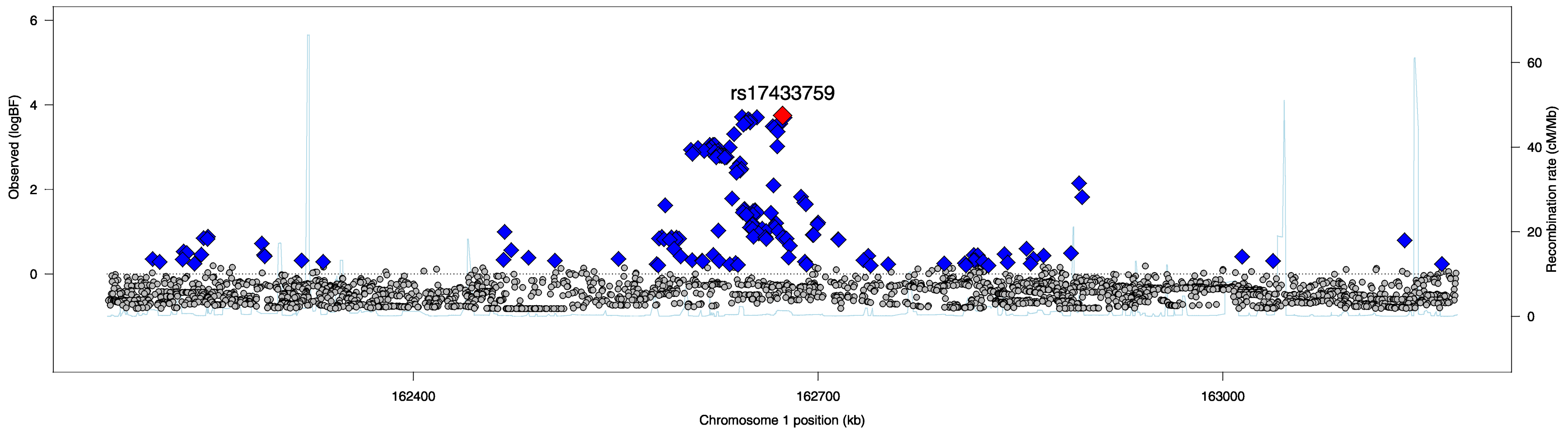
rs17433759
Observed (logBF)
Recombination rate (cM/Mb)
Chromosome 1 position (kb)
162400
162700
163000
0
2
4
6
20
40
60

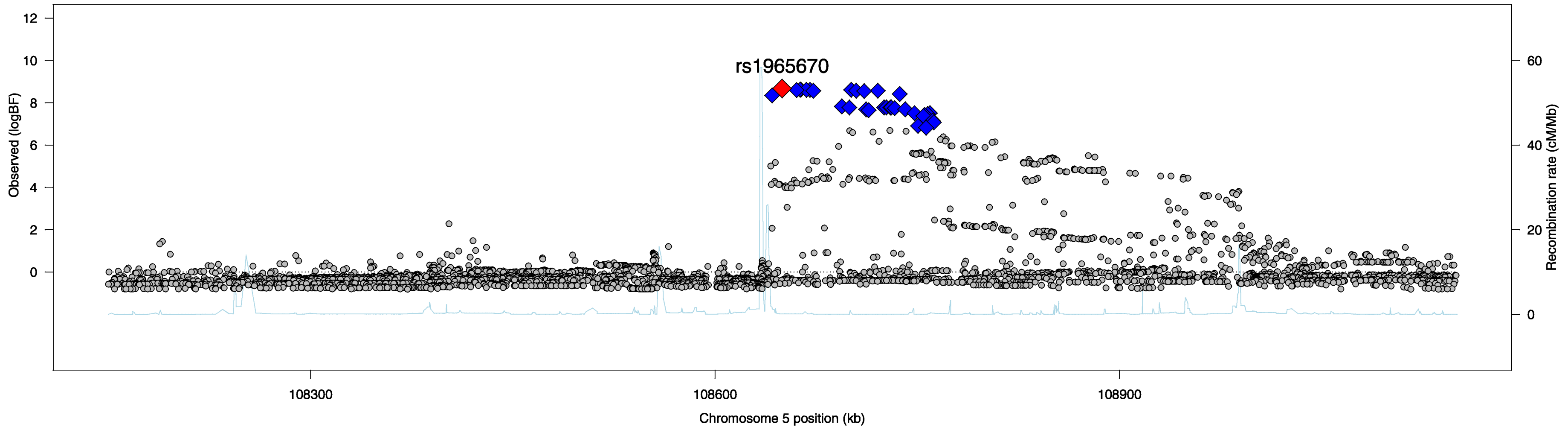

rs1965670
Observed (logBF)
Recombination rate (cM/Mb)
12
10
8
6
4
2
0
60
40
20
0
108300
108600
108900
Chromosome 5 position (kb)

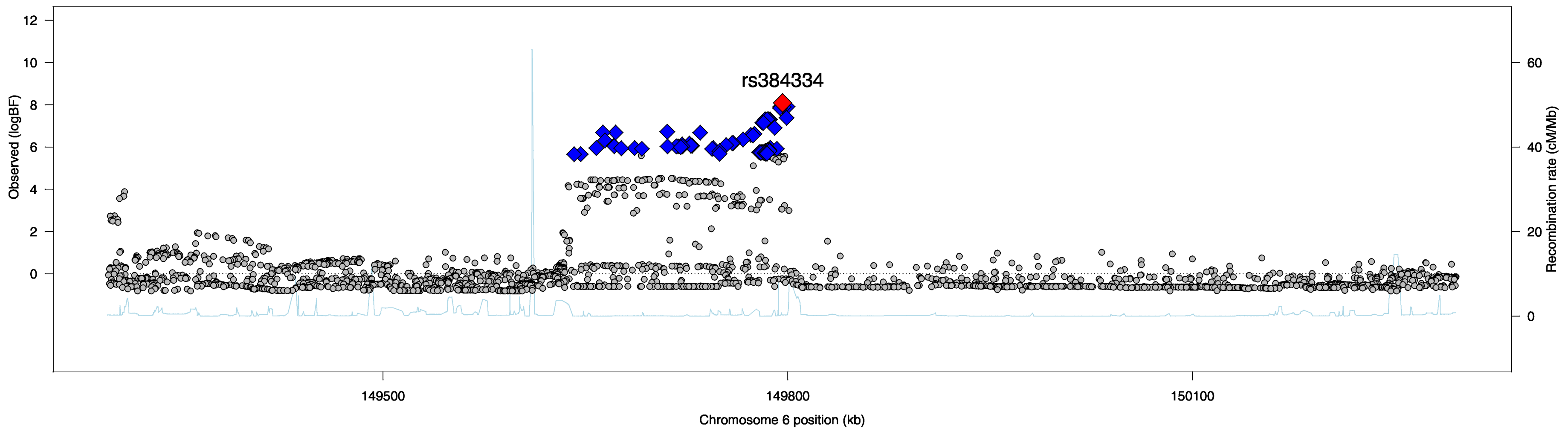

rs384334
Observed (logBF)
Recombination rate (cM/Mb)
12
10
8
6
4
2
0
60
40
20
0
149500
149800
150100
Chromosome 6 position (kb)

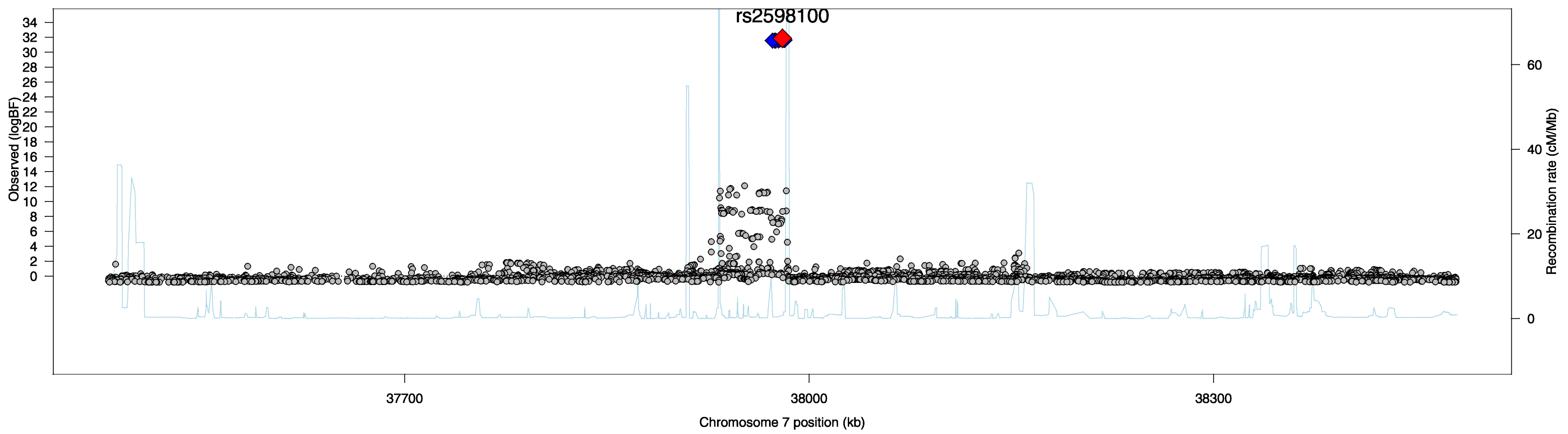

rs2598100
Observed (logBF)
Recombination rate (cM/Mb)
37700
38000
38300
Chromosome 7 position (kb)

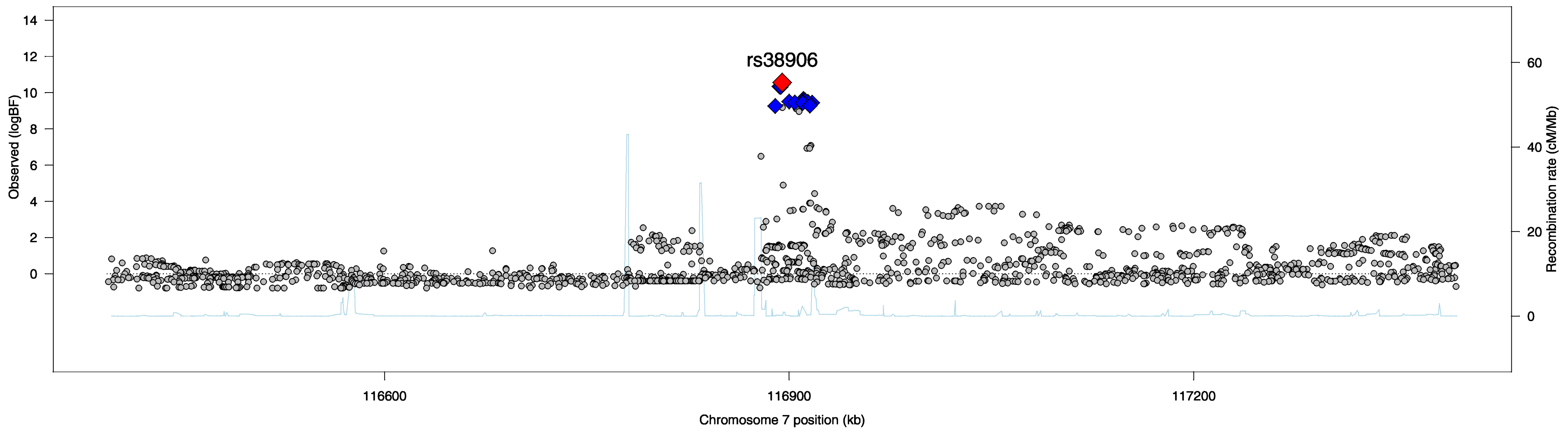

rs38906
Observed (logBF)
Recombination rate (cM/Mb)
116600
116900
117200
Chromosome 7 position (kb)

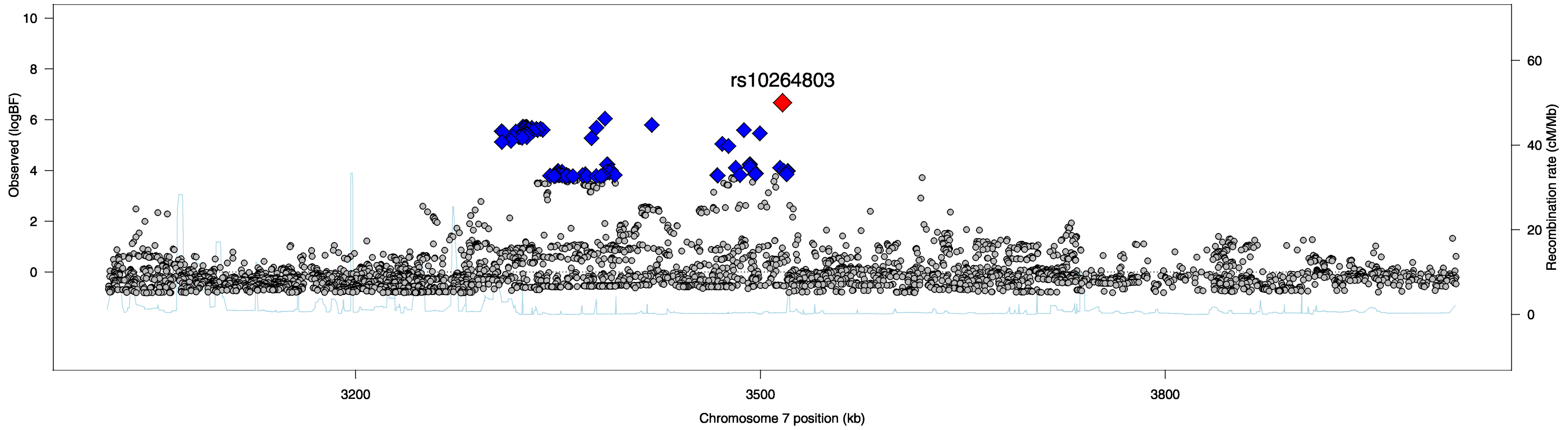

rs10264803
Observed (logBF)
Recombination rate (cM/Mb)
Chromosome 7 position (kb)

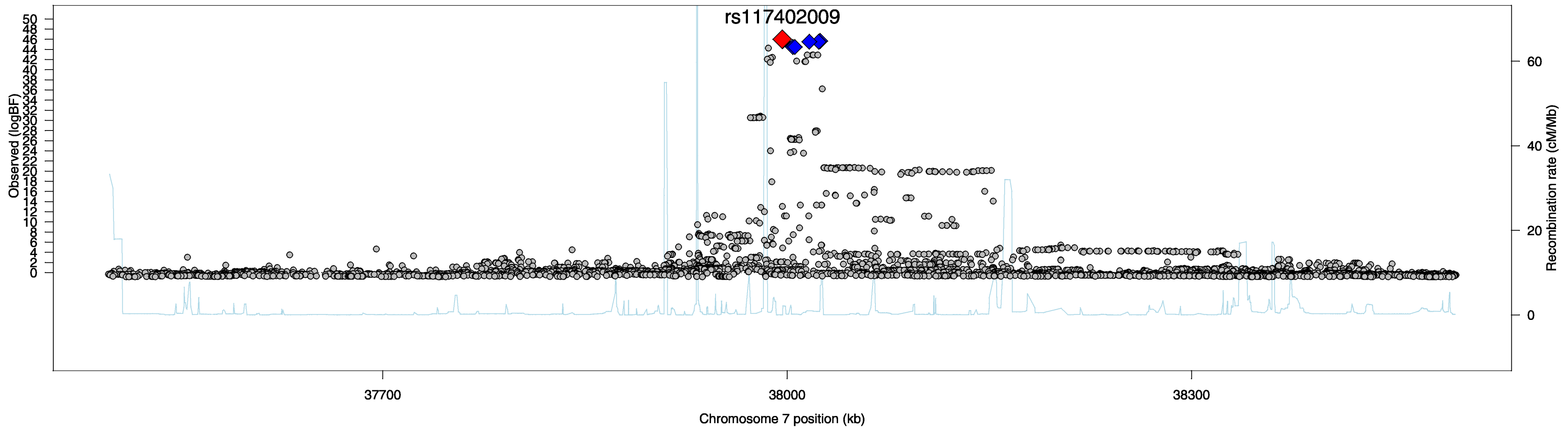

rs117402009
Observed (logBF)
Recombination rate (cM/Mb)
Chromosome 7 position (kb)

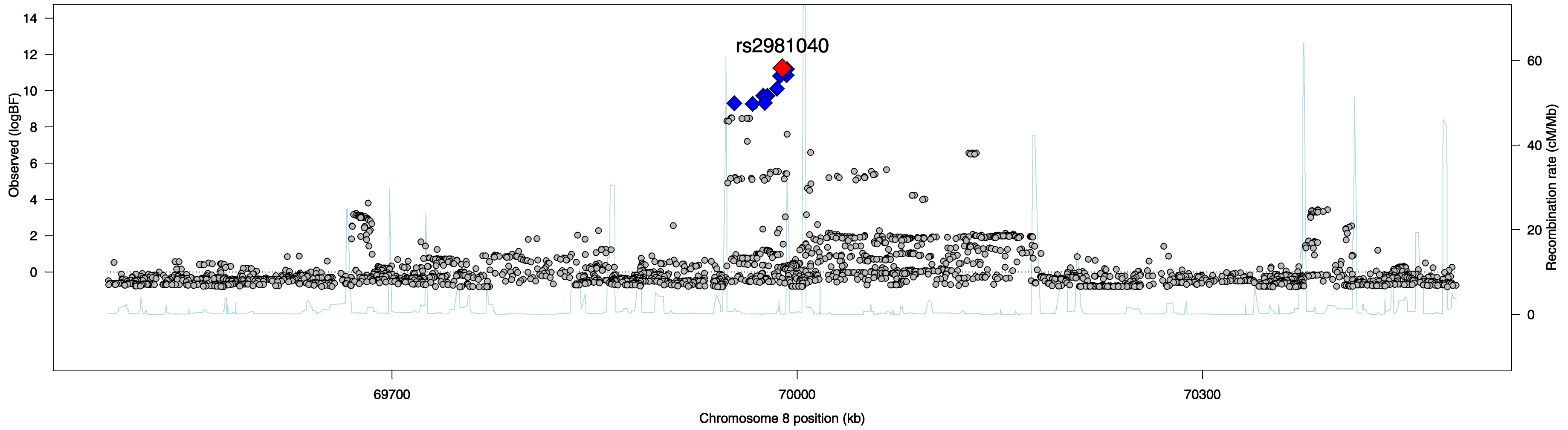

rs2981040
Observed (logBF)
Recombination rate (cM/Mb)
Chromosome 8 position (kb)
69700
70000
70300

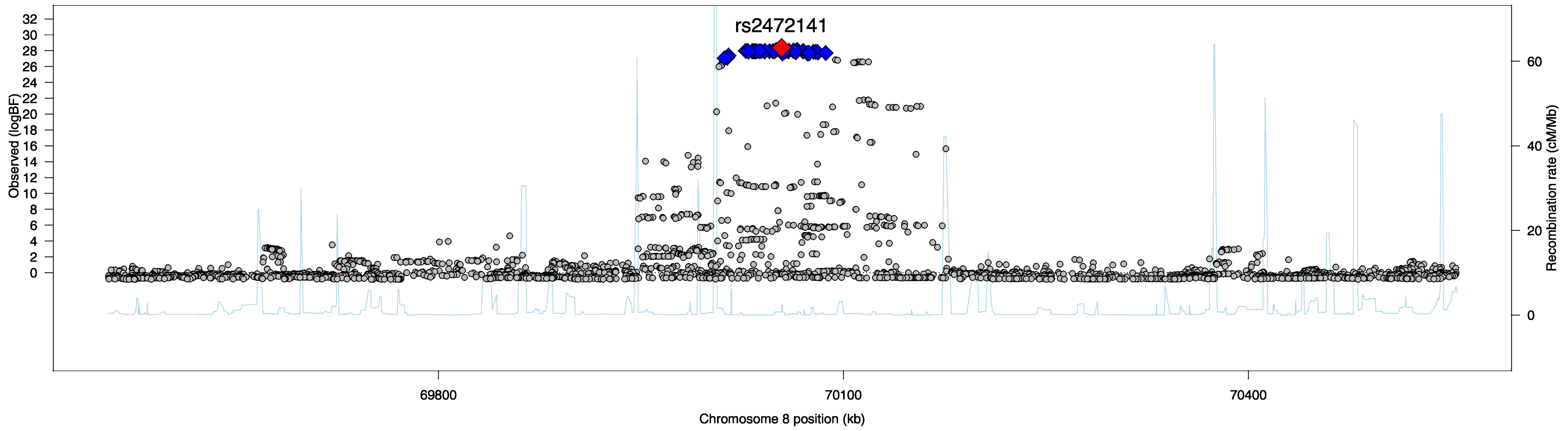

rs2472141
Observed (logBF)
Recombination rate (cM/Mb)
Chromosome 8 position (kb)
69800
70100
70400

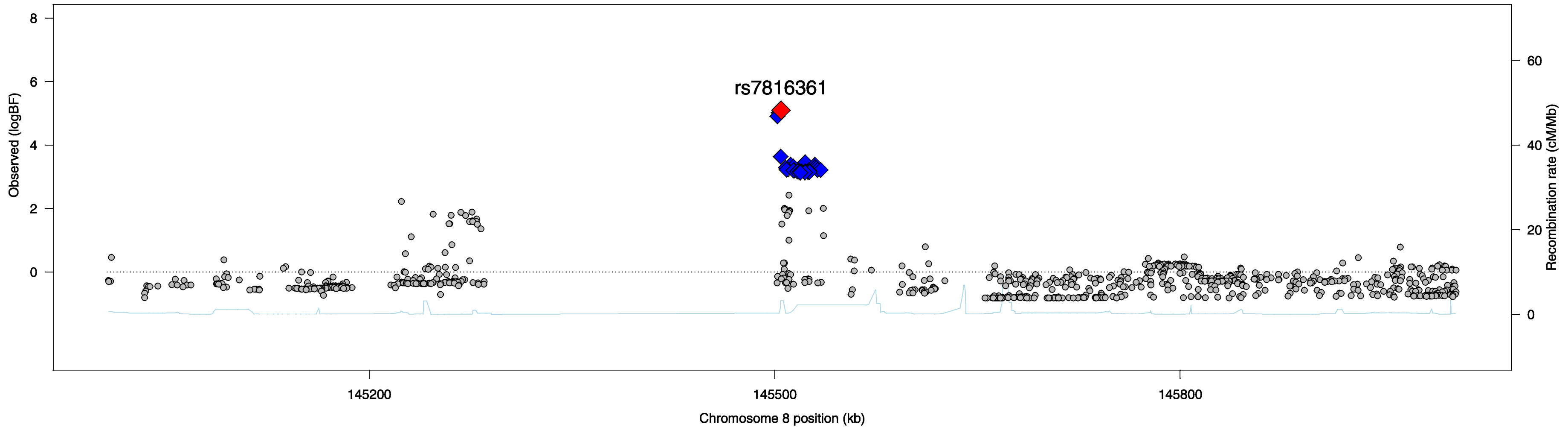
rs7816361
Observed (logBF)
Recombination rate (cM/Mb)
Chromosome 8 position (kb)
145200
145500
145800
8
6
4
2
0
60
40
20
0

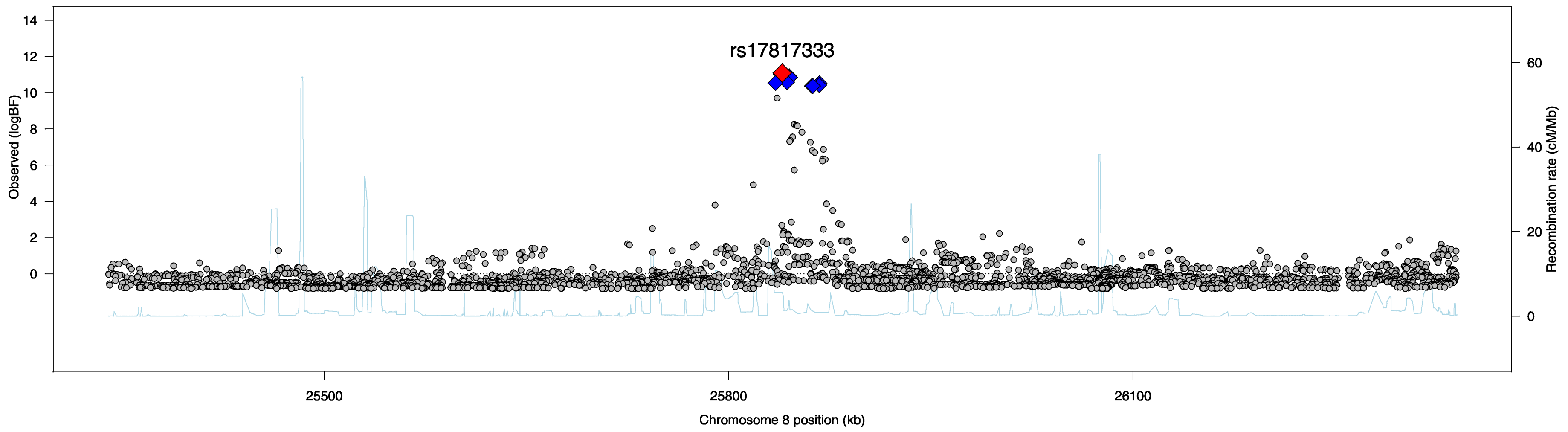
rs17817333
Observed (logBF)
Recombination rate (cM/Mb)
Chromosome 8 position (kb)
25500
25800
26100
14
12
10
8
6
4
2
0
60
40
20
0

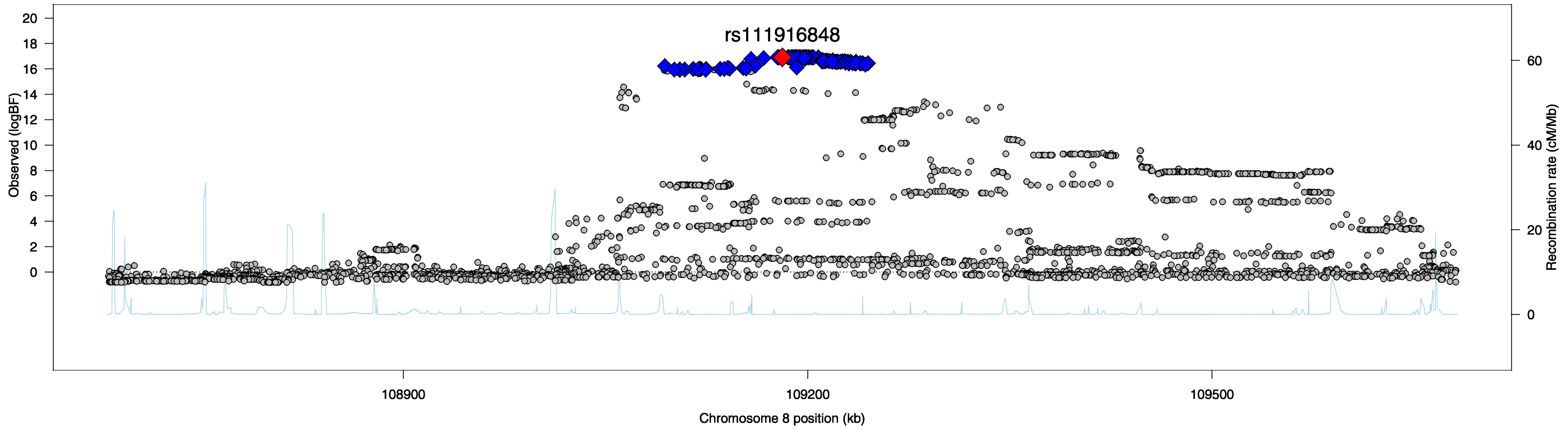

rs111916848
Observed (logBF)
Recombination rate (cM/Mb)
20
18
16
14
12
10
8
6
4
2
0
60
40
20
0
108900
109200
109500
Chromosome 8 position (kb)

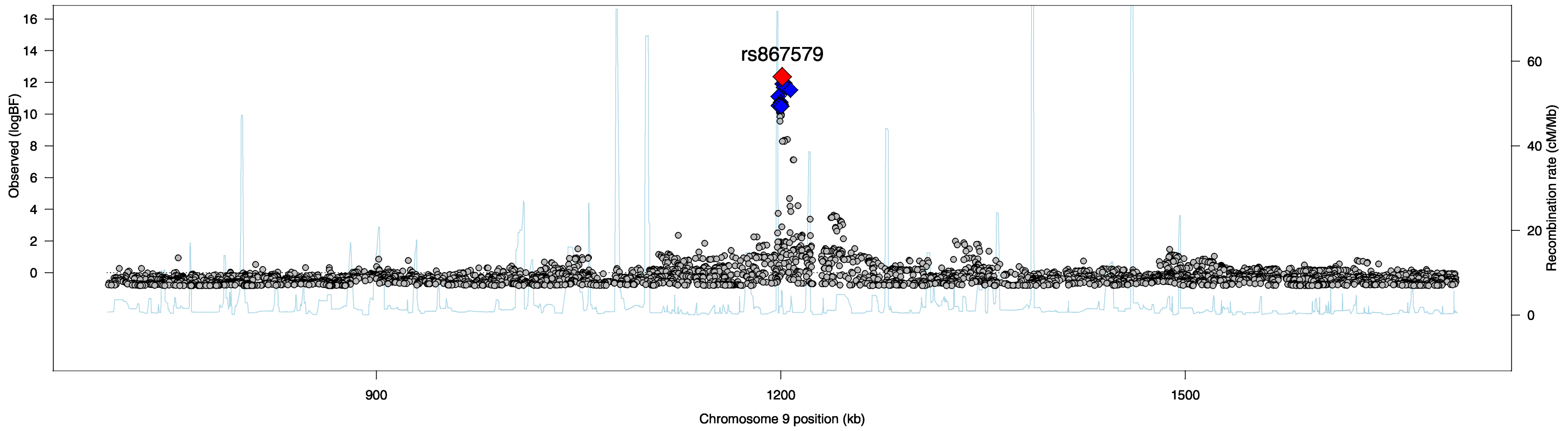

rs867579
Observed (logBF)
Recombination rate (cM/Mb)
16
14
12
10
8
6
4
2
0
60
40
20
0
900
1200
1500
Chromosome 9 position (kb)

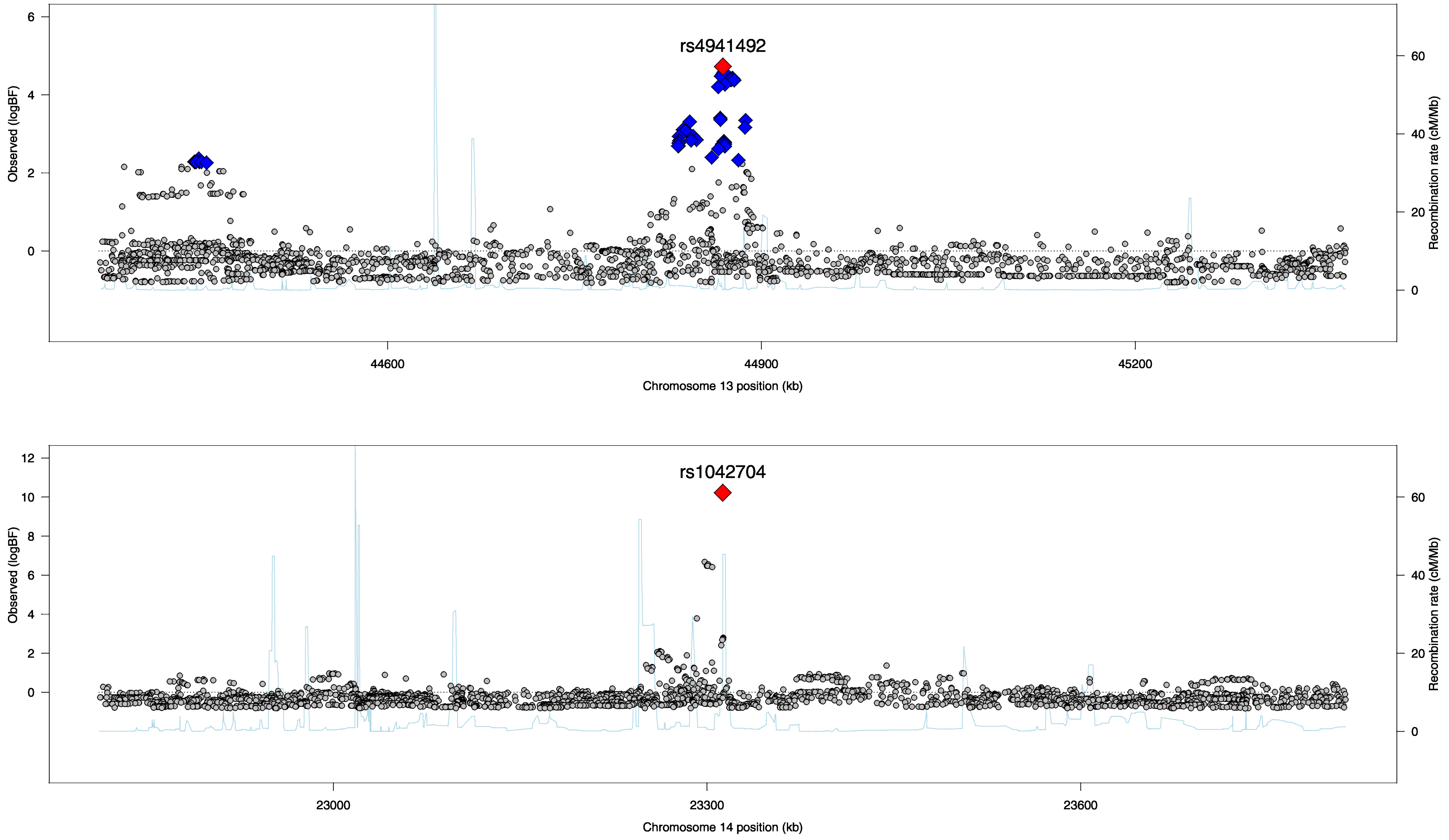

rs4941492
Observed (logBF)
Recombination rate (cM/Mb)
Chromosome 13 position (kb)
44600
44900
45200
rs1042704
Chromosome 14 position (kb)
23000
23300
23600

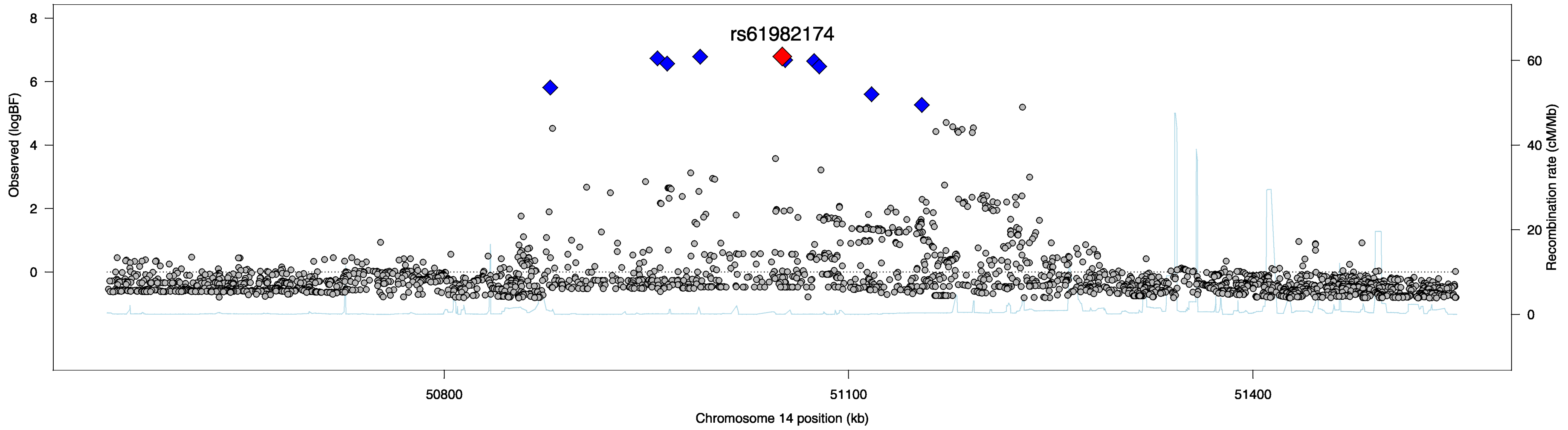
rs61982174
Observed (logBF)
8
6
4
2
0
Recombination rate (cM/Mb)
60
40
20
0
50800
51100
51400
Chromosome 14 position (kb)

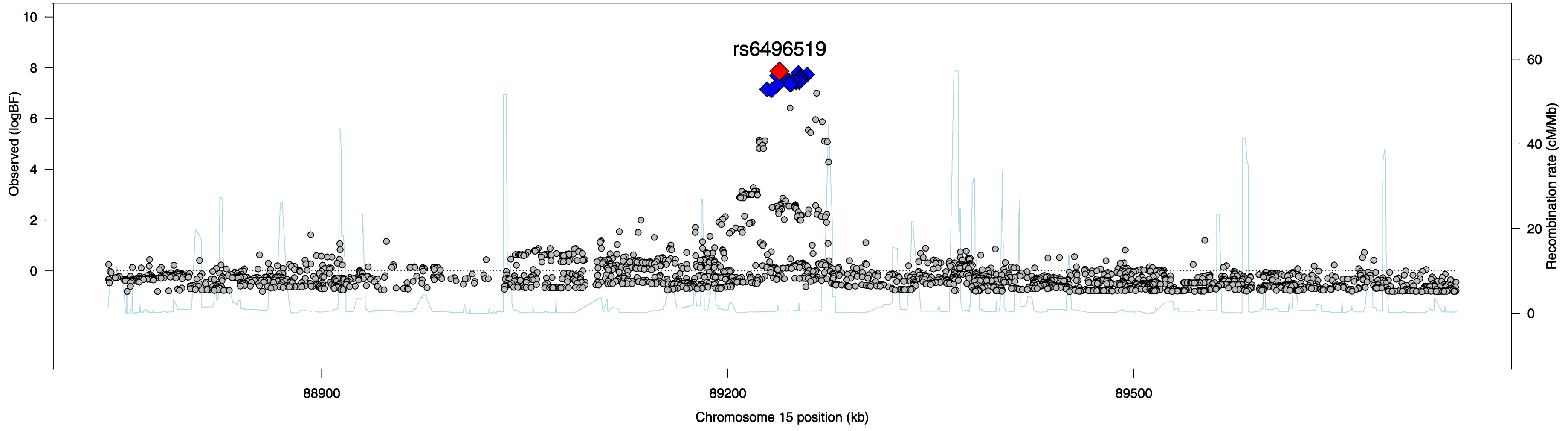
rs6496519
Observed (logBF)
10
8
6
4
2
0
Recombination rate (cM/Mb)
60
40
20
0
88900
89200
89500
Chromosome 15 position (kb)

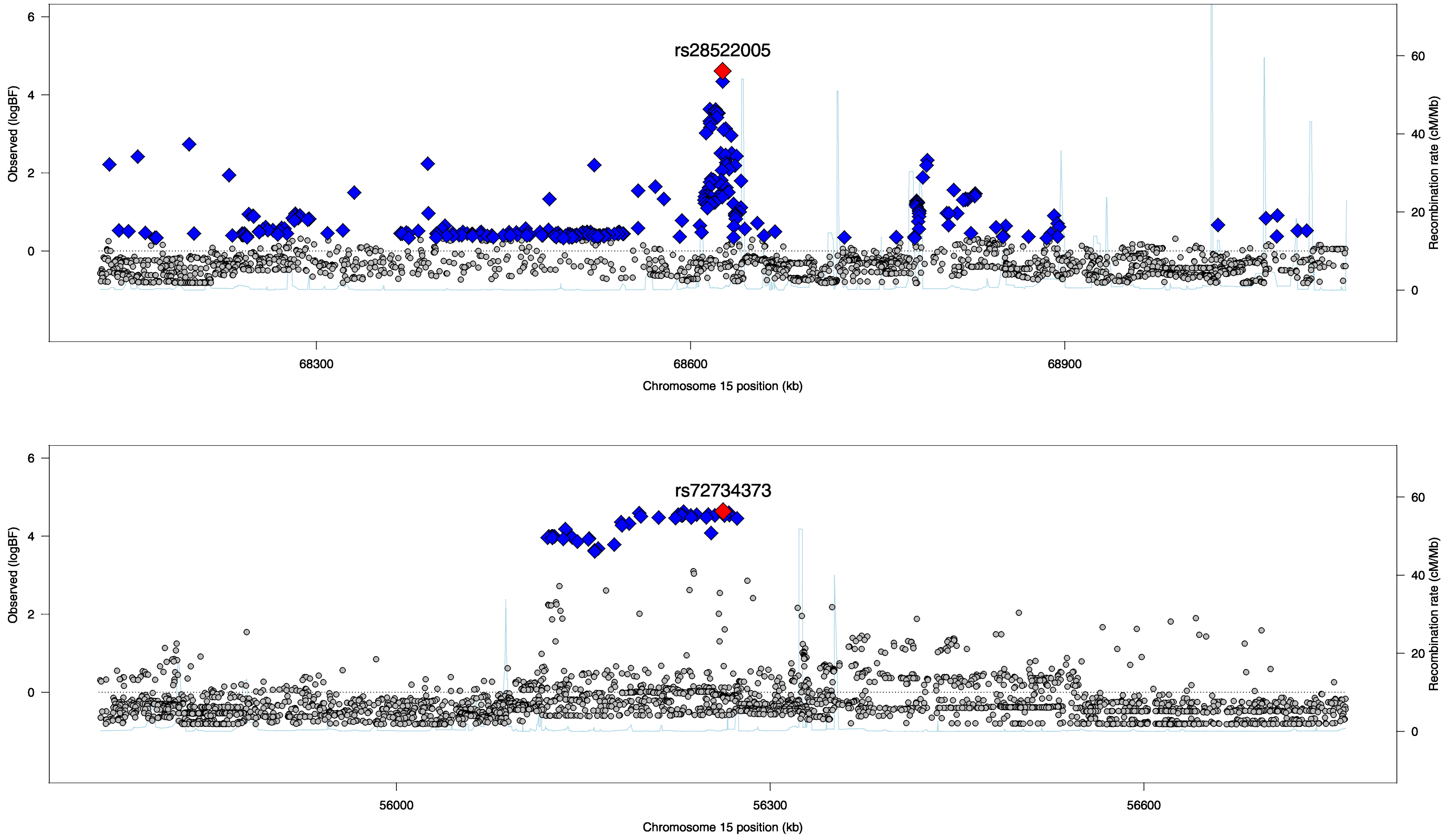

rs28522005
Observed (logBF)
Recombination rate (cM/Mb)
68300
68600
68900
Chromosome 15 position (kb)
rs72734373
Observed (logBF)
Recombination rate (cM/Mb)
56000
56300
56600
Chromosome 15 position (kb)

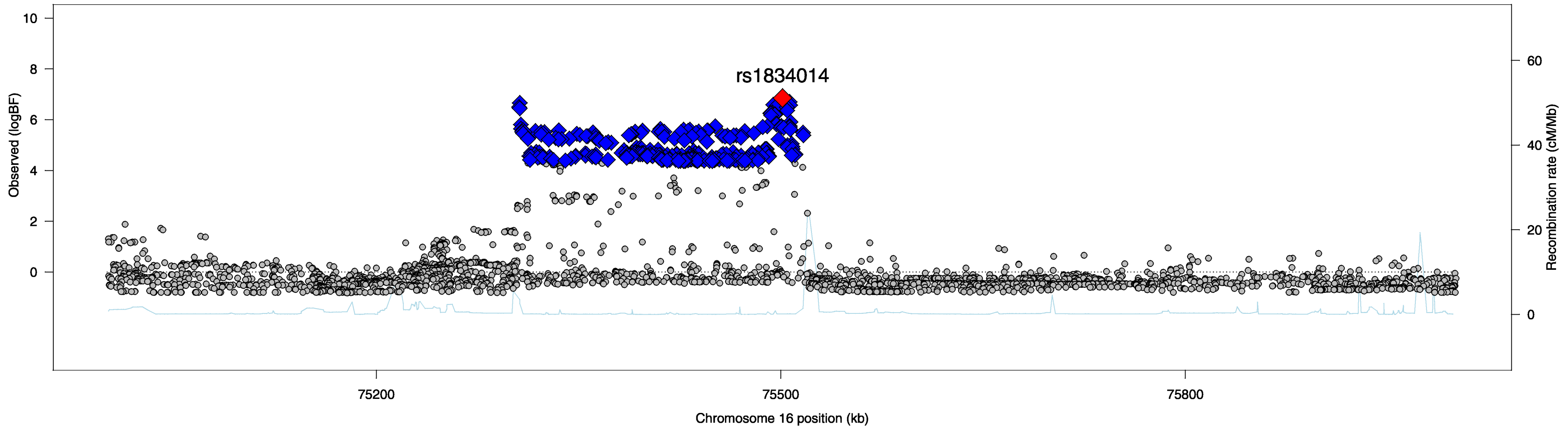
rs1834014
Observed (logBF)
Recombination rate (cM/Mb)
Chromosome 16 position (kb)
75200
75500
75800

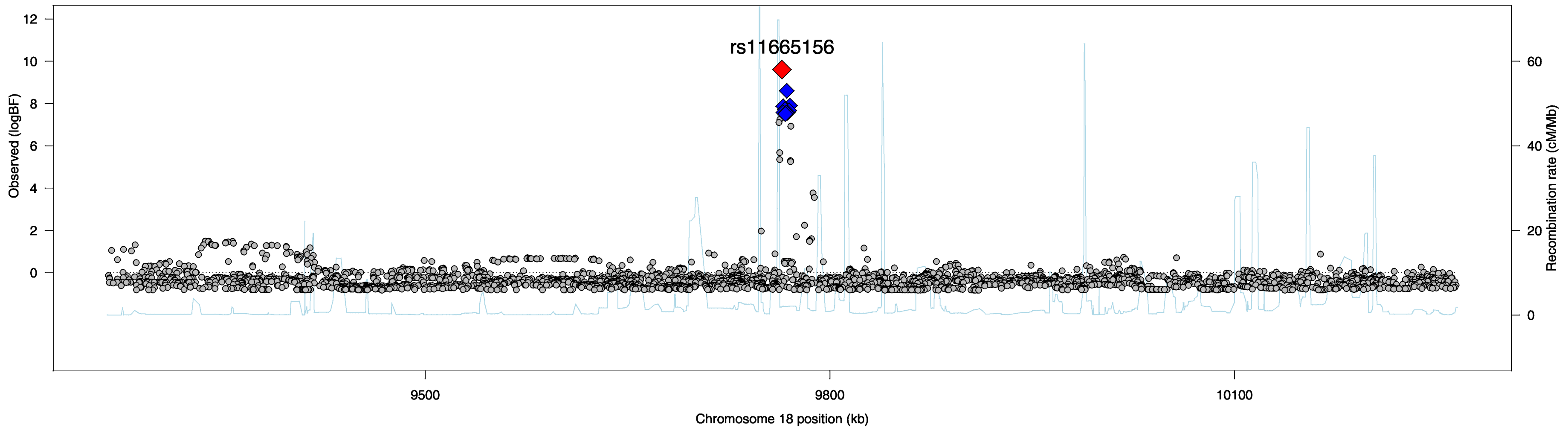
rs11665156
Observed (logBF)
Recombination rate (cM/Mb)
Chromosome 18 position (kb)
9500
9800
10100

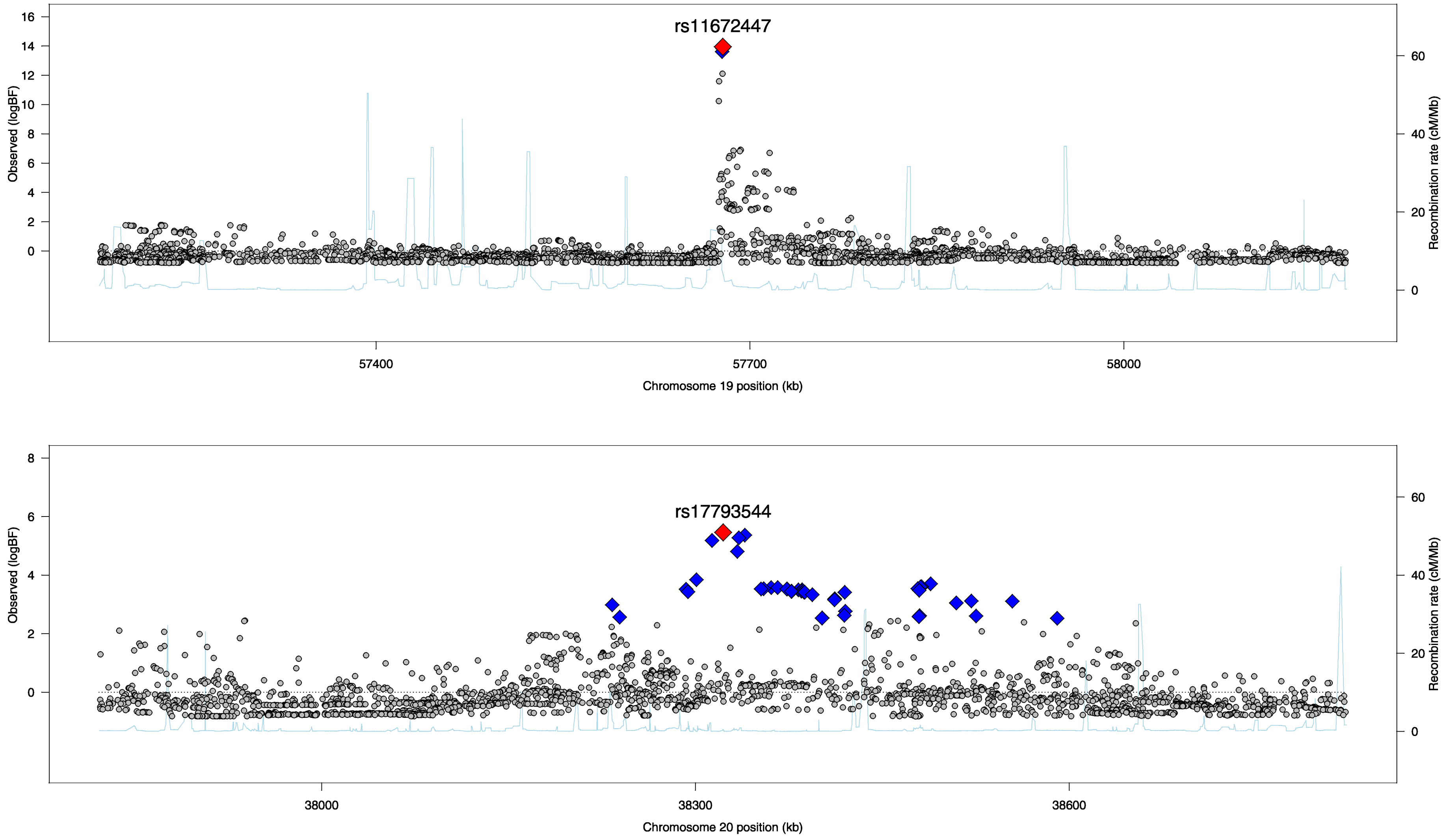

rs11672447
Observed (logBF)
Recombination rate (cM/Mb)
57400
57700
58000
Chromosome 19 position (kb)
rs17793544
Observed (logBF)
Recombination rate (cM/Mb)
38000
38300
38600
Chromosome 20 position (kb)

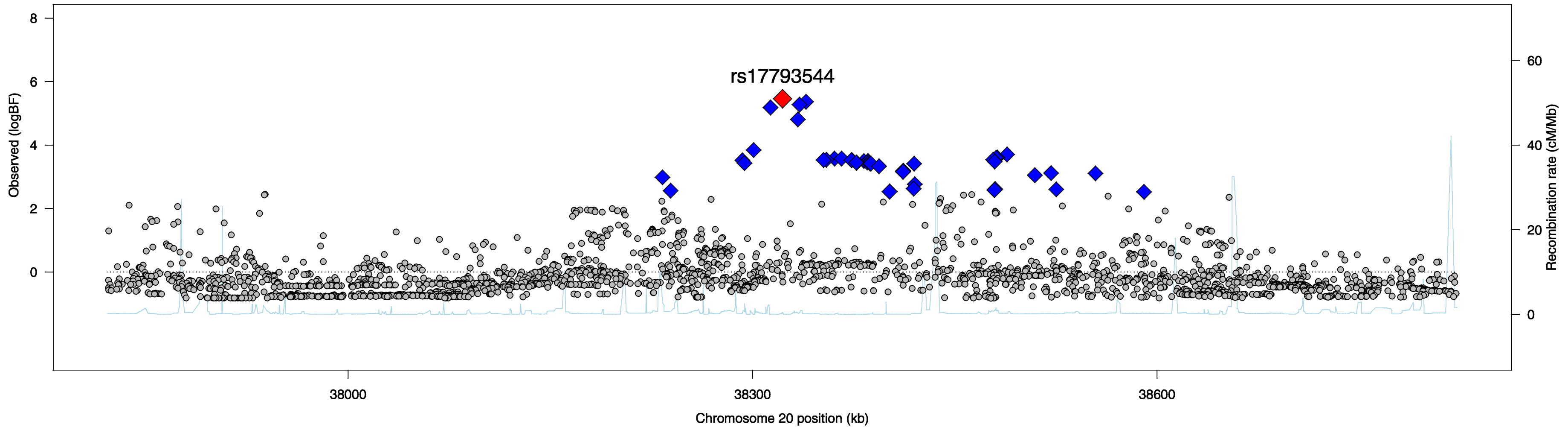
rs17793544
Observed (logBF)
8
6
4
2
0
Recombination rate (cM/Mb)
60
40
20
0
38000
38300
38600
Chromosome 20 position (kb)

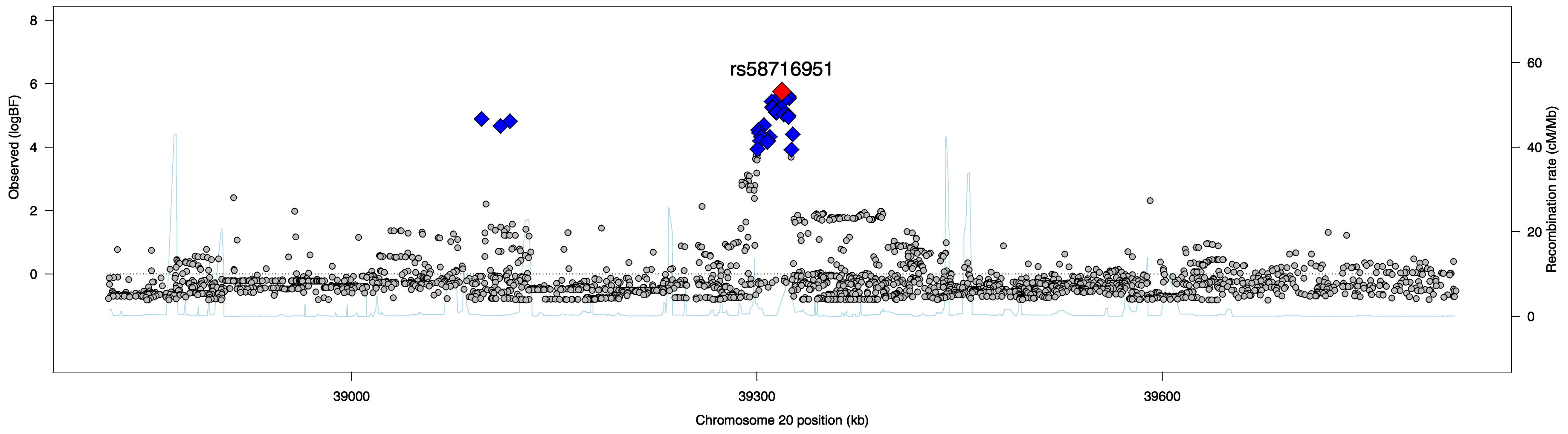
rs58716951
Observed (logBF)
8
6
4
2
0
Recombination rate (cM/Mb)
60
40
20
0
39000
39300
39600
Chromosome 20 position (kb)

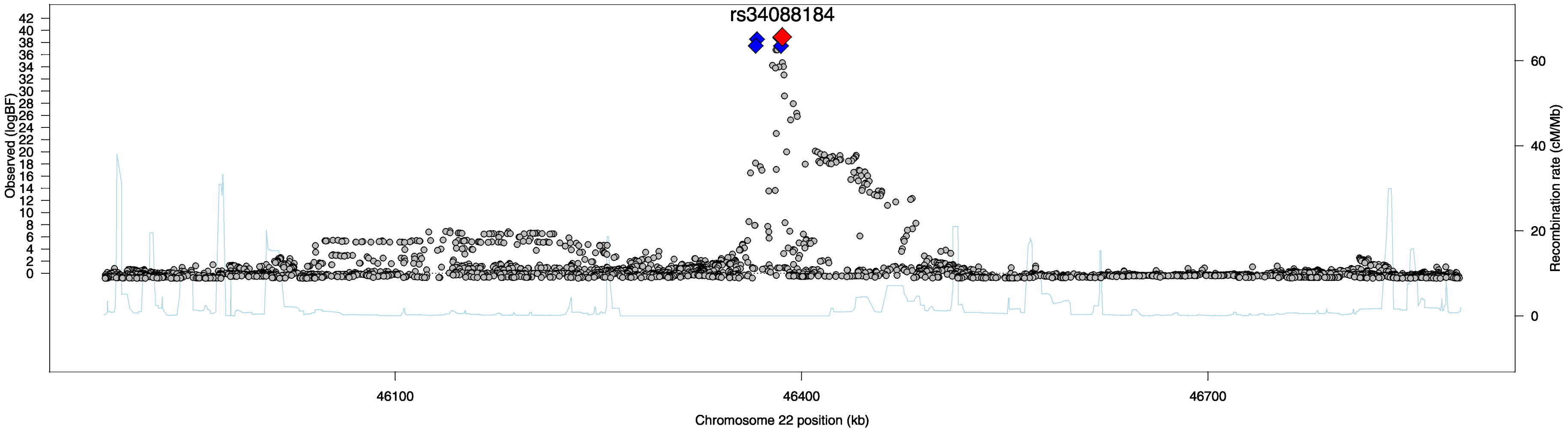

rs34088184
Observed (logBF)
0
2
4
6
8
10
12
14
16
18
20
22
24
26
28
30
32
34
36
38
40
42
Recombination rate (cM/Mb)
0
20
40
60
46100
46400
46700
Chromosome 22 position (kb)

Figure S8. Immunohistochemistry of fixed, surgically resected fibrotic DD tissue confirms WNT3A expression (brown, upper left panel) compared to isotype control (brown, upper right panel), and palm skin (brown, lower left panel). SFRP4 was also not expressed in palm skin (brown, lower right panel). Scale bar = 20µm.

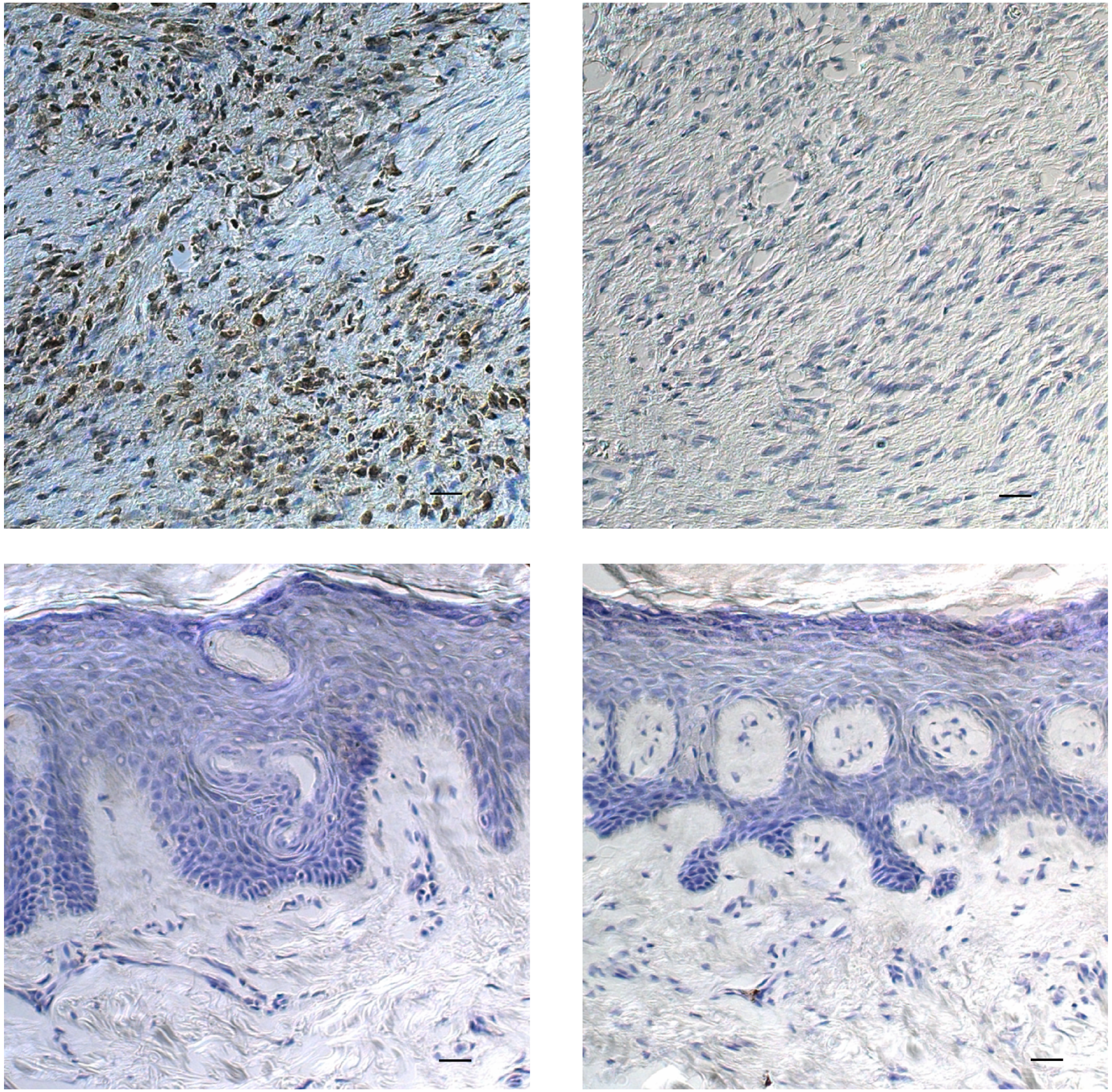

Figure S9. Annotated regional association plots for a) rs12677559 and b) rs2247056 after analysis of the discovery cohort, plotted with LocusZoom [1]. Recombination rate was calculated within the software using data from HapMap. In the lower panel of each figure, genes within 500kb of the index SNP are shown. The position on the chromosomes is shown in relation to Human Genome build hg19.

a)

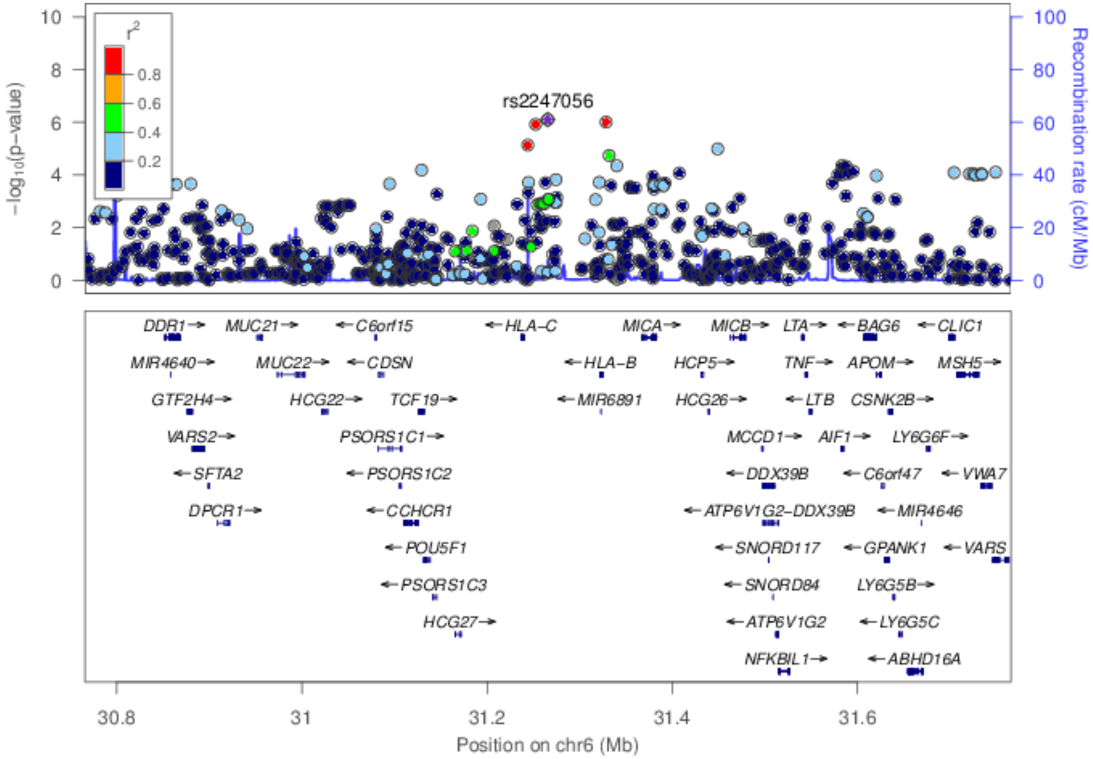

b)

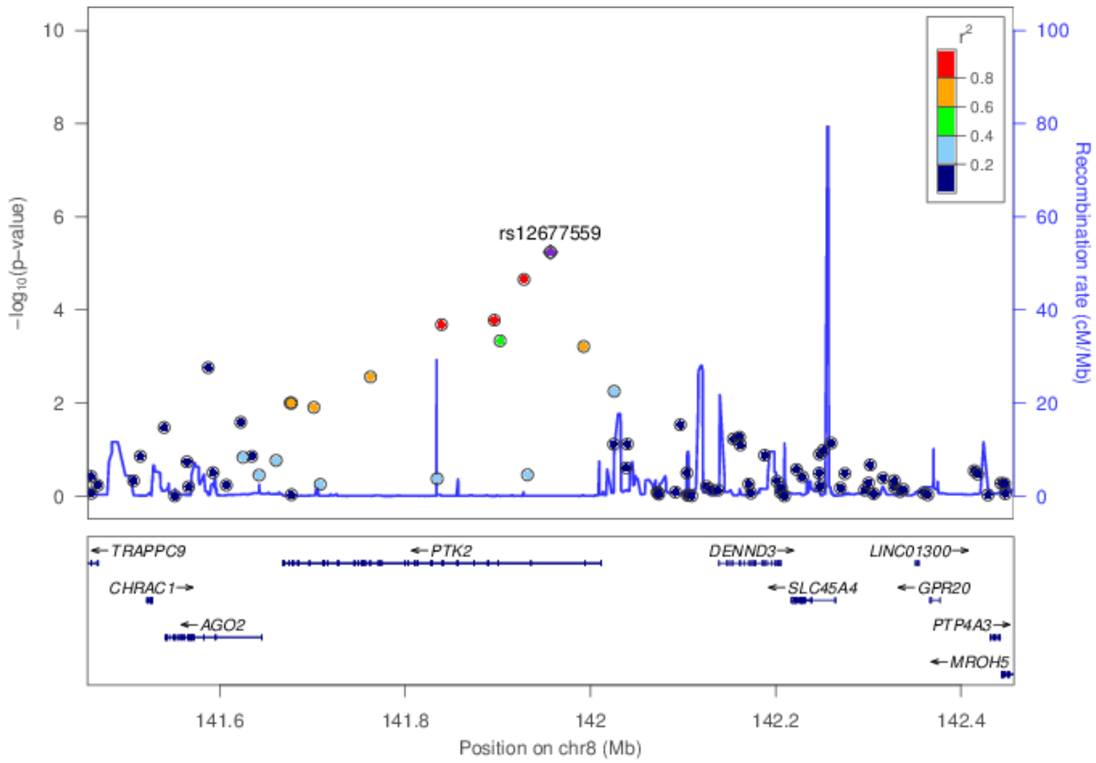

rs12677559
r2
0.8
0.6
0.4
0.2
−log10(p−value)
Recombination rate (cM/Mb)
0
2
4
6
8
10
0
20
40
60
80
100
TRAPPC9
PTK2
DENND3
LINC01300
CHRAC1
SLC45A4
GPR20
AGO2
PTP4A3
MROH5
141.6
141.8
142
142.2
142.4
Position on chr8 (Mb)

Table S1. Samples and genotyping platforms used in the discovery and replication phases

			Pre-QC		Post-QC	
Phase	Geographical Origin	Genotyping Platform	Cases	Controls	Cases	Controls
Discovery	United Kingdom	Illumina HumanCoreExome	4201	4891	3871	4686
Replication	Germany	Taqman<br>Affymetrix 6.0<br>Illumina Axiom	768	1353	768	1321
	Netherlands	Sequenom Custom array	2195	1983	2104	1860
	United Kingdom	Sequenom Custom array<br>Illumina HumanCoreExome	1207	5070	1169	5070

Table S2. 99% credible sets for associated loci. This table is supplied as an Excel workbook, with a separate worksheet for each individual credible set.

Table S3. Variants carried through to replication phase, if an assay could be designed. Variants that reached genome-wide significance ($p>5x10^{-8}$) on meta-analysis of discovery and replication cohorts are also reported in Table 1 in the main manuscript. This table is supplied as an Excel workbook.
